# Dynamic cell contacts between periportal mesenchyme and ductal epithelium act as a rheostat for liver cell proliferation

**DOI:** 10.1016/j.stem.2021.07.002

**Published:** 2021-11-04

**Authors:** Lucía Cordero-Espinoza, Anna M. Dowbaj, Timo N. Kohler, Bernhard Strauss, Olga Sarlidou, German Belenguer, Clare Pacini, Nuno P. Martins, Ross Dobie, John R. Wilson-Kanamori, Richard Butler, Nicole Prior, Palle Serup, Florian Jug, Neil C. Henderson, Florian Hollfelder, Meritxell Huch

**Affiliations:** 1 Wellcome Trust/Cancer Research UK Gurdon Institute, Cambridge CB2 1QN, UK; 2 Wellcome Trust–Medical Research Council Stem Cell Institute, Cambridge CB2 1QR, UK; 3 Department of Physiology, Development and Neuroscience, University of Cambridge, Cambridge CB2 3DY, UK; 4 Max Planck Institute of Molecular Cell Biology and Genetics, Dresden 01307, Germany; 5 Department of Biochemistry, University of Cambridge, Cambridge CB2 1GA, UK; 6 Wellcome Sanger Institute, Wellcome Genome Campus, Hinxton, Cambridge CB10 1SA, UK; 7 Centre for Inflammation Research, The Queen’s Medical Research Institute, University of Edinburgh, Edinburgh EH16 4TJ, UK; 8 MRC Human Genetics Unit, Institute of Genetics and Cancer, University of Edinburgh, Edinburgh EH4 2XU, UK; 9 Novo Nordisk Foundation Center for Stem Cell Biology (DanStem), University of Copenhagen, Copenhagen 2200, Denmark

**Keywords:** liver, organoid, niche, mesenchyme, regeneration, multicellular co-culture, droplet microfluidics, flow-focussing device, organotypic co-culture, liver ductal cell

## Abstract

In the liver, ductal cells rarely proliferate during homeostasis but do so transiently after tissue injury. These cells can be expanded as organoids that recapitulate several of the cell-autonomous mechanisms of regeneration but lack the stromal interactions of the native tissue. Here, using organoid co-cultures that recapitulate the ductal-to-mesenchymal cell architecture of the portal tract, we demonstrate that a subpopulation of mouse periportal mesenchymal cells exerts dual control on proliferation of the epithelium. Ductal cell proliferation is either induced and sustained or, conversely, completely abolished, depending on the number of direct mesenchymal cell contacts, through a mechanism mediated, at least in part, by Notch signaling. Our findings expand the concept of the cellular niche in epithelial tissues, whereby not only soluble factors but also cell-cell contacts are the key regulatory cues involved in the control of cellular behaviors, suggesting a critical role for cell-cell contacts during regeneration.

## Highlights

• The numbers of mesenchyme-ductal cell contacts change during damage regeneration
• Ductal-mesenchyme organoids recapitulate cell interactions and *in vivo* architecture
• Mesenchymal cells induce ductal epithelium cell proliferation via soluble factors
• Mesenchymal cells impair ductal epithelium cell proliferation via cell-cell contacts

## Graphical abstract

**Figure:**
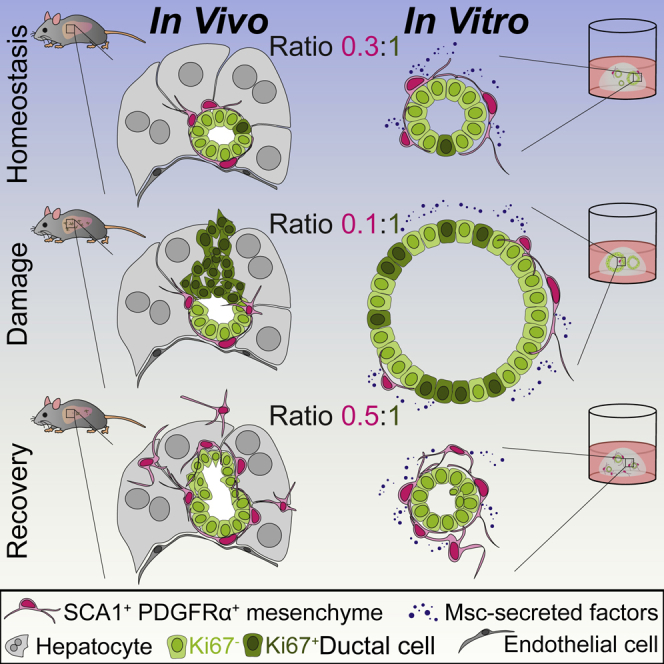

## Teaser

Huch and colleagues describe that during liver regeneration, ductal and mesenchymal cells proliferate with different tempos, resulting in changes in the cellular contacts between both populations. By developing organoid co-cultures that recapitulate ductal-mesenchymal interactions, they provide proof that the number of cell contacts matters and either promotes or arrests epithelial proliferation.

## Introduction

The adult liver epithelium comprises hepatocytes and biliary ducts lined by liver ductal cells (DCs, also known as cholangiocytes). The epithelium is mostly mitotically dormant in homeostasis yet proliferates swiftly upon damage, enabling rapid regeneration (Miyajima et al., 2014). Although hepatocytes comprise the bulk of the regenerative response (Malato et al., 2011), DCs also respond to injury (Furuyama et al., 2011). In addition, severe tissue damage and hepatocyte senescence inducecellular plasticity in the ductal compartment and endow the otherwise unipotent cholangiocytes with the capacity to replace lost hepatocyte mass (Choi et al., 2014; Raven et al., 2017). Healthy adult DCs can be expanded *in vitro* as self-renewing liver organoids in a 3D extracellular matrix (Matrigel) and a defined cocktail of growth factors (R-Spondin-1 [RSPO1], Fibroblast Growth Factor 10 [FGF10], epidermal growth factor [EGF], and Hepatocyte Growth Factor [HGF]; Huch et al., 2013, 2015) that recapitulate the transient mitogenic milieu of the regenerating liver (Apte et al., 2008). Using this model system, we have shown that liver ductal organoids recapitulate many aspects of liver regeneration in a dish (Aloia et al., 2019). Notwithstanding, across multiple mammalian tissues, regeneration relies on the dynamic crosstalk between the epithelium and its respective tissue microenvironment (Gurtner et al., 2008). The contribution of the latter in the ductal-mediated regeneration of the liver are largely unknown.

The patterning of hepatic epithelium throughout development is dependent on cues from apposed mesenchymal tissues (Zaret, 2002). In the adult liver, the hepatic mesenchymal pool, whose ontogeny traces back to the septum transversum mesenchyme [STM] (Zorn, 2008), diversifies into centro-lobular fibroblasts and smooth muscle cells, lobule-interspersed hepatic stellate cells (HSCs), and a portal tract (PT)-restricted population referred to as portal fibroblasts (PFs) (Lepreux and Desmoulière, 2015). The physiology of these cells has been appraised in the context of various disease states (Mederacke et al., 2013; Ramachandran et al., 2019). *In vivo* inhibition of HSC activation exacerbates liver damage while diminishing DC expansion (Pintilie et al., 2010; Shen et al., 2011). Similarly, Thy1^+^ HSCs and PFs have been identified as a source of Fibroblast Growth Factor 7 [FGF7] that sustains DC proliferation during regeneration (Takase et al., 2013), while Jagged1^+^ myofibroblasts direct ductal lineage differentiation in mouse models of chronic liver damage (Boulter et al., 2012). Although these studies highlight discrete cases of mesenchymal-to-ductal cell signaling, they do not address the dynamic interactions between these lineages in homeostasis or throughout the different phases of the regenerative response. *In vitro* co-culture models with different stromal populations have been devised to enhance the stability and functions of hepatocytes (Berger et al., 2015; Bhatia et al., 1998; Coll et al., 2018; Davidson et al., 2017; Nguyen et al., 2015; Ouchi et al., 2019; Taymour et al., 2021; Ware et al., 2017). However, ductal-mesenchymal co-cultures have not been reported yet. In addition, current *ex vivo* adult liver organoid models are epithelial centric and fail to recapitulate the multicellular complexity of the adult tissue (Prior et al., 2019), hampering an in-depth understanding of the stromal niche-to-epithelial cell interactions during homeostasis and regeneration.

Here, we describe that a subpopulation of peri-portal mesenchymal cells (labeled by platelet-derived growth factor receptor alpha [PDGFRα] and stem cell antigen 1 [SCA1]) acts as a rheostat that regulates the proliferation capacity of DCs. Mesenchymal-secreted mitogens support organoid formation and expansion. However, direct mesenchymal-to-ductal cell contact abolishes DC proliferation in a mesenchyme-dose-dependent manner through mechanisms that involve, at least in part, Notch signaling activation. Hence, our results indicate that the number of cellular contacts between epithelium and mesenchyme, rather than the absolute number of cells in both populations, controls DC proliferation dynamics.

## Results

### Periportal PDGFRα^+^SCA1^+^ mesenchymal cells surrounding the duct epithelium express a pro-regenerative growth factor signature

**Figure 1:**
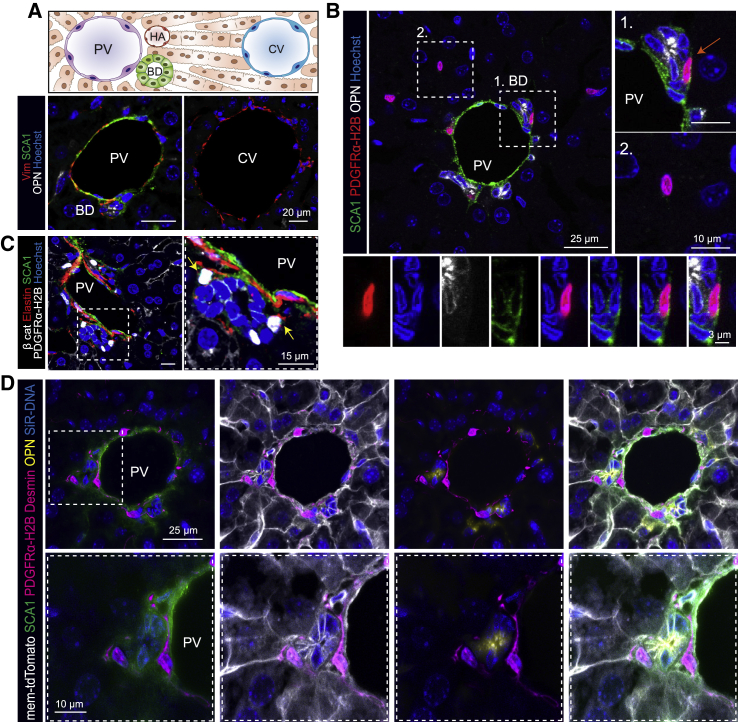
PDGFRα^+^SCA1^+^ mesenchymal cells reside near the portal tract (A) SCA1 marks exclusively the portal tract region of the liver lobule. Top: schematic of a liver lobule, spanning from the portal tract (formed by the portal vein [PV], hepatic artery [HA], and bile duct [BD]) to the central vein (CV) area. Bottom: representative composite single z stack images of liver sections, stained for SCA1 (green), Vimentin (Vim, red), the ductal marker osteopontin (OPN, white) and nuclei (Hoechst, blue). (B) Representative composite single z stack images of *Pdgfra-H2B-GFP* (nuclear red) mouse livers co-stained with SCA1 (green) and the ductal marker osteopontin (OPN, white). PDGFRα^+^SCA1^+^ cells (B1, close up underneath) are in close proximity to the ductal epithelium (orange arrow), while PDGFRα^+^SCA1^−^ cells (B2) are spread throughout the parenchyma. (C) Representative composite single z stack images of *Pdgfra-H2B-GFP* (nuclear white) mouse livers co-stained with SCA1 (green), elastin (red), and β-catenin (white, membrane). Yellow arrow, PDGFRα^+^SCA1^+^. (D) Representative composite maximum intensity projection image of PDGFRα^+^SCA1^+^ cells (nuclear magenta) contacting liver ductal cells (OPN, yellow) through desmin (magenta) membrane protrusions; SCA1 staining (green), membrane marker tdTomato (white), and DNA (SiR-DNA, blue). See also Figure S1 and Video S1.

The process of tissue regeneration is a joint endeavor between the epithelium and surrounding stroma. Accordingly, we first sought to characterize the proximate neighbors of the ductal epithelium, which we hypothesized could act as a regulatory niche for DC-driven regeneration. Liver ductal cells (DCs; also known as cholangiocytes) reside at the PT area of the liver lobule (Figure 1A), spatially separated from the mid-lobular and central vein (CV) zones. We found that the hematopoietic and cancer stem cell and surface marker SCA1 (encoded by the gene *Ly6a*; Upadhyay, 2019) labeled cells exclusively localized at the PT, in proximity to and including the biliary ductal epithelium, as identified by osteopontin (OPN). In contrast, SCA1 expression was absent or below detection limit in the remainder of the liver parenchyma (Figures 1A, 1B, S1A, and S1B). SCA1 expression was also detected in the CD31^+^ endothelium lining the portal vein, but not the VEGFR3^+^ sinusoidal endothelial network or liver-resident macrophages (F4/80^+^) (Figures S1A and S1B).

To determine whether SCA1^+^CD31^−^OPN^−^ cells encompassed a population of periportal mesenchyme, we analyzed the expression of SCA1 in livers derived from *Pdgfra-H2B-GFP* mice, which readily report expression of the archetypal mesenchymal marker PDGFRα (Figure S1C). We found that SCA1 labels a subpopulation of mesenchymal cells that surround and directly contact the ductal epithelium (Figures 1B–1D and S1D–S1G; Video S1) and express mesenchymal markers such as CD34, elastin, desmin, and reelin (Figures 1C, 1D, and S1D–S1F). PDGFRα^+^SCA1^+^ cells were located at a median distance of 8 μm from the center of the biliary duct, whereas this distance was more than tripled for the PDGFRα^+^SCA1^−^ fraction (Figure S1H). Accordingly, we utilized PDGFRα^+^SCA1^+^ expression as a proxy for identifying the mesenchymal cells nearest to the biliary epithelium and focused on this population from here onward. Pericytes (αSMA^+^) appeared to be distinct from the PDGFRα^+^SCA1^+^ mesenchyme (Figure S1G).

Video S1 Ductal cells are contacted by PDGFRα+SCA1+ mesenchyme in vivo, related to Figure 1 Video shows a representative Z stack of a liver section (8 μm) from a *Pdgfrα-H2B-GFP/mTmG* mouse stained for SCA1 (green), OPN (yellow) and Desmin (magenta). PDGFRα (magenta, nuclear) and membrane (white) are also shown, together with SiR-DNA nuclear staining (blue).5

**Figure 2:**
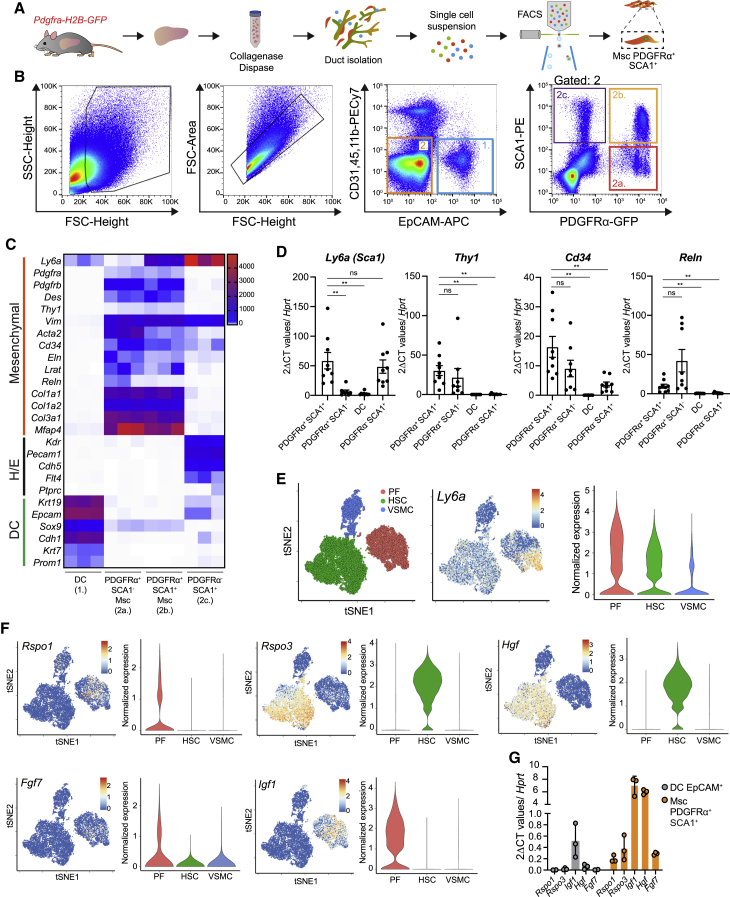
Periportal SCA1^+^Msc cells express a pro-regenerative growth factor signature (A and B) Isolation of EpCAM^+^ ductal cells (DCs; gate 1), PDGFRα^+^SCA1^−^ (gate 2a), and PDGFRα^+^SCA1^+^ (gate 2b) Msc and PDGFRα^−^SCA1^+^ (gate 2c) stromal cells from *Pdgfra-H2B-GFP* mouse livers. (A) Experimental design. (B) Representative FACS plots. (C) RNA sequencing (RNA-seq) analysis of the populations in (B). Heatmap represents the TPM (transcripts per million) values from the RNA-seq for the indicated genes (n = 3 biological replicates). H/E, hematopoietic/endothelial cell markers. (D) RT-qPCR expression analysis of selected genes from freshly sorted DCs and specified niche cells in (B). Graph represents mean ± SEM of n > 8 biological replicates (mice) from n = 3 independent experiments. Unpaired t test with Welsch correction (^∗∗^p < 0.01; ns, p > 0.01). (E and F) scRNA-seq analysis of mouse hepatic Msc populations reported in Dobie et al. (2019). tSNE [t-distributed stochastic neighbor embedding] (left) and violin plots (right) indicating the *mRNA* expression levels for SCA1 (*Ly6a*; E) or the indicated growth factors (F). PF, portal fibroblast; HSC, hepatic stellate cell; VSMC, vascular smooth muscle cell. (G) Gene expression analysis of selected secreted growth factor genes in the indicated sorted populations. Graph represents mean ± SD of n = 3 biological replicates (mice). See also Figure S2.

For an in-depth analysis of the peri-ductal associated stroma, we isolated PDGFRα^+^SCA1^+^ as well as PDGFRα^−^SCA1^+^, PDGFRα^+^SCA1^−^, and EpCAM^+^ DCs from healthy murine livers and obtained their transcriptional profile (Figures 2A and 2B). Transcriptome and qRT-PCR analysis confirmed that the PDGFRα^+^SCA1^+^ and PDGFRα^+^SCA1^−^ cells expressed a clear mesenchymal gene signature, including markers such as *Pdgfra*, *Pdgfrb*, *Eln*, and *Cd34* and various collagens (*Col1a1* and *Col1a2*). In contrast, endothelial (e.g., *Kdr*) genes were highly expressed in the PDGFRα^−^SCA1^+^ fraction, while ductal (e.g., *Krt19*)-specific genes were highly expressed in the EpCAM^+^ fraction, as expected. Endothelial and DC markers were weakly expressed or absent in the PDGFRα^+^SCA1^+^ cells (Figures 2C, 2D, S2A, and Data S1), while some mitogens such as *Rspo1* and *Hgf* were expressed (Figure S2B and Data S1). Moreover, Thy1 (Katsumata et al., 2017) and *Lrat* (Mederacke et al., 2013), purported PF and HSC markers, respectively, were also expressed in both PDGFRα^+^SCA1^+^ and SCA1^−^ populations (Figures 2C and 2D) while reelin (*Reln*), a well-known HSC marker, was mainly present in the PDGFRα^+^SCA1^−^ fraction (Figures 2C and 2D), in agreement with our immunostaining analysis (Figure S1E). These results thereby suggested a high degree of mesenchymal cell heterogeneity.

To gain deeper insight into this heterogeneity and increase the resolution of the SCA1^+^ mesenchymal cell expression profile, we utilized our published single-cell RNA sequencing (scRNA-seq) data of murine liver mesenchyme (Dobie et al., 2019), where three distinct mesenchymal cell clusters are readily identified (Figure 2E): an *Acta2*-enriched vascular smooth muscle cell (VSMC) population, an HSC cluster marked by *Lrat*- and *Reelin*-positive cells, and a PF cluster marked by *Cd34-*expressing cells (Figure S2C). High *Ly6a* (SCA1)^+^ cells were identified in the PF cluster, while cells expressing weaker levels were detectable within the HSC fraction (Figure 2E). Notably, both HSCs and PFs expressed various mitogens and growth factors, including *Rspo1*, *Rspo3*, *Fgf7*, and *Hgf* (Figures 2F and S2D), all essential for duct and hepatocyte specification (Rossi et al., 2001), regeneration (Hu et al., 2007; Kan et al., 2009; Yang et al., 2014), and organoid formation (Huch et al., 2013, 2015). These results were confirmed in sorted PDGFRα^+^SCA1^+^ cells (Figures 2G and S2B) as well as in PDGFRα^+^SCA1^+^ cells after sub-fractioning into PFs and HSCs using the bona-fide PF marker CD34 (PFs, CD34^+^; HSCs, CD34^−^) (Figures S2E and S2F).

Collectively, these results suggested that PDGFRα^+^SCA1^+^ mesenchymal cells (hereafter SCA1^+^Msc cells) represent a periportal, duct-contacting, mesenchymal subpopulation that expresses markers of both PFs and HSCs and is enriched in paracrine mitogens capable of modulating DC expansion.

### The cellular ratios and cell contacts between DC and SCA1^+^ mesenchyme change dynamically during the damage-repair response and negatively correlate with DC proliferation

*In vivo*, the damaged-induced proliferation of DCs is facultative and arrests once the tissue is regenerated, thus warranting the return to homeostasis (Cordero-Espinoza and Huch, 2018). Having observed that SCA1^+^Msc cells express mitogens known to regulate DC expansion during regeneration and organoid formation, we hypothesized that the relative abundance and contact between both populations could dictate the proliferative state of the ductal epithelium.

**Figure 3:**
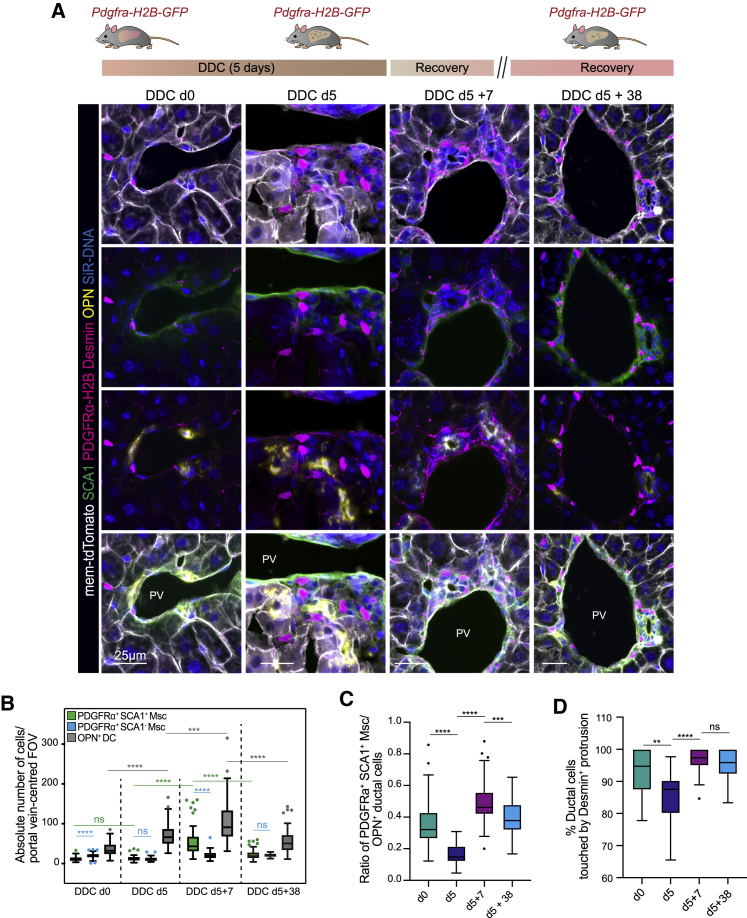
Relative abundance and cell contacts between DCs and PDGFRα^+^SCA1^+^Msc cells change dynamically during the damage-regenerative response (A–D) The number, distribution, and cell contacts between DCs, SCA1^+^Msc cells, and SCA1^−^ Msc cells were quantified before and after inducing liver injury and at days 7 and 38 of recovery. (A) Top: scheme of experimental approach. Bottom: representative maximum intensity projection composite images of livers from *Pdgfra-H2B-GFP/mTmG* mice stained for desmin (magenta), SCA1 (green), and OPN (yellow), *Pdgfra-H2B-GFP* (nuclear; magenta), membrane tdTomato (white), and nuclei (SiR-DNA, blue). (B) Box-and-whisker Tukey plot (median, whiskers are 1.5 interquartile range) of the absolute number of mesenchymal (PDGFRα^+^SCA1^+^ and PDGFRα^+^SCA1^−^) and ductal (OPN^+^) cells per field of view (FOV) of PV-centered composite confocal images from DDC-damaged livers at day 0 (n = 3), day 5 (n = 3), day 5 plus 7 days recovery (n = 3), and day 5 plus 38 days recovery (n = 2). Dots represent outliers. Mann-Whitney tests; ^∗∗∗∗^p < 0.0001; ^∗∗∗^p < 0.001; ns, p > 0.05. (C) Box-and-whisker Tukey plot (median, whiskers are 1.5 interquartile range) of the ratio of the number of PDGFRα^+^SCA1^+^Msc cells relative to DCs. Dots, outliers. Mann-Whitney t tests; ^∗∗∗∗^p < 0.0001; ^∗∗∗^p < 0.001. (D) Box-and-whisker Tukey plot (median, whiskers are 1.5 interquartile range) represents the percentage of DCs contacted by a desmin protrusion in DDC-damaged livers (n ≥ 3 mice). Dots, outliers. Mann-Whitney test; ^∗∗^p < 0.001; ^∗∗∗∗^p < 0.0001. See also Figure S3.

To test this hypothesis, we modeled acute liver damage by feeding mice with 0.1% 3,5-diethoxycarbonyl-1,4- dihydrocollidine (DDC) for 5 days, followed by a recovery period in normal diet for 7 and 38 days (Figures 3A, S3A, and S3B), and quantified total cell numbers, cell ratios, and cell contacts between DCs and SCA1^+^Msc (Figures 3B–3D). In healthy tissue, DCs display limited proliferation (Figure S3C). SCA1^+^Msc and DC (OPN^+^) co-exist periportally within close proximity (∼11 μm) (Figures 3A, S3A, S3B, and S3D), with a median population ratio of 0.3 Msc cells per 1 DC (0.3:1, from hereon) (Figure 3C) and the majority of DCs (∼93%) contacted by a SCA1^+^Msc cell (Figures 3A, 3D, and S3E). Following tissue damage (DDC day 5), DCs, but not PDGFRα^+^ (SCA1^+^ or SCA1^−^) cells, increase in number (Figures 3B and S3C). This results in a significant drop in the PDGFRα^+^SCA1^+^/DC cell ratio (from 0.3:1 to 0.1:1) (Figure 3C), increased distance between both compartments (from ∼11 μm to ∼30 μm), and a significant decrease in the number of cell contacts between both populations (from ∼93% to ∼84%) (Figures 3D and S3E). By the early phase of recovery ( DDC day 7, after recovery), the DC pool was still enlarged (Figure 3B), but the percentage of proliferating cells had diminished significantly to its pre-damage condition (Figure S3C). This coincided with an increase in the absolute number of SCA1^+^Msc cells (Figure 3B), a raise in the cell ratios from 0.1:1 to 0.5:1 (Figure 3C), a return to the baseline distance (Figures 3A, S3A, S3B, and S3D), and a reestablishment of the cell contacts (from ∼84% to 96%) (Figures 3A and 3D). At day 38 of recovery (termination phase), both DCs and SCA1^+^Msc cells returned to their steady-state numbers, ratios, spatial disposition, and cell contacts (Figures 3A–3D, S3A, S3B, and S3E). Of note, the PDGFRα^+^SCA1^−^ mesenchymal compartment did not increase its numbers during the entire damage-regenerative response (Figure 3B).

Taken together, these results indicate that both cell populations proliferate, but at different tempo; the DCs proliferate first, during damage (day 5), followed by the mesenchymal cells after the damage is removed (day 7, after recovery). This asynchrony results in a significant and dynamic change in the distance, cellular ratios, and number of cell contacts between both populations. Accordingly, we can hypothesize a scenario whereby in steady state, SCA1^+^Msc cells hold the ductal epithelium in a nonproliferative state; in contrast, upon damage, DC proliferation is prioritized, presumably by mitogenic signals, which results in a temporary drop in the steady-state ratios and cell contacts. Upon cessation of damage, mesenchymal cells expand and reestablish physical contacts with the ductal epithelium, which eventually reinstates the homeostatic, nonproliferative, steady state.

### Mesenchyme-secreted factors activate DC proliferation and organoid formation

To test the hypothesis that the relative abundance and cell contacts between the two populations could control the proliferative state of the DCs during regeneration, we studied how dynamic changes in Msc/DC numbers and cell contacts impact epithelial cell behavior. For that, we opted to manipulate the cellular ratios between both populations *in vitro*, in organoid co-cultures, where experimental conditions can be controlled.

Like the regenerating tissue, organoids also depend on key growth factors that mimic the mitogenic microenvironment of the damaged liver. Considering that the SCA1^+^Msc population expressed a battery of mitogens (Figures 2D–2G, S2B, and S2D) required for liver organoids to expand (Huch et al., 2013), we first sought to determine whether these cells would support the growth of liver ductal organoids *in vitro*. For that, we first identified culture conditions that would enable the maintenance of these mesenchymal cells *in vitro*. We selected AdDMEM/F12 supplemented with FBS and WNT3A (hereafter called mesenchymal medium [MM]) and culturing on plastic to enhance mesenchymal cell viability (Figures S4A and S4B). Given the low yield of primary PDGFRα^+^SCA1^+^ and PDGFRα^+^SCA1^−^ mesenchymal cells isolated from murine livers (Figure S4C), we investigated if our optimized culture conditions would enable expansion of these cells prior to co-culture. We readily expanded PDGFRα^+^SCA1^+^ cells (∼2 months in culture, passage 5) (Figures S4D–S4G), while the endothelial PDGFRα^−^SCA1^+^ cells could not be consistently grown (Figure S4H). Notably, sub-fractioning the PDGFRα^+^SCA1^+^ cells into HSCs and PFs using CD34 (PFs, CD34^+^ and HSCs, CD34^−^), indicated that the cells that expanded in culture were mainly PFs (CD34^+^) (Figure S4I). Similarly, from the PDGFRα^+^SCA1^−^ fraction, only PF (CD34^+^) cells could be expanded, but at much lower efficiency (Figure S4I). Expanded PDGFRα^+^SCA1^+^ cells conserved their morphology, secretome, and marker expression for the first three passages in culture (Figures S4D–S4G) but at passages 2–3 became activated (*αSma*^*+*^) (Rockey et al., 1992) and lost *Rspo1* expression (Figure S4F).

**Figure 4:**
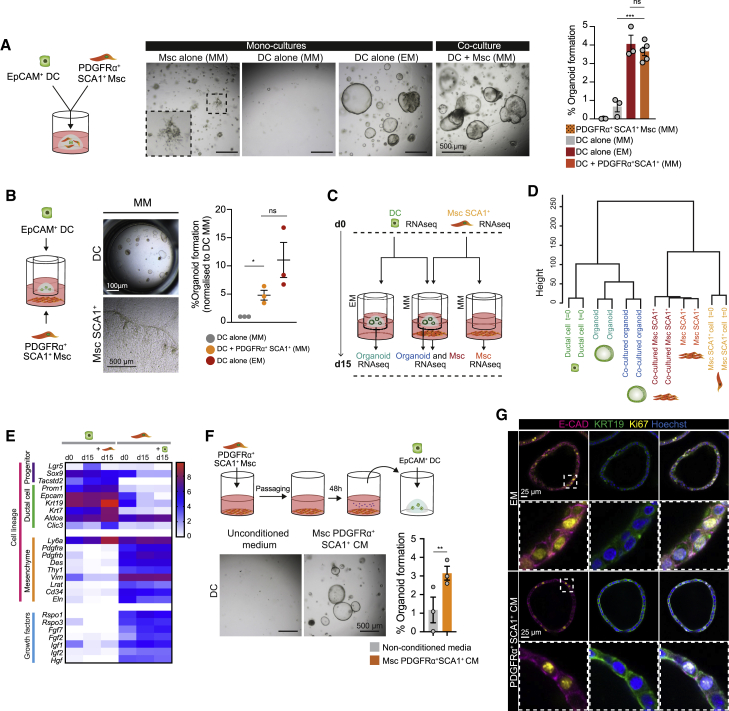
PDGFRα^+^SCA1^+^ mesenchymal cells support organoid formation via secreted growth factors (A) Organoid-formation efficiency of EpCAM^+^ DCs and PDGFRα^+^SCA1^+^Msc cells cultured either alone (monoculture) or together (co-culture, 3,600 DCs and 18,000 Msc) in mesenchymal medium (MM) or DCs cultured alone in complete expansion medium (EM). Left: experimental design. Middle: representative brightfield images. Right: graph representing mean ± SEM of the percentage of organoid formation at day 10 obtained from at least n = 3 independent biological replicates. Student’s t test; ^∗∗∗^p < 0.001; ns, p > 0.1. (B) Organoid-formation efficiency of freshly sorted EpCAM^+^ DCs seeded on a transwell insert alone in EM or MM or co-cultured for 10 days with freshly sorted PDGFRα^+^SCA1^+^ cells in MM. Left: schematic of a transwell co-culture. Middle: representative brightfield images of a transwell co-culture at day 10. Organoids, upper chamber. Msc, bottom chamber. Graph represents mean ± SEM of the percentage of organoid formation obtained from n = 3 independent biological replicates. Student’s t test; ^∗^p < 0.1; ns, p > 0.1. (C–E) RNA-seq analysis of DCs and SCA1^+^Msc sorted cells collected at day 0 (prior to culture), cultured alone or co-cultured in a transwell (day 15). (C) Experimental design. (D) Unsupervised clustering analysis of global mRNA expression in DCs and SCA1^+^Msc cells. (E) Heatmap representing the mean log_2_(TPM+1) value of the indicated genes from n = 2 independent biological replicates. (F) PDGFRα^+^SCA1^+^ conditioned medium (CM) or unconditioned MM was added to freshly sorted EpCAM^+^ cells, and organoid formation was assessed at day 10. Top: experimental design. Bottom: representative brightfield images. Graph represents the percent organoid formation efficiency at day 10. Results are shown as mean ± SEM of n = 3 independent experiments, each with two biological replicates. Unpaired t test with Welsch correction; ^∗∗^p < 0.01. (G) Immunofluorescence analysis of organoids derived from sorted EpCAM^+^ DCs cultured for 10 days in complete medium (EM) or Msc CM. Single composite z stack images of organoids stained for E-cadherin (magenta), KRT19 (green), proliferation (Ki67, yellow), and nuclei (blue). Representative images of n = 3 independent experiments. See also Figure S4.

Next, we co-isolated SCA1^+^Msc and EpCAM^+^ DCs and embedded them together inside of 3D Matrigel droplets that were overlaid with MM medium (devoid of growth factors). Interestingly, we observed that co-cultures with SCA1^+^Msc cells sustained organoid formation at an efficiency close to 4%, which was comparable to controls receiving the media supplemented with all growth factors and 4-fold higher than DC alone (Figure 4A). This effect was independent of WNT3A and FBS in the MM medium, since similar results were obtained when basal medium devoid of these components was used (Figure S4J). Culturing SCA1^+^Msc cells on their own did not generate organoids, as expected (Figure 4A, Msc alone panel). Notably, sub-fractioning for CD34^+^ PFs indicated that both SCA1^+^ and SCA1^−^ PFs were able to support organoid growth when expanded (Figure S4K), in contrast to control mouse embryonic fibroblasts (MEFs) (Figure S4L).

However, considering (1) the low yield of all the sub-fractioned populations, (2) the inability to expand the CD34^−^ cells in our medium, (3) that only the PDGFRα^+^SCA1^+^CD34^+^ fraction can be readily expanded further than P1, and (4) that the majority of the PDGFRα^+^SCA1^+^ cells are CD34^+^ (∼85% of all SCA1^+^ cells are CD34^+^), from here on, we only used PDGFRα^+^SCA1^+^ cells without sub-fractioning for CD34.

To decipher whether the organoid-supportive ability of the SCA1^+^Msc cells relied on close proximity or soluble growth factors, we co-cultured DC and PDGFRα^+^SCA1^+^ cells within transwell-fitting plates to prevent cell contact between both populations (Figures 4B–4E). Under these conditions, we observed a remarkably similar 4-fold increase in organoid formation efficiency in DC/SCA1^+^Msc co-cultures compared to DCs alone (Figure 4B), suggesting that the secreted growth factor repertoire of the SCA1^+^Msc cells was directly responsible for supporting DC proliferation and organoid formation. To determine the nature of the organoid structures formed upon co-culture, we compared their molecular identity to that of control organoids grown in growth-factor-rich medium. For that, we performed RNA sequencing of DCs immediately after sorting (day 0) and following 15 days of culture alone in expansion medium (EM; medium supplemented with growth factors) or in a transwell co-culture with SCA1^+^Msc cells in MM (medium devoid of growth factors) (Figures 4C–4E). Control organoids exposed to MM alone displayed minimal growth and could not be sequenced. The use of transwells enabled us to obtain the expression profile of each population independently before and after co-culture. Hierarchical clustering analysis revealed that organoids supported by SCA1^+^Msc cells closely resembled organoids cultured in EM (Figure 4D). They expressed progenitor (*Tacstd2*, *Sox9*, and *Lgr5*) as well as DC markers (*Krt19* and *Epcam*) (Figure 4E), suggesting that SCA1^+^Msc cells are capable of activating differentiated DCs to a proliferative state that enables organoid formation. Notably, the expression prolife, including lineage markers and secretome, of the SCA1^+^Msc remained relatively unaltered upon 15 days in culture, either alone or when co-cultured with DCs in a transwell (Figure 4E), in agreement with our characterization upon passages (Figures S4D–S4G) and arguing against a phenotypic transformation *in vitro*. Remarkably, conditioned medium from serially passaged mesenchymal cells supported organoid formation at a similar mean efficiency (3.3%) compared with non-expanded SCA1^+^Msc cells, suggesting that the cells remain functional even upon expansion (compare Figures 4F with Figures 4A and 4B). Immunofluorescence analysis indicated that the mesenchymal-supported organoids were formed by a single-layer epithelium of proliferative DCs (Krt19^+^, Ki67^+^), similar to organoids grown in complete medium (Figure 4G).

Collectively, these results highlighted that both freshly isolated and *in*-*vitro*-expanded SCA1^+^Msc cells secrete pro-mitogenic factors that enable the activation of differentiated DCs into self-renewing liver organoids *in vitro*.

### SCA1^+^ mesenchymal cells dually control DC behavior by promoting or arresting DC proliferation in a cell-contact-dependent manner

The capacity of the PDGFRα^+^SCA1^+^ mesenchyme to induce DC proliferation and organoid formation *in vitro* resembled the context of a regenerating liver yet was seemingly at odds with the low proliferative index of the ductal epithelium in homeostasis, from where both cell populations derived. This led us to reexamine the fidelity of our culturing methods and to redesign a culture system that would recapitulate physiological liver architecture. At the PT, SCA1^+^Msc cells are found in the immediate vicinity of DCs, physically wrapping the ductal epithelium (refer to Figures 1B–1D). Recapitulating this cell contact was crucial to characterize the cell interactions beyond paracrine signaling, yet such cell proximity became unavoidably disrupted following cell sorting, and our culturing methods using Matrigel droplets (Figure 4A), transwells (Figures 4B–4E), and conditioned media (Figure 4F) failed to reestablish it (Figure S4M).

**Figure 5:**
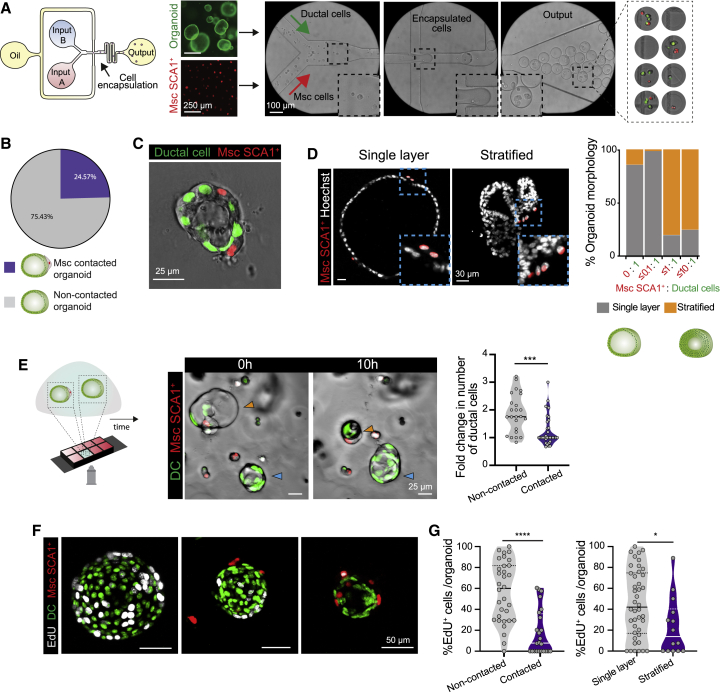
Mesenchymal-ductal organoids recapitulate the *in vivo* duct/Msc architecture of the portal tract (A) Microfluidic setup for cell encapsulation of DCs and Msc, with a flow-focusing device (FFD) containing two separate inlets for cell loading (input A and input B, in aqueous phase), one inlet for the continuous phase (oil) and one outlet. Representative brightfield images of encapsulated microgels are shown. (B) Frequency of formation of Msc-contacted organoids (containing DCs + SCA1^+^Msc) at day 4 following encapsulation. n = 4 independent experiments. (C) Representative composite single z stack image of a Msc-contacted organoid at day 4 post-encapsulation exhibiting a single-layer ductal (nuclear GFP^+^) epithelium surrounded by mesenchymal (nuclear tdTom^+^) cells on the periphery. See Figure S5F for additional examples. (D) Representative composite single z stack immunofluorescence images of Msc-contacted organoids exhibiting cystic/single-layer epithelium (left) and stratified epithelium (right). Msc, red; nuclei, white. Percentage of organoid morphologies observed according to the ratio of SCA1^+^Msc per DC. Data are presented as mean from n = 3 independent experiments. (E) Time-lapse imaging (24 h) of Msc-contacted (nuclear GFP^+^ and nuclear tdTom^+^) versus non-contacted (nuclear GFP^+^) organoids grown within the same Matrigel droplet and culture medium. Left: experimental design. Middle: stills of a time-lapse at day 4 after co-encapsulation. Non-contacted organoid grows (blue arrowhead), while the Msc-contacted organoid collapses (orange arrowhead). Violin plot indicates the data-point distribution, median, and interquartile range (IQR) of the fold change on number of DCs following 24 h of imaging in non-contacted versus Msc-contacted structures obtained from n = 3 experiments. Dot, independent organoid. Mann-Whitney test; ^∗∗∗^p < 0.001. (F) Representative composite maximum projected z stacks images of organoids immunostained for EdU (white) assessed in day 5 co-cultures following incubation with 10 μM EdU for 16 h. (G) Violin graphs represent the distribution, median, and IQR of the percentage of EdU^+^ DCs in non-contacted versus Msc-contacted organoids (left) and in single-layer/cystic versus stratified organoids (right) presented in (F) from n = 3 independent biological replicates. Mann-Whitney test; ^∗∗∗∗^p < 0.0001; ^∗^p < 0.1. See also Figure S5 and Videos S2, S3, and S4.

Aiming to reconstitute the ductal-to-mesenchymal cell architecture of the PT *in vitro*, we tested a microfluidics-based approach for co-encapsulating ductal and mesenchymal cells into microgel droplets (70 μm in diameter), such that by restricting the spatial surroundings of the two cell types, we would increase the probability of their physical aggregation. As an experimental setup, we utilized ductal organoids and *in vitro* expanded SCA1^+^Msc cells. The epithelial (GFP^+^) and mesenchymal (tdTom^+^) populations were resuspended in agarose and loaded separately onto custom-designed microfluidic flow-focusing devices (FFDs; Figures 5A, S5A, and S5B). As the encapsulation process follows a Poisson distribution, multiple permutations were observed, including separate encapsulation of both cell types and a large number of gels without cells (Figures 5A and S5C). Co-encapsulation (i.e., the presence of both cell types in one microgel) occurred in ∼6% of all events (Figure S5C).

Following encapsulation, the agarose microgels were embedded into Matrigel and cultured in MM for 4–5 days to allow organoid growth. No organoids were formed when seeding the microgels into agarose or in Matrigel, when encapsulation was performed in the absence of mesenchyme (Figures S5D–S5E). We detected the formation of multicellular complex organoids containing both ductal and mesenchymal cells that had established contact at an efficiency of ∼25% (Figure 5B). The layout of the Msc-ductal structures was reminiscent of the *in vivo* spatial arrangement, with the mesenchymal cells positioned on the basal surface of the biliary epithelium (compare Figures 5C and S5F with Figure 1B). We observed a preferential radial distribution (∼51%) of the mesenchyme around the ductal structure, as it occurs *in vivo* (Figures S5F–S5H, category b), but we also encountered unilateral segregation of multiple SCA1^+^Msc cells (∼28% of cases) (Figures S5F–S5H, category c). The organoids in contact with mesenchymal cells exhibited additional diversity in terms of their epithelial architecture. We found some complex organoids retaining the single-layer epithelial architecture, with cells encircling a central lumen, typical of ductal organoids, while others presented a pseudo-stratified epithelium (Figure 5D). We noted that these two types of architectural arrangements correlated with the proportions of Msc and DCs within the complex organoids, such that structures with an Msc/DC ratio of ≤0.1 retained their single-layer epithelial architecture, but in ratios of >0.1, a stratified epithelium developed (Figure 5D).

To probe the effect of mesenchymal cell contact on DC expansion, we tracked individual organoids in culture and performed time-lapse imaging from day 4–5 of seeding, when the process of organoid formation had commenced. Surprisingly, we found a very interesting dichotomous behavior. The majority of non-Msc-contacted organoids (GFP^+^ only) augmented in cell numbers and organoid area as time progressed (Figures 5E and S5I; Videos S2 and S3), as expected from being in the presence of mesenchyme-derived mitogens and reminiscent of our conditioned medium and transwell experiments (Figures 4B–4G). In stark contrast, mesenchyme-contacted organoids exhibited a significant paucity in growth (Figures 5E and S5I; Videos S2 and S4). This correlated with a reduced proliferative potential in the ductal compartment, assayed by EdU incorporation, which was exacerbated with higher doses of Msc contact (Figures 5F and 5G). Moreover, we noted a correlation between pseudo-stratified epithelial organization and decreased DC proliferation (Figure 5G). Mesenchymal cells forming part of multicellular complex organoids, on the other hand, rarely increased in numbers over the time assayed (Figure S5J).

Video S2 Non-contacted and mesenchyme-contacted chimeric organoids, related to Figure 5 Video shows mesenchymal contacted and non-contacted organoids. Mesenchymal (SCA1^+^) cells are shown in nuclear red while DCs are nuclear green. Note that, while the mesenchymal contacted organoid involutes and collapses, the non-contacted organoid keeps expanding through the course of the analysis. Stills from this video are provided in main Figure 3E.6

Video S3 Non-contacted organoid, related to Figure 5 Video shows a growing organoid (nuclear green) next to, but not contacted by, a mesenchymal (SCA1^+^) cell (nuclear red).7

Video S4 Mesenchyme-contacted organoid, related to Figure 5 Video shows an organoid (nuclear green) contacted by a mesenchymal cell (nuclear red). Note that on the course of the imaging the contacted organoid collapses and loses its initial structure.8

These observations suggested that SCA1^+^Msc cells regulate DC behavior in two ways: secreting pro-proliferative signals yet inducing growth arrest via cell contact.

### SCA1^+^Msc cells mediate DC proliferation arrest through Notch cell-cell contact inhibition

Having observed the paradoxical behavior of the SCA1^+^Msc population *in vitro*, we hypothesized that it is the number of contacts and ratios between the two populations which ultimately control the DC state.

**Figure 6:**
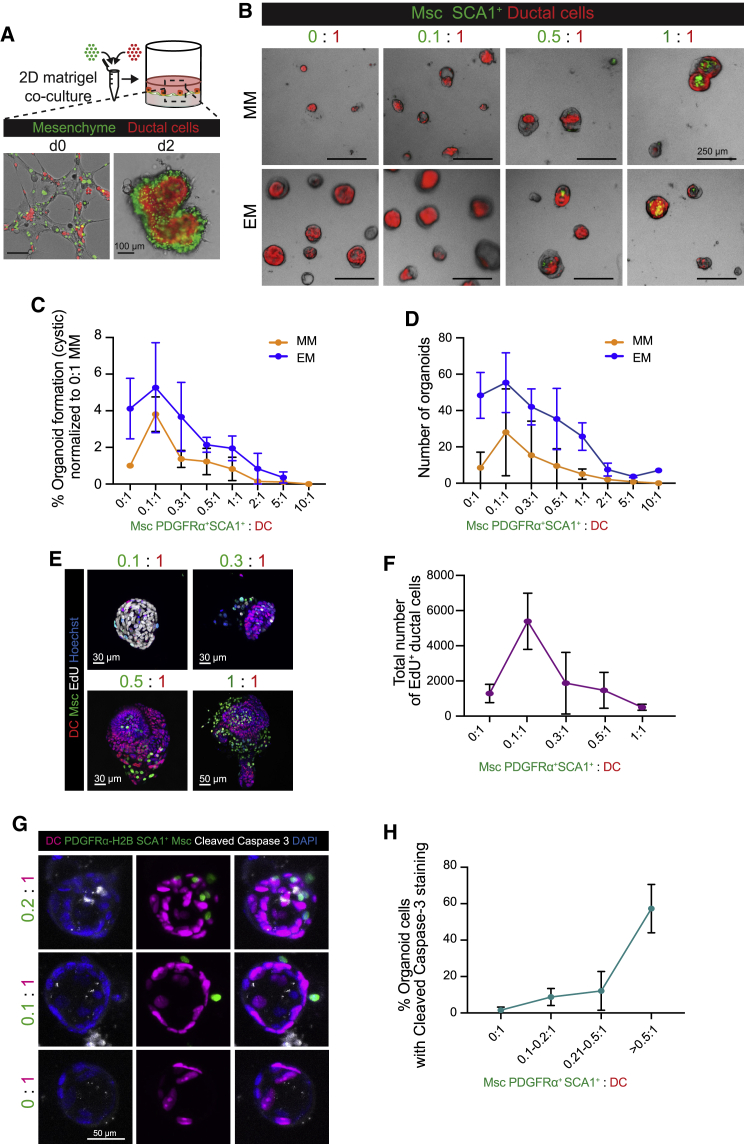
Dosage of cell contacts between PDGFRα^+^SCA1^+^Msc and DCs determines the net outcome of DC proliferation (A) DCs (nuclear tdTom^+^, red) were mixed at different ratios with PDGFRα^+^SCA1^+^Msc cells (nuclear GFP^+^, green) and seeded on top of a 2D layer of Matrigel. Representative composite image of a 1:1 ratio co-culture at days 0 and 2. (B and C) Freshly sorted DCs (red) were co-cultured with increasing ratios of SCA1^+^Msc cells in either growth-factor-devoid medium (MM) or growth-factor-rich medium (EM) for 8 days. (B) Representative composite images. (C) Quantification of cystic/single-layer organoid formation efficiency at the indicated ratios in MM (orange) or EM (blue) at day 8, normalized to the DCs alone in MM (ratio 0:1). Graphs denote mean ± SD of n = 3 (EM) and n = 4 (MM) independent experiments. (D) Total numbers of organoids from (C). (E and F) DC co-cultured with increasing numbers of SCA1^+^Msc cells were incubated with 10 μM EdU at day 6, and the number of proliferating cells was quantified 16 h later. (E) Representative composite images; EdU, white; nuclei, blue. (F) Graph representing the total number of EdU^+^ DCs in the co-cultures at the indicated ratios. Mean ± SD of n = 2 independent experiment with >22 organoids per condition. (G) Representative composite maximum intensity projection of Msc-contacted (nuclear green) and non-contacted organoids (nuclear magenta), stained for cleaved caspase-3 (white) at day 7. Nuclei, blue; the ratio of Msc to DC is specified for each structure. (H) Percentage of DCs stained with cleaved caspase-3 in non-contacted or Msc-contacted structures; graph shows mean ± SD of n = 3 independent experiments with bins specified by the ratio of Msc to DC in each organoid structure. See also Figure S6.

To test this, we opted to recapitulate the regeneration spectrum of PDGFRα^+^SCA1^+^/DC ratios *in vitro*. The microfluidics-based encapsulation method allowed this to a certain extent (Figure 5), but there was no exogenous control on the final output of the aggregated Msc/DCs, which hampered the systematic analysis of cell interactions at different ratios. We thus devised a contact-permissive co-culture method (Figures 6A and S6A), which generated multicellular organoids at an efficiency of 94.6% at a 1:1 ratio (Figure S6B), from which we inferred that the cell contact between both populations was directly proportional to the ratio seeded. This allowed the systematic study of cell interactions, both paracrine and cell bound, at the whole-population level instead of on an organoid-per-organoid basis as required with the microfluidics approach.

Using this co-culture method, we seeded increasing numbers of SCA1^+^Msc (GFP^+^) cells with a fixed number of sorted DCs (tdTom^+^) both in MM medium (devoid of growth factors) and in complete medium (EM) (Figures 6B–6D). In MM, the ratio of 0.1:1 (PDGFRα^**+**^SCA1^+^ cells/DCs) resulted in a 3.8-fold increase in organoid formation relative to DCs alone (Figures 6B–6D), resembling the organoid formation efficiency obtained when DCs were co-cultured in transwell or using condition medium (see Figures 4B and 4F). The DC expansion at 0.1:1 was gradually reversed as epithelial-mesenchymal contacts augmented until nearly abolishing organoid growth at ratios 1:1 and higher (Figures 6B–6D). Remarkably, this effect could not be compensated by a mitogen-rich microenvironment (Figures 6B–6D, EM), highlighting the strong cytostatic effect of the contacting mesenchyme. Moreover, at ratios higher than 0.1:1 (Msc/DCs), we observed a negative correlation between mesenchymal cell dosage and the total number of dividing DCs, while the number of apoptotic DCs significantly increased (Figures 6E–6H, S6C, and S6D). Importantly, this phenotype was indeed reliant on physical contact between the two cell types, given that transwell co-cultures at a 5:1 ratio robustly promoted, instead of inhibited, organoid expansion (compare Figure S6E with Figures 6C and 6D). Interestingly, *in vitro* lineage tracing of *Lgr5*^+^ progenitors –which are activated from differentiated DCs upon organoid culture – revealed a decreased percentage of proliferating *Lgr5*^*+*^ cells in organoids contacted by the mesenchyme, even in the presence of complete medium containing RSPO1 and supplemented with WNT3a (EM + WNT) (Figures S6F and S6G).

Collectively, these results suggest a potential mechanism whereby it is the relative abundance of contacts between DCs and their mesenchymal niche cells what curtails the size of the ductal pool. Taking into account the strong correlation between the *in vivo* and *in vitro* results (see Figure 3 versus Figure 6) regarding the number of cellular ratios, cell contacts, and ductal proliferation, our co-culture results suggest a potential scenario whereby *in vivo*, during the damage-regenerative response, the Msc population could act as a direct upstream regulator of the ductal proliferative state.

**Figure 7:**
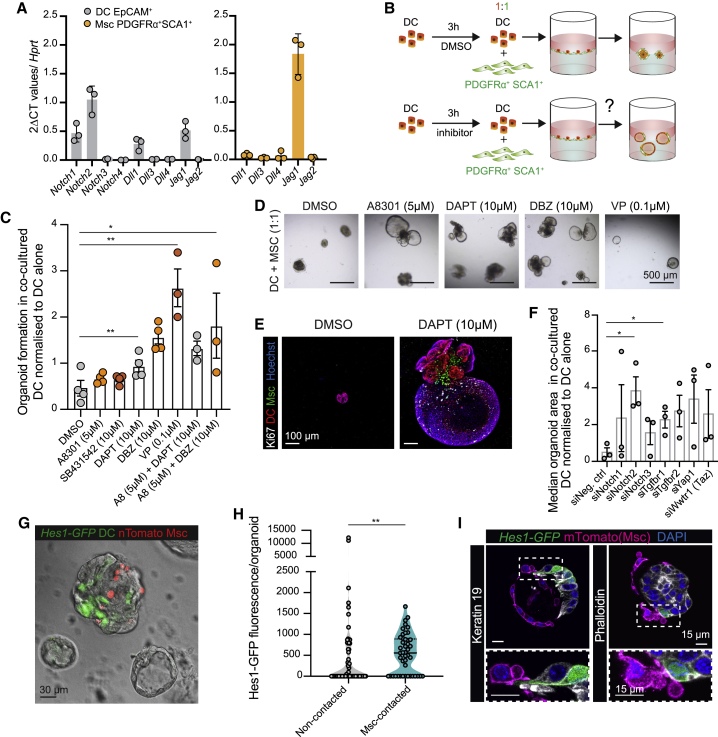
Cell contact from PDGFRα^+^SCA1^+^Msc cells inhibits DC proliferation via Notch signaling (A) RT-qPCR gene expression analysis on selected genes of the Notch pathway in freshly sorted EpCAM^+^ DCs (gray bars) and PDGFRα^+^SCA1^+^Msc cells (orange bars). Graphs represent mean ± SD of n = 3 independent experiments. (B–E) Freshly sorted EpCAM^+^ DCs (5,000 cells) were pretreated for 3 h with DMSO or the indicated inhibitors prior to being co-cultured at 1:1 ratio with 5,000 SCA1^+^Msc cells in MM. (B) Experimental design. (C) Graph represents cystic/single-layer organoid formation at day 10 in the DC/SCA1^+^Msc co-cultures normalized to that of the respective DC monocultures. Graphs display mean ± SEM from n ≥ 3 independent experiments. Student’s t test (all treatments compared to the DMSO). ^∗∗^p < 0.01; ^∗^p < 0.1; ns, p > 0.1. (D) Representative bright-field images from day 10 co-cultures. (E) Maximum projected composite images of chimeric organoids (DC, red; Msc, green) immunostained for Ki67 (white) and nuclei (blue) at day 10 after DAPT treatment. (F) Freshly sorted DCs (5,000 cells) were transfected with the indicated small interfering RNA (siRNA) and cultured alone or with 2,500 SCA1^+^Msc cells in MM at a 0.5:1 ratio (Msc/DCs). Organoid formation was assessed at day 10. Bar graph represents mean ± SEM of median organoid area normalized to the respective DC monocultures from n = 3 independent experiments. Student’s t test (compared to control); ^∗^p < 0.1; ns, p > 0.1. (G–I) Co-cultures between DCs sorted from *Hes1-GFP* mouse livers (green) and SCA1^+^Msc cells (ntdTom^+^, red) seeded at a 0.5:1 (Msc/DCs). The number of Hes1-GFP^+^ cells was assessed at day 8. (G) Representative bright-field and composite fluorescence image showing a contacted organoid (gray and red) with active Hes1-GFP (green) and non-contacted, Hes1-GFP^−^ organoids (gray). (H) Graph represents the Hes1-GFP mean fluorescence intensity and area per z stack, normalized to total area, in non-contacted versus Msc-contacted organoids. Data are presented as violin plots showing data-point distribution, median, and IQR of n = 2 independent experiments (n = 46 Msc-contacted and 68 non-contacted organoids). Mann-Whitney test; ^∗∗^p < 0.01. (I) Single z stack composite images of membrane tdTomato^+^ SCA1^+^Msc cells (magenta) establishing cell-cell contact with Hes1-GFP DCs. DC membranes were immunostained with Keratin-19 (white, left) or Phalloidin (white, right) and nuclei (blue). See also Figure S7.

To investigate the molecular basis for this contact inhibition, we examined our RNA-seq data in quest for proximity-based or juxtacrine signaling pathways wherein receptor(s) and ligand(s) could be paired between the two cell populations. We found that DC expressed *Notch1* and *Notch2*, *Tgfbr1* and *Tgfbr2* and the Hippo pathway downstream effectors *Yap1* and *Wwtr1*(TAZ), while SCA1^+^Msc expressed Notch and Tgfb ligands (Figures 7A and S7A; Data S1). Then, we devised a small-scale screening assay using small-molecule inhibitors of these pathways where sorted DC were pre-treated with vehicle or the indicated inhibitor(s) prior to being co-cultured with SCA1^+^Msc (Figure 7B). Pre-treated DCs were cultured alone or in the presence of SCA1^+^Msc cells in a ratio expected to suppress organoid growth, and results were normalized to DC alone, to account for non-mesenchymal derived phenotypes (Figure 7C). Compared to controls, pre-treatment with the gamma secretase inhibitors DAPT and DBZ and the YAP inhibitor verteporfin (VP) yielded a significant increase in organoid formation, while TGFβ inhibitors had no significant effect (Figures 7C, 7D, and S7B). Interestingly, the combination between Notch and TGFβ inhibitors (DAPT or DBZ + A8301) also rescued organoid growth (Figure 7C). Notably, organoids arising from DAPT pre-treated DC (labeled by tdTom^+^) were proliferative while still retaining physical interactions with the SCA1^+^Msc cells (Figure 7E).

To identify the potential effectors/receptors that regulate the mesenchymal cell contact inhibition, we next performed a small-scale siRNA knockdown of some components of the pathways in DCs during co-culture with Msc. Notably, *Notch2*, but not *Notch1* knockdown significantly increased DC expansion (Figures 7F and S7C), while *Notch3* (Figures 7A and S7A), showed no effect, as expected for not being expressed.

Given that Notch signaling is a well-known inducer of mature DC fate in hepatoblasts (Hofmann et al., 2010; Sparks et al., 2010; Zong et al., 2009) and adult hepatocytes (Jeliazkova et al., 2013), we decided to focus on this pathway as one of multiple potential mechanisms through which the mesenchyme modulates DC behavior in adulthood. To visualize Notch signaling in DCs upon Msc contact, we used *Hes1-GFP* mice (Klinck et al., 2011). In homeostatic livers, Hes1 expression was heterogeneous among DCs, even when all DCs were physically wrapped by SCA1^+^Msc (Figure S7D). DCs sorted from *Hes1-GFP* mice and cultured in complete medium generated organoids with limited GFP fluorescence (Figure S7E, 1), while isolated SCA1^+^Msc cells had undetectable *Hes1* expression (Figure S7E, 2). Co-culturing *Hes1-GFP* organoid cells with nuclear tdTom^+^SCA1^+^Msc cells at 1:1 ratio led to a higher percentage of *Hes1*-GFP DC relative to DC monocultures (Figure S7F). This was specific to co-cultures where cell contacts had been established, since Notch activation was not recapitulated upon addition of mesenchymal conditioned medium to DCs (Figure S7G). To better assess if contact was required for Notch signaling, we co-cultured *Hes1-GFP* ductal organoids with nuclear tdTom^+^SCA1^+^Msc cells at a 0.5:1 ratio so as to generate a mix of Msc-contacted and non-contacted organoid structures within the same well. Under these conditions, we found increased *Hes1-GFP* fluorescence in Msc-contacted structures (Figures 7G, 7H, and S7H). Notably, by using SCA1^+^Msc cells expressing a membrane-anchored tdTomato, we confirmed that mesenchymal-to-epithelial membrane contact activates Hes1-GFP expression in DCs (Figure 7I), albeit not in all cases (reminiscent of native tissues, Figure S7D), likely due to this being a snapshot of an otherwise dynamic process. In addition, we found that activation of Notch signaling significantly diminished DC proliferation (Figure S7I).

Altogether, these results suggest a mechanism by which, at least *in vitro*, SCA1^+^Msc cells induce DC proliferation arrest, in part by the juxtacrine activation of Notch signaling in DCs.

## Discussion

The regenerative capacity of the liver epithelium bespeaks not only cell-intrinsic plasticity but also an instructive microenvironment capable of guiding epithelial fate choices (Boulter et al., 2013). Previous work had reported on a hepatic population of SCA1^+^ cells residing at the PT, which expanded in damaged livers (Clayton and Forbes, 2009) and contributed to fibrosis (Katsumata et al., 2017). Here, we identify PDGFRα^+^SCA1^+^ cells as a periportal mesenchymal subpopulation whose stoichiometry with respect to the ductal compartment, dynamic in regeneration, dictates its behavior as a pro-proliferative or a cytostatic niche. We demonstrate a very interesting paradox behind the relationship between mesenchymal and neighboring ductal populations. While PDGFRα^+^SCA1^+^ mesenchymal-secreted factors induce DC proliferation, cell contact is cytostatic. This growth-inhibitory effect overrides mitogenic signals, as it cannot be rescued by supplementation of a mitogen-rich medium. Our results might reconcile the apparent dichotomy behind a regenerative, pro-proliferative cellular environment and a pro-quiescent, post-mitotic homeostatic cellular environment. We suggest a new concept whereby it is the number of cell-cell contacts with the mesenchymal niche that determines the outcome (proliferation or cellular arrest) of DC behavior. While we formally demonstrate this paradox *in vitro*, using organoid co-cultures, future studies will aim at elucidating this mechanism *in vivo*.

It is well established that juxtacrine or contact-dependent signaling like that of Notch occurs either between adjacent cells or proximate neighbors aided by filopodia extensions (Cohen et al., 2010; De Joussineau et al., 2003); in contrast, secreted ligands such as FGF (Christen and Slack, 1999) and HGF (Patel et al., 2015) are diffusible and span a signaling range of multiple cell diameters (Perrimon et al., 2012). The integration of paracrine and juxtacrine signals antagonistic to each other can potentially explain part of the population dynamics between DC and SCA1^+^Msc cells as follows: a low mesenchymal-to-DC ratio (0.1:1) maximizes DC proliferation via soluble factors while limiting mesenchymal contact to a few DCs; higher ratios, on the other hand, engage more DCs via juxtacrine signaling and eventually abolish DC proliferation. This is reminiscent of the concept of stem cell niche occupancy, whereby restrictions of niche factors, including abundance and signaling range, cause cells to compete with one another and regulate population asymmetry (Klein and Simons, 2011; Stine and Matunis, 2013). We showed an asynchrony in the DC and Msc expansion upon damage, with the DC expansion preceding that of SCA1^+^Msc cells, thereby reducing the ratio and the number of cell contacts. An outstanding question is the stimulus that first signals for DC proliferation and spreading. Future in-depth studies will aim at addressing this question.

The ductal-mesenchymal organoids developed here were instrumental for deciphering the population dynamics and molecular crosstalk between both populations but also hinted at the possibility of modeling adult liver histoarchitecture in a dish. Complex liver buds have been previously generated using pluripotent stem cell [PSC]-derived epithelium and mesenchymal populations (Ouchi et al., 2019; Takebe et al., 2013), but not with primary adult liver populations. In our study, DC and SCA1^+^Msc displayed cohesiveness by spontaneously aggregating with each other, a mechanism that could relate to mesenchymal-induced cell condensation (Takebe et al., 2015), yet promptly segregated into their respective compartments to recapitulate the spatial arrangement of homeostatic biliary ducts *in vivo*, where PDGFRα^+^SCA1^+^ cells wrap around but do not intermingle with the ductal epithelium. The principles governing this were beyond the scope of this work but are subject of great interest in understanding the self-organization of multicellular tissues (Takeichi, 2011).

In summary, our findings expand the concept of cellular niche in that it is the relative abundance of cell contacts, and not the absolute number of cells, that dictates the final outcome of epithelial proliferation during the different phases of the damage-regenerative response. Interestingly, in mouse prostate and muscle, mesenchymal SCA1^+^ populations have also been found to modulate epithelial proliferation and myogenic differentiation, respectively (Joe et al., 2010; Wei et al., 2019). It would be of great interest to study whether our observations translate to human tissue once a direct homolog of *Sca-1/Ly6a* in humans is identified (Upadhyay, 2019). While our studies have focused on liver DC interactions in the PT area, we envision similar mechanisms at play in any other system where cell numbers dynamically change as a consequence of external cues, such as the lung or breast epithelium.

### Limitations of the study

We focused on a subpopulation of mesenchymal PDGFRα^+^SCA1^+^ cells. One limitation, though, is our inability to expand SCA1^−^ fractions, which prevented us from testing whether the paradoxical behavior observed is a general feature of all or only a subset of liver mesenchymal cells. Another limitation is the low efficiency of co-encapsulation in our microfluidics system. Methods combining flow-based microfluidics could be leveraged to increase the yield of structures with the appropriate cellular ratios (Li et al., 2013). Also, while *in vitro* Notch signaling explains part of the cell contact inhibition mechanism, the plethora of behaviors observed upon direct mesenchymal-to-epithelial contact, including cell apoptosis and organoid collapse, cannot depend on a sole mechanism. It is tempting to speculate that it is the coalescence of several mechanisms, including biochemical signals and mechanical forces exerted by the Msc in the ductal epithelium, that explains the full phenotype. Accurately detangling one from the others will be a demanding yet not totally unsurmountable challenge to pursue in future studies.

## STAR★Methods

### Key resources table

**Table:** 

REAGENT or RESOURCE	SOURCE	IDENTIFIER
**Antibodies**		

Rat anti-Ly-6A/E (Sca-1) monoclonal (Clone D7), FITC	ThermoFisher Scientific	Cat# 11-5981-81; RRID: AB_465332
Rat anti-Ly-6A/E (Sca-1) monoclonal (Clone D7), PE	ThermoFisher Scientific	Cat# 12-5981-82; RRID: AB_466086
Rat anti-Ly-6A/E (Sca-1) monoclonal (Clone D7), Super Bright 436	ThermoFisher Scientific	Cat# 62-5981-82; RRID: AB_2637287
Rat anti-Ly-6A/E (Sca-1) monoclonal (Clone D7)	ThermoFisher Scientific	Cat# 14-5981-85; RRID: AB_467779
Rat anti- CD326 (EpCAM) monoclonal (Clone G8.8), APC	ThermoFisher Scientific	Cat# 17-5791-80; RRID: AB_2734965
Rat anti-CD31 monoclonal (Clone 390), PE-Cy7	Abcam	Cat# ab46733; RRID: AB_868905
Rat anti-CD31 monoclonal (Clone 390), PE-Cy7	BD Biosciences	Cat# 561410; RRID: AB_10612003
Rabbit anti-CD31 polyclonal	Abcam	Cat# ab28364; RRID: AB_726362
Rat anti-CD45 monoclonal (Clone 30-F11), PE-Cy7	BD Biosciences	Cat# 552848; RRID: AB_394489
Rat anti-CD45 monoclonal (Clone 30-F11), APC	ThermoFisher Scientific	Cat# 17-0451-83; RRID: AB_469393
Rat anti-CD11b monoclonal (Clone M1/70), PE-Cy7	BD Biosciences	Cat# 552850; RRID: AB_394491
Mouse anti-VEGF R3/Flt-4 Affinity Purified polyclonal	R&D Systems	Cat# AF743; RRID: AB_355563
Goat anti-Osteopontin Polyclonal	R&D Systems	Cat# AF808; RRID: AB_2194992
Rabbit anti-Cytokeratin, wide spectrum screening polyclonal	Agilent	Cat# Z0622; RRID: AB_2650434
Rat anti-Cytokeratin 19 monoclonal (Clone TROMA-III)	DSHB	Cat# TROMA-III; RRID: AB_2133570
Rat anti-F4/80 monoclonal (Clone CI:A3-1)	Abcam	Cat# ab6640; RRID: AB_1140040
Rabbit anti-Desmin polyclonal	Abcam	Cat# ab8592; RRID: AB_306653
Rabbit anti-Desmin polyclonal	Abcam	Cat# ab15200; RRID: AB_301744
Rabbit anti-Vimentin monoclonal (Clone EPR3776)	Abcam	Cat# ab92547; RRID: AB_10562134
Rabbit anti-alpha smooth muscle actin polyclonal	Abcam	Cat# ab5694; RRID: AB_2223021
Rabbit anti-CD34 monoclonal (Clone EP373Y)	Abcam	Cat# ab81289; RRID: AB_1640331
Rat anti-CD34 monoclonal (Clone RAM34), eFluor 660	ThermoFisher Scientific	Cat# 50-0341-82; RRID: AB_10596826
Goat anti-PDGFR- alpha polyclonal	R&D Systems	Cat# AF1062; RRID: AB_2236897
Goat anti-Reelin Affinity Purified polyclonal	R&D Systems	Cat# AF3820; RRID: AB_2253745
Rabbit anti-Ki67 (Ki-67) monoclonal (Clone SP6)	ThermoFisher Scientific	Cat# RM-9106-S1; RRID: AB_149792
Rabbit anti-Elastin polyclonal	CEDARLANE	Cat# CL55041AP; RRID: AB_10061195
Mouse anti-E-Cadherin monoclonal (Clone 34/E)	BD Biosciences	Cat# 610405; RRID: AB_397787
Mouse anti-Beta-Catenin monoclonal (Clone 14)	BD Biosciences	Cat# 610153; RRID: AB_397554
Rabbit anti-Phospho-Histone H3 (Ser10) polyclonal	Cell Signaling Technology	Cat# 9701; RRID: AB_331535
Rabbit anti-Cleaved Caspase-3 (Asp175) monoclonal (Clone 5A1E)	Cell Signaling Technology	Cat# 9664; RRID: AB_2070042

**Chemicals, peptides, and recombinant proteins**		

Collagenase from *Clostridium histolyticum*	Merck/Sigma	Cat# C9407
Dispase II	ThermoFisher Scientific	Cat# 17105-041
Fetal Bovine Serum	Merck/Sigma	Cat# F7524
Advanced DMEM/F-12	ThermoFisher Scientific	Cat# 12634010
DMEM, high glucose, GlutaMAX Supplement, pyruvate	ThermoFisher Scientific	Cat# 31966021
HEPES (1M)	ThermoFisher Scientific	Cat# 15630056
Penicillin/Streptomycin	ThermoFisher Scientific	Cat# 15140-122
GlutaMAX supplement	ThermoFisher Scientific	Cat# 35050-068
TrypLE Express Enzyme (1X), phenol red	ThermoFisher Scientific	Cat# 12605010
TrypLE Select Enzyme (10X), no phenol red	ThermoFisher Scientific	Cat# A1217701
B27-Supplement, serum free	ThermoFisher Scientific	Cat# 17504-044
N-2 Supplement	ThermoFisher Scientific	Cat# 17502-048
N-acetylcysteine (NAC)	Merck/Sigma	Cat# A9165
[Leu15]-Gastrin I Human	Merck/Sigma	Cat# G9145; CAS: 39024-57-2
Mouse EGF Recombinant Protein	ThermoFisher Scientific	Cat# PMG8041
Recombinant Human FGF-10	Peprotech	Cat# 100-26
Nicotinamide	Merck/Sigma	Cat# N0636
Rspondin 1 conditioned medium	Home made	Broutier et al., 2016 Nature Protocols
WNT3a conditioned medium	Home made	Broutier et al., 2016 Nature protocols
Recombinant Human HGF (Insect derived)	Peprotech	Cat# 100-39
Recombinant Human Noggin	Peprotech	Cat# 120-10C
Lipofectamine RNAiMAX	ThermoFisher Scientific	Cat# 13778030
ROCK inhibitor - Y-27632 dihydrochloride	Merck/Sigma	Cat# Y0503; CAS: 129830-38-2
Matrigel Growth Factor Reduced (GFR) Basement Membrane Matrix, Phenol Red-free	Corning	Cat# 356231
0.1% DDC (3,5-diethoxycarbonyl-1,4- dihydrocollidine) mouse diet	Custom Animal Diets, LLC	Cat# AD5001
Cell Recovery Solution	Corning	Cat# 354253
HFE-7500 3M (TM) Novec (TM) Engineered fluid	Fluorochem	Cat# 051243; CAS: 297730-93-9
Pico-Surf (5% (w/w) in Novec 7500)	Sphere Fluidics	Cat# C022
SeaPrep Agarose	Lonza	Cat# 50302
1H,1H,2H,2H-Perfluoro-1-octanol (PFO)	Merck/Sigma	Cat# 370533; CAS: 647-42-7
O.C.T. compound	VWR Chemicals	Cat# 361603E
Triton X-100	Merck/Sigma	Cat# T8787; CAS: 9002-93-1
Bovine serum albumin (BSA)	Merck/Sigma	Cat# A8806; CAS: 9048-46-8
Donkey serum	Merck/Sigma	Cat# D9663
Dimethyl sulfoxide (DMSO)	Merck/Sigma	Cat# D8418; CAS: 67-68-5
A8301 (TGF-βRI, ALK4 and ALK7 inhibitor)	TOCRIS	Cat# 2939/10; CAS: 909910-43-6
SB431542 (TGF-βRI, ALK4 and ALK7 inhibitor)	TOCRIS	Cat# 1614; CAS: 301836-41-9
DAPT (2S)-N-[(3,5-Difluorophenyl)acetyl]-L-alanyl-2-phenyl]glycine 1,1-dimethylethyl ester) (γ-secretase inhibitor)	Merck/Sigma	Cat# D5942; CAS: 208255-80-5
DBZ (Dibenzazepine) (γ-secretase inhibitor)	Merck/Sigma	Cat# D5942; CAS: 208255-80-5
Verteporfin	Merck/Sigma	Cat# SML0534; CAS: 129497-78-5
Vectashield	Vector Laboratories	Cat# H-1000-10
(Z)-4-Hydroxytamoxifen	Merck/Sigma	Cat# H7904-5MG; CAS: 68047-06-3
Hoechst 33342, Trihydrochloride, Trihydrate	ThermoFisher Scientific	Cat# H3570; CAS: 23491-52-3
SiR DNA	SpiroChrome	Cat# CY-SC007
DAPI	BD Biosciences	Cat# BD564907
Alexa Fluor Phalloidin 647	ThermoFisher Scientific	Cat# A22287
(Z)-4-Hydroxytamoxifen	Merck/Sigma	Cat# H7904-5MG; CAS: 68047-06-3

**Critical commercial assays**		

Click-iT EdU Alexa Fluor 594 Imaging Kit	ThermoFisher Scientific	Cat# C10339
Viability/Cytotoxicity Assay Kit for Animal Live & Dead Cells	Biotium	Cat# 30002
PicoPure RNA Isolation Kit	ThermoFisher Scientific	Cat# KIT0204
iTaq Universal SYBR Green Supermix	Bio-Rad	Cat# 172-5124
FastStart Essential DNA Green Master	Roche	Cat# 06402712001

**Deposited data**		

RNA sequencing data of sorted cells and co-cultures	GEO	GEO: GSE140697
scRNA sequencing of mesenchymal populations	Dobie et al., 2019	N/A

**Experimental models: Cell lines**		

Mouse: liver mesenchyme SCA1^+^	This paper	N/A
Mouse: liver mesenchyme SCA1^+^ PDGFRα^+^	This paper	N/A
Mouse: liver mesenchyme SCA1^+^ PDGFRα^+^ CD34^+^	This paper	N/A
Mouse: liver mesenchyme SCA1^-^ PDGFRα^+^ CD34^+^	This paper	N/A
Mouse: liver mesenchyme SCA1^+^ PDGFRα^+^ CD34^-^	This paper	N/A
Mouse: liver mesenchyme SCA1^-^ PDGFRα^+^ CD34^-^	This paper	N/A
Mouse: mouse embryonic fibroblasts (MEFs)	Gift from Ronald Naumann, MPI-CBG	N/A

**Experimental models: organisms/strains**		

Mouse: *Rosa26-mTmG* [Gt(ROSA)26Sortm4(ACTB-tdTomato,-EGFP)Luo/J]	The Jackson Laboratory	RRID: IMSR_JAX:007576
Mouse: *Rosa26-nTnG* [Gt(ROSA)26Sortm1(CAG-tdTomato^∗^,-EGFP^∗^)Ees]	The Jackson Laboratory	RRID: IMSR_JAX:023035
Mouse: Lgr5iresCreERT	Clevers Lab	Huch et al., 2013
Mouse: R26RtdTomato [Gt(ROSA)26Sortm9(CAG tdTomato)Hze/J]	The Jackson Laboratory	RRID: IMSR_JAX:007909
Mouse: Hes1-GFP [Tg(Hes1-EGFP)1Hri]	Serup Lab;	Klinck et al., 2011 Gene Expression Patterns
Mouse: *Pdgfra-H2B-GFP* [B6.129S4-Pdgfratm11(EGFP)Sor/J]	Zernicka-Goetz Lab	Hamilton et al., 2003; RRID: IMSR_JAX:007669

**Oligonucleotides**		

*See* Table S3 *for qRT-PCR primers*	N/A	N/A
*See* Table S4 *for siRNA oligos*	Dharmacon	N/A

**Software and algorithms**		

ZEN	Zeiss	https://www.zeiss.com/microscopy/int/products/microscope-software/zen-lite.html
ImageJ/Fiji	Schneider et al., 2012	https://imagej.nih.gov/ij/
FlowJo	FlowJo	https://www.flowjo.com/
Huygens	Scientific Volume Imaging	https://svi.nl/
Noise to Void	Github	https://github.com/juglab/n2v
Liver Cell Distances	This paper	https://github.com/gurdon-institute/Liver-Cell-Distances
Chimeric Organoid Analyzer	This paper	https://github.com/gurdon-institute/Chimeric-Organoid-Analyser
Prism9	GraphPad	https://www.graphpad.com/scientific-software/prism/

### Resource availability

#### Lead contact

Further information and requests for resources and reagents should be directed to and will be fulfilled by the lead contact, Meritxell Huch (huch@mpi-cbg.de).

#### Materials availability

This study did not generate new unique reagents.

### Experimental model and subject details

#### Cell culture

Organoids were cultured in AdDMEM/F12 (ThermoFisher, 12634010) medium containing HEPES (ThermoFisher, #15630-056), Penicillin/Streptomycin (ThermoFisher, #15140-122), Glutamax (ThermoFisher, #35050-068), 1% B27 (Invitrogen, #17504-044), 1% N2 (ThermoFisher, #17502-048) and 1.25mM N-acetylcysteine (Merck/Sigma, #A9165) –referred to as Basal medium–, which was further supplemented with 10nM gastrin (Merck/Sigma, #G9145), 50ng/ml mEGF (ThermoFisher, #PMG8043), 5% RSPO1 conditioned medium (homemade), 100ng/ml FGF10 (Peprotech, #100-26), 10mM nicotinamide (Merck/Sigma, #N0636) and 50ng/ml HGF (Peprotech, #100-39) –referred to as expansion medium (EM). Following isolation, EpCAM+ DC were embedded in Matrigel and cultured in EM supplemented with 30% WNT3a conditioned medium (WNT CM) (homemade), 25ng/ml Noggin (Peprotech, #120-10C) and 10 μM ROCK inhibitor (Ri) (Y-27632, Merck/Sigma, #Y0503) for 3 days and then were switched to standard EM. Organoids were passaged at a 1:3 ratio once a week or when fully grown through mechanical dissociation and re-embedded in fresh Matrigel and cultured in EM.

Mesenchymal cells were cultured in Basal medium supplemented with WNT CM (30%) referred to as mesenchymal medium (MM). Cells were passaged at 1:3 and 1:2 ratios, through enzymatic digestion using TrypLE Express (ThermoFisher, #12605010) for 5 min at 37°C. Ri was added to the MM when cells were seeded right after sorting or following passage. Mouse embryonic fibroblasts (MEFs) were cultured in DMEM (ThermoFisher, #31966021) supplemented with 10% FBS (Merck/Sigma, #F7524) and Penicillin/Streptomycin (ThermoFisher, #15140-122). Both mesenchymal cells and organoids were cultured in 37°C with 21% O_2_ and 5% CO_2_. When required cells were grown in 3% FBS in the absence of WNT CM.

#### Mouse models

Mouse experiments were performed under the Animal (Scientific Procedures) Act 1986 Amendment Regulations 2012 following ethical review by the University of Cambridge Animal Welfare and Ethical Review Body (AWERB). In addition, mouse experiments conducted in Germany were performed in accordance with the German animal welfare legislation and in strict pathogen-free conditions in the animal facility of the MPI-CBG. Protocols were approved by the Institutional Animal Welfare Officer (Tierschutzbeauftragter), and all necessary licenses were obtained from the regional Ethical Commission for Animal Experimentation of Dresden, Germany (Tierversuchskommission, Landesdirektion Dresden).

Mouse lines *Rosa26-nTnG* [Gt(ROSA)26Sortm1(CAG-tdTomato^∗^,-EGFP^∗^)Ees] and *Rosa26-mTmG* [Gt(ROSA)26Sortm4(ACTB-tdTomato,-EGFP)Luo/J] were obtained from the Jackson Laboratory (JAX). The *Rosa26-nGFP* line was obtained by germline recombination of the *Rosa26-nTnG* using a ubiquitous Cre. The Lgr5iresCreERT/RosatdTom was described in Huch et al. (2013) and kindly donated by Prof Hans Clevers (Hubrecht Institute). The Hes1-GFP was reported in Klinck et al. (2011) and kindly donated by Prof Anne Grapin-Botton (MPI-CBG). The *Pdgfra-H2B-GFP* was described in Hamilton et al. (2003) and obtained from Prof Magdalena Zernicka-Goetz. The *Pdgfra-H2B-GFP* mTmG mouse was obtained by crossing the *Pdgfra-H2B-GFP* and *Rosa26-mTmG* strains.

Mice were kept under standard husbandry in a pathogen-free environment with a 12 h day/night cycle. Sterile food and water were given *ad libitum*. Healthy adult mice (8-12 weeks of age) of both sexes were used for experiments. To induce liver damage, 8-12 weeks old mice were transferred to individual wheat-free cages and were fed with diet pellets supplemented with 0.1% DDC (3,5-diethoxycarbonyl-1,4- dihydrocollidine) (Custom Animal diets, LLC, #AD5001). Littermates from up to 3 litters of similar age and both sexes were randomly assigned to experimental groups. The diet was provided *ad libitum* for the duration of the experiment (up to 5 days or until weight drop reached a maximum of 20%), after which the mice were either sacrificed (DDC day 5) or switched back to normal chow to allow recovery (DDC day 5+6 days, DDC day 5+38 days). Untreated mice were used as controls (DDC Ctrl day 0). For each condition in each separate experiment, n = 3 mice were used, plus one additional mouse (n = 4) for the recovery groups to account any potential unexpected deaths. No mice were excluded from analysis.

### Method details

#### Liver ductal isolation

To enrich for the biliary duct compartment, mouse livers were harvested and digested enzymatically as previously reported (Huch et al., 2013). In short, minced livers were incubated in a solution containing 0.0125% (mg/ml) collagenase (Merck/Sigma, #C9407), 0.0125% (mg/ml) dispase II (ThermoFisher, #17105-041) and 1% fetal bovine serum (FBS) (Merck/Sigma, #F7524) in DMEM/Glutamax (ThermoFisher, #31966-021) supplemented with HEPES (ThermoFisher, #15630-056) and Penicillin/Streptomycin (ThermoFisher, #15140-122) and 0.1 mg/ml of DNAase (Merck/Sigma, #DN25) in a shaker at 37°C and 150 rpm for 3h as detailed in Broutier et al. (2016). The biliary tree fragments and associated stroma were then dissociated into single cells with TrypLE diluted to 5x (GIBCO, #A12177-01).

#### Flow cytometry

For live cell sorting, single cells were incubated with fluorophore-conjugated antibodies for 30min and FACS-sorted using MoFlo Legacy, Astrios (Beckman Coulter), BD FACSAria (BD Biosciences) or SH800S (SONY) cell sorters. Cells were sequentially gated based on size and granularity (forward scatter, FSC, versus side scatter, SSC) and singlets (FSC-Area versus FSC-Height); after which ductal cells (DC) were selected based on EpCAM positivity and negative exclusion of the hematopoietic/endothelial markers CD31, CD45 and CD11b. The mesenchyme was enriched based on SCA1 positivity from the EpCAM^-^CD31^-^CD45^-^CD11b^-^ fraction, or in the case of *Pdgfra-H2B-GFP* mice, as double positive PDGFRα-GFP^+^SCA1^+^ cells gated from the EpCAM^-^CD31^-^CD45^-^CD11b^-^ fraction. For the isolation of PF and HSC the SCA1^+^/SCA1^-^ fractions were subsequently gated on CD34^+^ (PF) and CD34^-^ (HSC). Cells derived from *Rosa26-nTnG* or *Rosa26-mTmG* livers were further gated for tdTomato positivity. DC from *Hes1-GFP* livers were sorted as EpCAM^+^CD31^-^CD45^-^CD11b^-^ regardless of GFP positivity.

For analysis of *Hes1-GFP* expression following culture in conditioned medium (CM), 30 000 *Hes1-GFP* organoid cells were cultured in mesenchymal CM from PDGFRα^+^SCA1^+^ cells or non-conditioned media control (refreshed every 48h) for 8 days. For analysis of *Hes1-GFP* expression upon cell-cell contact with mesenchymal cells, 30 000 *Hes1-GFP* organoid cells were seeded alone or with 30 000 tdTomato^+^SCA1^+^ mesenchymal cells in EM + WNT CM (refreshed every 48h) on 2D Matrigel-layered 48-well plates for 8 days. *Lgr5CreERT2*, *R26-tdTomato* organoids were derived from *Lgr5CreERT2*, *R26-tdTomato* mice. 30 000 *Lgr5CreERT2*, *R26-tdTomato* organoid cells were cultured alone or co-cultured with PDGFRα-GFP^+^SCA1^+^ Msc cells on 2D Matrigel-layered 48-well plates overlaid with EM + WNT CM medium. On day 3, cultures were incubated with 10 μM of 4-hydroxytamoxifen (4-HT, Merck/Sigma, #H7904-5MG) and analyzed 24h later. Prior to all flow cytometric analysis, cultures were extracted from Matrigel with Cell Recovery solution (Corning, #354253), dissociated into single cells and analyzed with a Fortessa cell analyzer (BD Bioscience).

#### Matrigel co-culture

For matrigel bubble co-cultures, 3600 or 5000 freshly isolated and FACS-sorted ductal cells were embedded in a 25 μ.L Matrigel bubble with mesenchymal cells. The varying number of mesenchymal cells is indicated in the figure or figure legend (4000, 5000, 18 000, 25 000, 50 000, or 100 000) The co-culture was overlayed with 250 μL of MM. The organoid formation efficiency was assessed at day 7 after seeding.

#### Conditioned medium and transwell co-cultures

To generate mesenchymal conditioned medium (CM), sorted mesenchymal cells (PDGFRα^+^SCA1^+^) were first expanded *in vitro* (up to passage 2 or 3) as detailed above. When reaching 80%–90% confluency, cells were incubated with fresh MM medium. This was conditioned for 48h, centrifuged at 500 g for 10 min and filtered prior to being added to freshly sorted EpCAM^+^ DC. For transwell co-cultures, freshly sorted or *in vitro* passaged mesenchymal cells were seeded on the bottom of 24 transwell-fitting plates (Corning, #3470) and cultured in MM medium for 5-7 days until reaching 80%–90% confluency. Freshly sorted 5000 EpCAM^+^ DC were then seeded on top on cell-impermeable transwell inserts within a 25 μL drop of 100% Matrigel. Both the top and bottom compartments of the transwell were maintained in either Basal or MM for 10 days.

#### 2D Matrigel co-cultures

For cell aggregation on 96–well plates pre-coated with a Matrigel-layer, single PDGFRα^+^SCA1^+^ cells and DC were mixed in the following mesenchyme-to- ductal cell ratios: 0:1, 0.1:1, 0.2:1, 0.5:1, 1:1, 1:2 and 5:1. In 96 well plate, for a 1:1 ratio co-culture 5000 DCs and 5000 Msc were used, and Msc adjusted accordingly in lower ratios (e.g., 5000 DCs and 2500 Msc in 0.5:1 ratio). After mixing, cells were centrifuged at 300 g for 5 min and seeded on top of a 2D-layer of solidified Matrigel (100%) covering the bottom of a 96-well plate. The medium of choice was dependent on experimental context, but consisted on either growth factor-reduced mesenchymal medium (MM) or complete organoid expansion medium (EM) supplemented with WNT CM to enhance mesenchymal cell survival. After 48h, organoids containing ductal and mesenchymal cells were detected.

#### Microfluidic chip production

Polydimethylsiloxane (PDMS) microfluidic chips were produced using soft lithography and replica molding as described elsewhere (Kleine-Brüggeney et al., 2019). Ductal and mesenchymal cells were co-encapsulated into microgels using a microfluidic flow-focusing device (FFD) that was a modified version of the microfluidic chip previously described in Kleine-Brüggeney et al. (2019) and Kumachev et al. (2011) and used to compartmentalize cells in droplets. Chips were designed to contain two separate inlets for the loading of two distinct cell populations (in aqueous phase): one inlet for the continuous phase (fluorinated oil HFE 7500 (Fluorochem, #051243)) containing 0.3% Pico Surf 1 surfactant (Sphere Fluidics, #C022) and one outlet. To maximize the chance of cell-cell encounters by proximity, the cross geometry of the chip, where droplet formation occurs, was limited to a width of 70 μm and a height of 75 μm. The AutoCAD Flow Focusing Device (FFD) Chip Design File used for production of PDMS FFD is available upon request.

#### Microfluidic cell encapsulation

EpCAM^+^ ductal cells and SCA1^+^ mesenchymal cells were isolated from *Rosa26-nGFP* and *Rosa26-nTnG* mice, respectively or vice versa, and were expanded *in vitro* as detailed above. The organoid and mesenchymal cell populations were dissociated into single cells, filtered through 40 μm cell strainers and resuspended as 0.75 × 10^5-6^ cells/50 μL of MM + Ri medium, respectively. The cell suspensions were mixed with ultralow melting agarose solution (3% SeaPrep®, LONZA, #50302) in a volume ratio of 1:1 and were loaded onto the two aqueous phase inlets of the FFD. A flow rate of 3 μl/min was used for both aqueous phase channels and a flow rate of 30 μl/min for the continuous phase. The nascent emulsion droplet containing liquid agarose and cell suspension was collected in an ice cooled test tube resulting in agarose microgel formation. The gels were subsequently demulsified with 45 μl 1H,1H,2H,2H Perfluoro-1-octanol (PFO) (Merck, #370533) into 200 μL of MM+ Ri medium. μ-slide 8-well dishes (ibidi, #80826) were layered with 130 μL of ice-cold Matrigel/well and 10-15 μL of the microgel/cell suspension was seeded within each well. The cultures were maintained in MM medium.

#### Small molecule inhibitor and siRNA treatment

For the small-molecule inhibitor experiments, 10 000 freshly sorted EpCAM^+^ DC were incubated in MM + Ri medium supplemented with one of the following inhibitors: A8301 (5 μM), SB431542 (10 μM), DAPT (10 μM), DBZ (10 μM) or Verteporfin (0.1 μM) or a combination of these, for 3h at 37°C. Cells treated with the same % of the vehicle DMSO were used as controls. The DC-treated cells were divided in half: 5000 cells were seeded alone as monoculture, 5000 were mixed with PDGFRα^+^SCA1^+.^in a 1:1 ratio. Cells were seeded in MM + Ri on top of a Matrigel-coated well in a 96wp as above. For the siRNA screen, 10 000 freshly sorted EpCAM^+^ DC were transfected with 10pmol of a pool of 4 ON-Targetplus siRNA (Dharmacon) (see Methods S1D) for each candidate gene using Lipofectamine RNAimax (Life Technologies, # 13778030) according to manufacturer’s instruction. Cells suspended in Basal + Ri medium were centrifuged for 45 minutes at 600 g at 32°C and then incubated 3h at 37°C. 5000 transfected DC were seeded alone, 5000 were co-cultured with PDGFRα^+^SCA1^+.^ mesenchymal cells at 0.5:1 ratio in MM + Ri on 2D Matrigel-layered 96wp. Organoid formation was assayed at d10.

#### Mouse tissue sections staining

For tissue staining, livers were washed in PBS, diced with a razor blade and fixed for 2h or overnight in 10% formalin while rolling at 4°C. Tissues were then incubated with 30% sucrose PBS for 24-48h, embedded into cryomolds (Sakura, #4566) with OCT compound (VWR, #361603E) and snap-frozen. Tissue blocks were cryo-sectioned with a Leica CM-3050S cryostat or on Thermo Scientific CryoStar NX70 cryostat. For Ki67 staining, thick liver sections (100 μm) were blocked/permeabilized in PBS containing 1% Triton X-100 (Merck/Sigma, #T8787), 5% dimethyl sulfoxide (DMSO; Merck/Sigma, #D8418), 1% bovine serum albumin (BSA; Merck/Sigma #A8806) and 2% donkey serum (DS; Merck/Sigma, #D9663) for 16h at 4°C, and incubated with primary antibodies diluted in PBS + 0.5% Triton X-100, 1% DMSO, 2% DS for 72h at 4°C on an orbital shaker. Tissues were washed thoroughly over 24h with PBS + 0.5% Triton X-100 and 1% DMSO and then incubated with fluorophore-conjugated secondary antibodies in PBS + 0.5% Triton X-100, 1% DMSO and 2% DS for 48 at 4°C (see Methods S1A and S1B). Tissues were counterstained in PBS containing 1:1000 Hoechst 33342 (ThermoFisher, # H3570) for 1h and then washed in ascending glycerol concentrations (10%, 30%, 50%, 70%, 90%) for 1h. Sections were mounted in Vectashield (Vector Laboratories, #H-1000-10). All other liver immunostainings were performed on thin (8 or 12 μm) sections. For detection of surface antigens (e.g., SCA1), sections were blocked in PBS with 2% DS and 1% BSA for 2h at RT, incubated with primary antibodies in 1/100-diluted blocking buffer overnight at 4°C and with secondary antibodies for 2h at RT in 0.05% BSA PBS. Sections were counterstained with 1:1000 Hoechst for 10min and mounted in Vectashield. The stainings for PDGFRα, VEGFR3, β-catenin, and PCK were all enhanced with an additional Tris-EDTA pH9 antigen retrieval step (3min, 65°C) prior to blocking. Non-membrane stains were performed as above but with a blocking buffer supplemented with 0.5% Triton X-100. Images were acquired using a confocal microscope (Leica SP8 or Zeiss LSM 880) and processed using Volocity software (PerkinElmer), ZEN software (Zeiss), or ImageJ/Fiji.

For spectral unmixing of SCA1, OPN, and SiR DNA stainings in *Pdgfra-H2B-GFP mTmG* liver mouse section, 8 μm or 12 μm mouse tissue sections were imaged on Zeiss LSM 880 using a LD LCI Plan-Apochromat 40x glycerol immersion correction NA 1.2 objective (Zeiss). Laser lines at 405nm, 488nm, 561nm and 633nm were used to excite the fluorophores. Lambda mode scanning (detecting 410-687nm) was used to detect AF405, AF488, EGFP, tdTomato, AF633 and SiR-DNA. For all images, tile scans and z stacks were acquired with a step size of 1.1 μm and a pinhole of 20 airy unit. Images were taken at 1024x1024 voxel density with a line averaging of 8. Fluorophores and autofluorescence were unmixed into separate channels using the unmixing algorithm provided in the Zen software (Zeiss). Additionally, for each of the pictures a scan in the same Z stack was acquired using usual confocal set up as a control. Single stained slides were used to obtain the reference spectra of the different fluorophores. All composite pictures from stained tissue sections were obtained by merging the single channel images in FIJI/ImageJ.

Refer to Methods S1A and S1B for the complete list of primary antibody dilutions and secondary antibodies used.

#### Organoid and mesenchyme staining

For *in vitro* stainings, organoids and/or co-cultures were first extracted from Matrigel to facilitate immunostaining with ice-cold Cell Recovery solution (Corning, #354253) and then fixed with 4% paraformaldehyde (PFA) (Electron Microscopy Sciences, #15713-S) for 30min at RT; alternatively, cells were fixed *in situ* to preserve mesenchymal-to-epithelial interactions. Blocking and permeabilization was performed for 2h at RT in PBS containing 0.5% Triton X-100, 2% DMSO, 1% BSA and 2% DS. EdU incorporation assays were performed with the Click-iT® EdU Alexa Fluor 594 Imaging Kit (Life Technologies, #C10339) according to the manufacturer’s protocol. Cells were incubated for 16h with 10 μM EdU in their respective culture medium, after which they were fixed in 4% PFA for 30 min, permeabilized with 0.5% Triton X-100 for 20 min and incubated with freshly prepared 1X Click-iT EdU cocktail for 30 min at RT. Nuclei were stained with Hoechst 33342 (Life Technologies, # 23491-52-3), DAPI (BD-Biosciences, # BD564907) or SiR-DNA (Spirochrome, # CY-SC007) for 15 min.

For Cleaved Caspase 3, pSer10-Histone3 and Krt19 staining, organoids were fixed with 4% PFA in Matrigel for 30min on ice, washed in 0.01% Triton X-100 PBS and permeabilized in 0.2% Triton X-100 PBS for 30min at RT. After 1hr blocking in 3% BSA 0.01% Triton X-100 PBS, the samples were incubated with primary antibodies overnight at 4°C in blocking solution. Following 3 washes with 0.01% Triton X-100 PBS, the samples were incubated 1hr at RT with secondary antibodies and Phalloidin/DAPI in blocking solution.

For live/dead staining in live cultures, Viability/Cytotoxicity Assay Kit for Animal Live & Dead Cells (Biotium, #30002) was used according to the manufacturer’s protocol. Briefly, 2 μM Calcein and 4 μM EthDIII in PBS was added to cover the cells, and incubated for 30 min before washing, and imaging in normal media. Immunofluorescence images were acquired using a confocal microscope (Leica SP8 or Zeiss LSM 880) and processed using Volocity software (PerkinElmer), ZEN software (Zeiss), or ImageJ/Fiji. Live cell images were acquired in a Leica DMIL LED (brightfield only) using a Leica DF C450C camera or an EVOS FL (brightfield and fluorescence) microscope. Whole well pictures were acquired with a Leica M80 microscope using a Leica MC170 HD camera.

All composite pictures from stained cultures or organoids were obtained by merging the single channel images in FIJI/ImageJ.

#### Time-lapse imaging and processing

Time-lapse imaging of cells was carried out at 37°C and 5% CO_2_ for 24h periods. A 20x air objective on a spinning- disk confocal microscope system (Intelligent Imaging Innovations, Inc. 3i) comprising an Observer Z1 inverted microscope (Zeiss), a CSU X1 spinning disk head (Yokogawa), and a QuantEM 512SC camera (Photometrics), was used to perform time-lapse imaging. Imaging was performed at 15 min intervals, with a z-step of 7 μm and a low laser power. A 10x air objective on a Zeiss 710 confocal microscope was also used perform time-lapse imaging at 15 min intervals, with a z-step of 9 μm, and 1024 × 1024 bidirectional scanning. Videos were generated with the Slidebook6 software and were analyzed with ImageJ/Fiji.

#### qRT-PCR

Total RNA was extracted from cells using the Arcturus PicoPure RNA Isolation Kit (Applied Biosystems, #12204-01) according to the manufacturer’s protocol; including a 15 min digestion step with DNase to remove traces of genomic DNA. The RNA (50-250 ng) was reverse-transcribed with the Moloney Murine Leukemia Virus reverse transcriptase (M-MLVRT) (Promega, #M368B) and amplified using the iTaqTM Universal SYBR Green Supermix (Bio-Rad, #172-5124) on the CFX ConnectTM Real-Time PCR Detection System (Bio-Rad) or using FastStart Essential DNA Green Master (Roche, #06402712001) on the LightCycler 96 machine (Roche). The list of primers used for qRT-PCR is provided in Supplementary Methods S1C. Gene expression levels were normalized to the housekeeping gene *Hprt*, *18S* or *Gapdh* as specified in the graph axis labels. Refer to Methods S1C for the complete list of primer sequences used.

#### RNA sequencing and analysis

DC (EpCAM^+^ CD45^-^ CD11b^-^ CD31^-^) and mesenchymal/stromal sub-population (PDGFRα^+^/^-^ SCA1^+^/^-^ CD45^-^ CD11b^-^ CD31^-^) hepatic fractions were sorted from three healthy mouse littermates for analysis of gene expression in homeostasis. For co-culture analyses, mesenchymal cells (SCA1^+^ CD45^-^ CD11b^-^ CD31^-^) from two littermates were first expanded on the bottom of 24 transwell-fitting plates (50000 cells/well) for 7 days in MM medium, after which freshly sorted DC (EpCAM^+^ CD45^-^ CD11b^-^ CD31^-^) from two other littermates were cultured on a cell-impermeable transwell insert (5000 cells/Matrigel bubble) alone in EM or in MM with the mesenchymal cells at the bottom for 15 days. Total RNA was extracted from all samples with the Picopure RNA Extraction Kit according to manufacturer’s instructions (including DNase digestion).

RNA libraries were prepared using Smartseq2 (Picelli et al., 2014) and were sequenced on an Illumina HiSeq 4000 or Illumina HiSeq1500 instrument in single read mode at 50 base length. FastQC (version 0.11.4) was used for initial quality control of the reads. Reads were then mapped to the GRCm38/mm10 UCSC reference genome using STAR aligner (version 2.5.0a). Samtools was used to filter unmapped and low-quality reads (-F 1804 and -q 20). Raw counts were generated using featureCounts from the Rsubread package (version 1.24.2) including all exons for a gene from the mm10 GTF file (Mus_musculus.GRCm38.87.gtf). RPKMs were generated with raw counts and gene lengths reported by featureCounts. TPM (transcripts per million) and log_2_(TPM+1) values were generated by normalizing the RPKM values. Dendograms were generated using hclust from the R stats package (version 3.5.1). Scaled RPKM values were used with Euclidean distance and the ward.d method for performing hierarchical clustering. For clustering of all samples, the top 2 000 most variable genes were used. Heatmaps were prepared based on TPM and logTPM values using the Prism9 software. All data has been deposited in GEO database. GEO accession number GSE140697 .

#### Mesenchymal scRNaseq

Data was obtained from Dobie et al. (2019) and analyzed for the expression of specific genes as detailed in their methods section (Dobie et al., 2019).

### Quantification and statistical analysis

#### Organoid formation efficiency and size

Organoid formation efficiency was quantified by counting the total number of cystic/single layer (lumen-containing) organoid structures after 7-10 days in culture and normalizing it to the total number of EpCAM^+^ cells seeded (typically 5000). Organoids were selected as regions of interest (ROI) with the blow/lasso tool and measured for area using Fiji (Schindelin et al., 2012).

#### Liver section analysis and processing

In order to quantify the relative positions of OPN^+^ ductal cells from PDGFRα^+^SCA1^+^ mesenchymal cells in liver tissue slices we developed Liver Cell Distances, a custom pipeline for Fiji implemented as a Jython script. Liver Cell Distances generates signal masks from maximum intensity z-projections using parameter sets appropriate for the size and morphology of the labeled structures of interest (Methods S1E). Single channel masks are combined to create SCA1/GFP and Hoescht/OPN double labeled area masks, allowing extraction of areas expressing SCA1 and GFP, and nuclei expressing OPN. To allow unsupervised use of automatic thresholding methods on images with varying signal levels including those with only background present, minimum intensity values can be set to discard mask areas containing raw mean intensity values too low to be signal of interest.

Distances from each OPN labeled nucleus with an area of at least 5 μm^2^ to the nearest SCA1/GFP area are calculated by measuring the mean value of the SCA1/GFP signed Euclidean distance transform inside the nucleus. Liver Cell Distances script has been deposited in the publicly available GitHub repository: https://github.com/gurdon-institute/Liver-Cell-Distances. The analysis of liver sections with Desmin staining has been performed manually. The Desmin pictures have been denoised with Noise2Void (Krull et al., 2020), and were deconvolved with Huygens Professional version 19.04 (Scientific Volume Imaging, the Netherlands, https://svi.nl/), using a theoretical PSF and the CMLE algorithm with a SNR:20, 0.05 quality threshold and for a maximum of 40 iterations.

#### Fluorescence analysis

In order to quantify Hes1-GFP and tdTomato fluorescence within organoid structures, we developed Chimeric Organoid Analyzer, a script for Fiji that automatically applies custom segmentation pipelines for each of the image channels. Chimeric Organoid Analyzer measures organoid area in a single slice of a z stack chosen for optimal focus and measures the area of GFP and Tomato signal inside the organoid. Organoid area is mapped by calculating smoothed local variance and applying the Triangle automatic thresholding method (Zack et al., 1977). GFP signal is segmented using the Otsu threshold (Otsu, 1979) on smoothed signal, and Tomato-containing cell clusters are segmented using Kapur’s maximum entropy threshold (Kapur et al., 1985) on difference of Gaussians processed images. These methods were chosen to detect the features of interest in each channel, namely textured regions, large, homogeneous signal areas and discrete clusters of cells in the brightfield, GFP and Tomato channels respectively. Chimeric Organoid Analyzer has been deposited in the publicly available GitHub repository: https://github.com/gurdon-institute/Chimeric-Organoid-Analyser.

#### Statistics

Data were analyzed as detailed in Figure legends and as appropriate for each experiment by using Mann–Whitney test, Welch’s t test, unpaired t test with Welch’s correction or a Student’s t test. p < 0.05 was considered statistically significant. Calculations were performed using the Prism 9 software package. All P values are given in the corresponding figure legends. Dispersion and precision measures (e.g., mean, median, SD, SEM) are specified in the figure legends. All the independent and biological replicates are specified in figure legends and Supplementary Table S1. Additionally, we provide Table S1 with all the manual quantification data.

## Data Availability

The RNaseq datasets generated during this study are available at Gene Expression Omnibus (https://www.ncbi.nlm.nih.gov/geo/info/seq.html) under accession numbers GEO: GSE140697 . Software/packages used to analyze the dataset are either freely or commercially available. The custom scripts described in this manuscript are deposited on Github repository. Noise To Void: https://github.com/juglab/n2v Liver Cell Distances: https://github.com/gurdon-institute/Liver-Cell-Distances Chimeric Organoid Analyzer: https://github.com/gurdon-institute/Chimeric-Organoid-Analyser

## Supplemental information

Document S1 Figures S1–S7

Table S1 All manual quantification values outlined as separate tabs related to each relevant figure, related to Figures 1, 2, 3, 4, 5, 6, 7, S1–S7, and STAR Methods

Methods S1 Related to STAR Methods S1A: List of primary antibodies; S1B: List of secondary antibodies; S1C: qRT-PCR primers; S1D: siRNA oligos; S1E: Parameters of FIJI script analysis.

Data S1 RNA-sequencing analysis and gene list, related to Figures 2 and 4

Document S2 Article plus supplemental information

## References

[bib1] Aloia L., McKie M.A., Vernaz G., Cordero-Espinoza L., Aleksieva N., van den Ameele J., Antonica F., Font-Cunill B., Raven A., Aiese Cigliano R. (2019). Epigenetic remodelling licences adult cholangiocytes for organoid formation and liver regeneration. Nat. Cell Biol..

[bib2] Apte U., Thompson M.D., Cui S., Liu B., Cieply B., Monga S.P. (2008). Wnt/beta-catenin signaling mediates oval cell response in rodents. Hepatology.

[bib3] Berger D.R., Ware B.R., Davidson M.D., Allsup S.R., Khetani S.R. (2015). Enhancing the functional maturity of induced pluripotent stem cell-derived human hepatocytes by controlled presentation of cell-cell interactions in vitro. Hepatology.

[bib4] Bhatia S.N., Balis U.J., Yarmush M.L., Toner M. (1998). Microfabrication of hepatocyte/fibroblast co-cultures: role of homotypic cell interactions. Biotechnol. Prog..

[bib5] Boulter L., Govaere O., Bird T.G., Radulescu S., Ramachandran P., Pellicoro A., Ridgway R.A., Seo S.S., Spee B., Van Rooijen N. (2012). Macrophage-derived Wnt opposes Notch signaling to specify hepatic progenitor cell fate in chronic liver disease. Nat. Med..

[bib6] Boulter L., Lu W.Y., Forbes S.J. (2013). Differentiation of progenitors in the liver: a matter of local choice. J. Clin. Invest..

[bib7] Broutier L., Andersson-Rolf A., Hindley C.J., Boj S.F., Clevers H., Koo B.K., Huch M. (2016). Culture and establishment of self-renewing human and mouse adult liver and pancreas 3D organoids and their genetic manipulation. Nat. Protoc..

[bib8] Choi T.Y., Ninov N., Stainier D.Y., Shin D. (2014). Extensive conversion of hepatic biliary epithelial cells to hepatocytes after near total loss of hepatocytes in zebrafish. Gastroenterology.

[bib9] Christen B., Slack J.M. (1999). Spatial response to fibroblast growth factor signalling in Xenopus embryos. Development.

[bib10] Clayton E., Forbes S.J. (2009). The isolation and in vitro expansion of hepatic Sca-1 progenitor cells. Biochem. Biophys. Res. Commun..

[bib11] Cohen M., Georgiou M., Stevenson N.L., Miodownik M., Baum B. (2010). Dynamic filopodia transmit intermittent Delta-Notch signaling to drive pattern refinement during lateral inhibition. Dev. Cell.

[bib12] Coll M., Perea L., Boon R., Leite S.B., Vallverdú J., Mannaerts I., Smout A., El Taghdouini A., Blaya D., Rodrigo-Torres D. (2018). Generation of Hepatic Stellate Cells from Human Pluripotent Stem Cells Enables In Vitro Modeling of Liver Fibrosis. Cell Stem Cell.

[bib13] Cordero-Espinoza L., Huch M. (2018). The balancing act of the liver: tissue regeneration versus fibrosis. J. Clin. Invest..

[bib14] Davidson M.D., Kukla D.A., Khetani S.R. (2017). Microengineered cultures containing human hepatic stellate cells and hepatocytes for drug development. Integr. Biol..

[bib15] De Joussineau C., Soulé J., Martin M., Anguille C., Montcourrier P., Alexandre D. (2003). Delta-promoted filopodia mediate long-range lateral inhibition in Drosophila. Nature.

[bib16] Dobie R., Wilson-Kanamori J.R., Henderson B.E.P., Smith J.R., Matchett K.P., Portman J.R., Wallenborg K., Picelli S., Zagorska A., Pendem S.V. (2019). Single-Cell Transcriptomics Uncovers Zonation of Function in the Mesenchyme during Liver Fibrosis. Cell Rep..

[bib17] Furuyama K., Kawaguchi Y., Akiyama H., Horiguchi M., Kodama S., Kuhara T., Hosokawa S., Elbahrawy A., Soeda T., Koizumi M. (2011). Continuous cell supply from a Sox9-expressing progenitor zone in adult liver, exocrine pancreas and intestine. Nat. Genet..

[bib18] Gurtner G.C., Werner S., Barrandon Y., Longaker M.T. (2008). Wound repair and regeneration. Nature.

[bib19] Hamilton T.G., Klinghoffer R.A., Corrin P.D., Soriano P. (2003). Evolutionary divergence of platelet-derived growth factor alpha receptor signaling mechanisms. Mol. Cell. Biol..

[bib20] Hofmann J.J., Zovein A.C., Koh H., Radtke F., Weinmaster G., Iruela-Arispe M.L. (2010). Jagged1 in the portal vein mesenchyme regulates intrahepatic bile duct development: insights into Alagille syndrome. Development.

[bib21] Hu M., Kurobe M., Jeong Y.J., Fuerer C., Ghole S., Nusse R., Sylvester K.G. (2007). Wnt/beta-catenin signaling in murine hepatic transit amplifying progenitor cells. Gastroenterology.

[bib22] Huch M., Dorrell C., Boj S.F., van Es J.H., Li V.S., van de Wetering M., Sato T., Hamer K., Sasaki N., Finegold M.J. (2013). In vitro expansion of single Lgr5+ liver stem cells induced by Wnt-driven regeneration. Nature.

[bib23] Huch M., Gehart H., van Boxtel R., Hamer K., Blokzijl F., Verstegen M.M., Ellis E., van Wenum M., Fuchs S.A., de Ligt J. (2015). Long-term culture of genome-stable bipotent stem cells from adult human liver. Cell.

[bib24] Jeliazkova P., Jörs S., Lee M., Zimber-Strobl U., Ferrer J., Schmid R.M., Siveke J.T., Geisler F. (2013). Canonical Notch2 signaling determines biliary cell fates of embryonic hepatoblasts and adult hepatocytes independent of Hes1. Hepatology.

[bib25] Joe A.W., Yi L., Natarajan A., Le Grand F., So L., Wang J., Rudnicki M.A., Rossi F.M. (2010). Muscle injury activates resident fibro/adipogenic progenitors that facilitate myogenesis. Nat. Cell Biol..

[bib26] Kan N.G., Junghans D., Izpisua Belmonte J.C. (2009). Compensatory growth mechanisms regulated by BMP and FGF signaling mediate liver regeneration in zebrafish after partial hepatectomy. FASEB J..

[bib27] Kapur J.N., Sahoo P.K., Wong A.K.C. (1985). A New Method for Gray-Level Picture Thresholding Using the Entropy of the Histogram. Comput Vision Graph.

[bib28] Katsumata L.W., Miyajima A., Itoh T. (2017). Portal fibroblasts marked by the surface antigen Thy1 contribute to fibrosis in mouse models of cholestatic liver injury. Hepatol. Commun..

[bib29] Klein A.M., Simons B.D. (2011). Universal patterns of stem cell fate in cycling adult tissues. Development.

[bib30] Kleine-Brüggeney H., van Vliet L.D., Mulas C., Gielen F., Agley C.C., Silva J.C.R., Smith A., Chalut K., Hollfelder F. (2019). Long-Term Perfusion Culture of Monoclonal Embryonic Stem Cells in 3D Hydrogel Beads for Continuous Optical Analysis of Differentiation. Small.

[bib31] Klinck R., Füchtbauer E.M., Ahnfelt-Rønne J., Serup P., Jensen J.N., Jørgensen M.C. (2011). A BAC transgenic Hes1-EGFP reporter reveals novel expression domains in mouse embryos. Gene Expr. Patterns.

[bib32] Krull A.V.T., Prakash M., Lalit M., Jug F. (2020). Probabilistic Noise2Void: Unsupervised Content-Aware Denoising. Front. Comput. Sci..

[bib33] Kumachev A., Greener J., Tumarkin E., Eiser E., Zandstra P.W., Kumacheva E. (2011). High-throughput generation of hydrogel microbeads with varying elasticity for cell encapsulation. Biomaterials.

[bib34] Lepreux S., Desmoulière A. (2015). Human liver myofibroblasts during development and diseases with a focus on portal (myo)fibroblasts. Front. Physiol..

[bib35] Li C.Y., Wood D.K., Huang J.H., Bhatia S.N. (2013). Flow-based pipeline for systematic modulation and analysis of 3D tumor microenvironments. Lab Chip.

[bib36] Malato Y., Naqvi S., Schürmann N., Ng R., Wang B., Zape J., Kay M.A., Grimm D., Willenbring H. (2011). Fate tracing of mature hepatocytes in mouse liver homeostasis and regeneration. J. Clin. Invest..

[bib37] Mederacke I., Hsu C.C., Troeger J.S., Huebener P., Mu X., Dapito D.H., Pradere J.P., Schwabe R.F. (2013). Fate tracing reveals hepatic stellate cells as dominant contributors to liver fibrosis independent of its aetiology. Nat. Commun..

[bib38] Miyajima A., Tanaka M., Itoh T. (2014). Stem/progenitor cells in liver development, homeostasis, regeneration, and reprogramming. Cell Stem Cell.

[bib39] Nguyen T.V., Ukairo O., Khetani S.R., McVay M., Kanchagar C., Seghezzi W., Ayanoglu G., Irrechukwu O., Evers R. (2015). Establishment of a hepatocyte-kupffer cell coculture model for assessment of proinflammatory cytokine effects on metabolizing enzymes and drug transporters. Drug Metab. Dispos..

[bib40] Otsu N. (1979). Threshold Selection Method from Gray-Level Histograms. Ieee T Syst Man Cyb.

[bib41] Ouchi R., Togo S., Kimura M., Shinozawa T., Koido M., Koike H., Thompson W., Karns R.A., Mayhew C.N., McGrath P.S. (2019). Modeling Steatohepatitis in Humans with Pluripotent Stem Cell-Derived Organoids. Cell Metab..

[bib42] Patel D., Haque A., Gao Y., Revzin A. (2015). Using reconfigurable microfluidics to study the role of HGF in autocrine and paracrine signaling of hepatocytes. Integr. Biol..

[bib43] Perrimon N., Pitsouli C., Shilo B.Z. (2012). Signaling mechanisms controlling cell fate and embryonic patterning. Cold Spring Harb. Perspect. Biol..

[bib44] Picelli S., Faridani O.R., Björklund A.K., Winberg G., Sagasser S., Sandberg R. (2014). Full-length RNA-seq from single cells using Smart-seq2. Nat. Protoc..

[bib45] Pintilie D.G., Shupe T.D., Oh S.H., Salganik S.V., Darwiche H., Petersen B.E. (2010). Hepatic stellate cells’ involvement in progenitor-mediated liver regeneration. Lab. Invest..

[bib46] Prior N., Inacio P., Huch M. (2019). Liver organoids: from basic research to therapeutic applications. Gut.

[bib47] Ramachandran P., Dobie R., Wilson-Kanamori J.R., Dora E.F., Henderson B.E.P., Luu N.T., Portman J.R., Matchett K.P., Brice M., Marwick J.A. (2019). Resolving the fibrotic niche of human liver cirrhosis at single-cell level. Nature.

[bib48] Raven A., Lu W.Y., Man T.Y., Ferreira-Gonzalez S., O’Duibhir E., Dwyer B.J., Thomson J.P., Meehan R.R., Bogorad R., Koteliansky V. (2017). Cholangiocytes act as facultative liver stem cells during impaired hepatocyte regeneration. Nature.

[bib49] Rockey D.C., Boyles J.K., Gabbiani G., Friedman S.L. (1992). Rat hepatic lipocytes express smooth muscle actin upon activation in vivo and in culture. J. Submicrosc. Cytol. Pathol..

[bib50] Rossi J.M., Dunn N.R., Hogan B.L., Zaret K.S. (2001). Distinct mesodermal signals, including BMPs from the septum transversum mesenchyme, are required in combination for hepatogenesis from the endoderm. Genes Dev..

[bib51] Schindelin J., Arganda-Carreras I., Frise E., Kaynig V., Longair M., Pietzsch T., Preibisch S., Rueden C., Saalfeld S., Schmid B. (2012). Fiji: an open-source platform for biological-image analysis. Nat. Methods.

[bib68] Schneider C., Rasband W., Eliceiri K. (2012). NIH Image to ImageJ: 25 years of image analysis. Nat. Methods.

[bib52] Shen K., Chang W., Gao X., Wang H., Niu W., Song L., Qin X. (2011). Depletion of activated hepatic stellate cell correlates with severe liver damage and abnormal liver regeneration in acetaminophen-induced liver injury. Acta Biochim. Biophys. Sin. (Shanghai).

[bib53] Sparks E.E., Huppert K.A., Brown M.A., Washington M.K., Huppert S.S. (2010). Notch signaling regulates formation of the three-dimensional architecture of intrahepatic bile ducts in mice. Hepatology.

[bib54] Stine R.R., Matunis E.L. (2013). Stem cell competition: finding balance in the niche. Trends Cell Biol..

[bib55] Takase H.M., Itoh T., Ino S., Wang T., Koji T., Akira S., Takikawa Y., Miyajima A. (2013). FGF7 is a functional niche signal required for stimulation of adult liver progenitor cells that support liver regeneration. Genes Dev..

[bib56] Takebe T., Sekine K., Enomura M., Koike H., Kimura M., Ogaeri T., Zhang R.R., Ueno Y., Zheng Y.W., Koike N. (2013). Vascularized and functional human liver from an iPSC-derived organ bud transplant. Nature.

[bib57] Takebe T., Enomura M., Yoshizawa E., Kimura M., Koike H., Ueno Y., Matsuzaki T., Yamazaki T., Toyohara T., Osafune K. (2015). Vascularized and Complex Organ Buds from Diverse Tissues via Mesenchymal Cell-Driven Condensation. Cell Stem Cell.

[bib58] Takeichi M. (2011). Self-organization of animal tissues: cadherin-mediated processes. Dev. Cell.

[bib59] Taymour R., Kilian D., Ahlfeld T., Gelinsky M., Lode A. (2021). 3D bioprinting of hepatocytes: core-shell structured co-cultures with fibroblasts for enhanced functionality. Sci. Rep..

[bib60] Upadhyay G. (2019). Emerging Role of Lymphocyte Antigen-6 Family of Genes in Cancer and Immune Cells. Front. Immunol..

[bib61] Ware B.R., Durham M.J., Monckton C.P., Khetani S.R. (2017). A Cell Culture Platform to Maintain Long-term Phenotype of Primary Human Hepatocytes and Endothelial Cells. Cell. Mol. Gastroenterol. Hepatol..

[bib62] Wei X., Zhang L., Zhou Z., Kwon O.J., Zhang Y., Nguyen H., Dumpit R., True L., Nelson P., Dong B. (2019). Spatially Restricted Stromal Wnt Signaling Restrains Prostate Epithelial Progenitor Growth through Direct and Indirect Mechanisms. Cell Stem Cell.

[bib63] Yang J., Mowry L.E., Nejak-Bowen K.N., Okabe H., Diegel C.R., Lang R.A., Williams B.O., Monga S.P. (2014). β-catenin signaling in murine liver zonation and regeneration: a Wnt-Wnt situation!. Hepatology.

[bib64] Zack G.W., Rogers W.E., Latt S.A. (1977). Automatic measurement of sister chromatid exchange frequency. J. Histochem. Cytochem..

[bib65] Zaret K.S. (2002). Regulatory phases of early liver development: paradigms of organogenesis. Nat. Rev. Genet..

[bib66] Zong Y., Panikkar A., Xu J., Antoniou A., Raynaud P., Lemaigre F., Stanger B.Z. (2009). Notch signaling controls liver development by regulating biliary differentiation. Development.

[bib67] Zorn A.M. (2008). StemBook.

