# Supplementary material for: Dynamic cell contacts between periportal mesenchyme and ductal epithelium act as a rheostat for liver cell proliferation
Source: Cell Stem Cell. 2021 Nov 4;28(11):1907–1921.e8. doi: 10.1016/j.stem.2021.07.002 (PMC8577825; doi:10.1016/j.stem.2021.07.002)
Supplement: Document S1 — Figures S1–S7 [file mmc1.pdf]

Cell Stem Cell, Volume *28*

Supplemental Information

Dynamic cell contacts between periportal mesenchyme and ductal epithelium act as a rheostat for liver cell proliferation

Lucía Cordero-Espinoza, Anna M. Dowbaj, Timo N. Kohler, Bernhard Strauss, Olga Sarlidou, German Belenguer, Clare Pacini, Nuno P. Martins, Ross Dobie, John R. Wilson-Kanamori, Richard Butler, Nicole Prior, Palle Serup, Florian Jug, Neil C. Henderson, Florian Hollfelder, and Meritxell Huch

Cordero-Espinoza et al._Figure S1

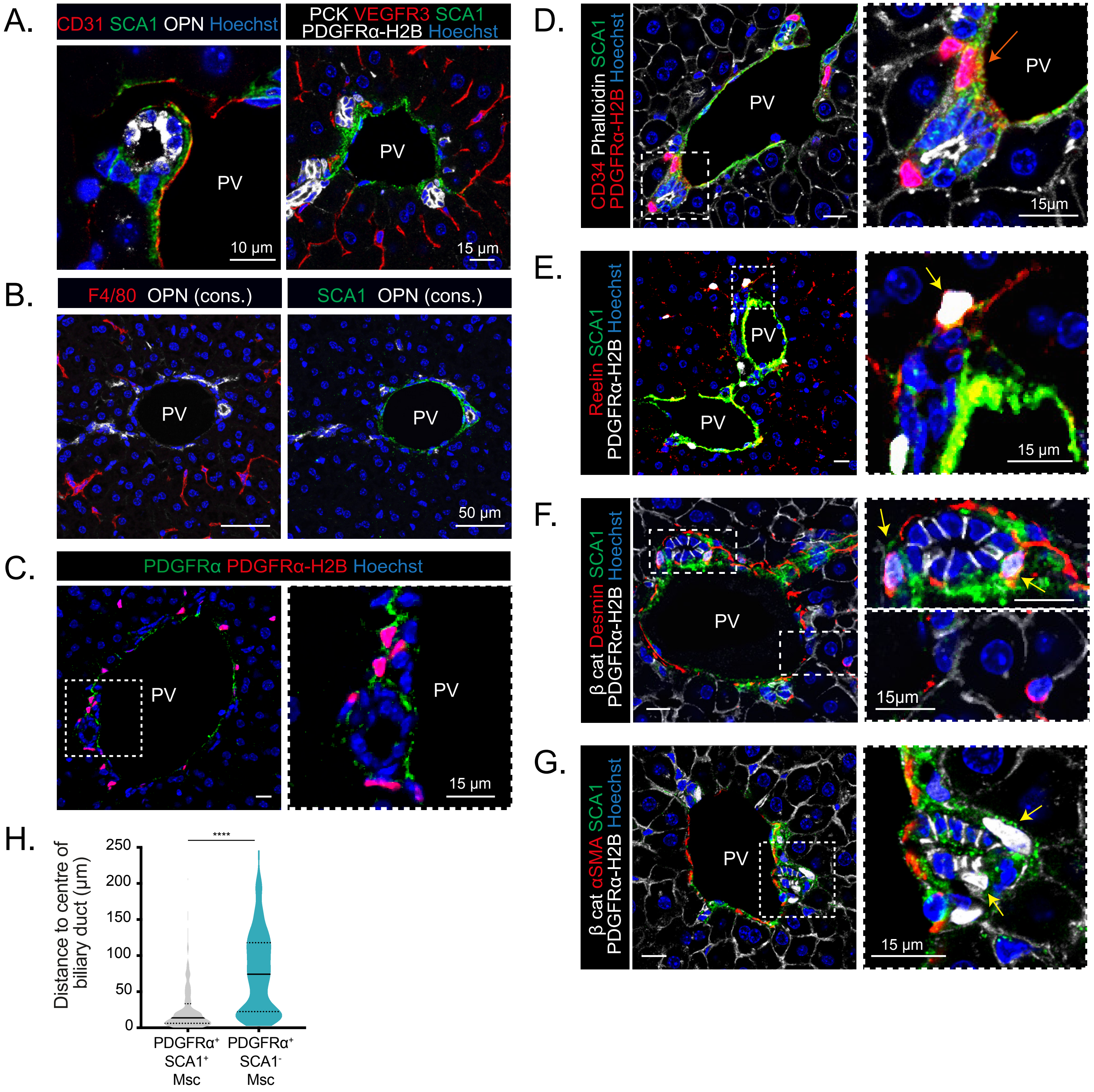

Figure S1. Periportal SCA1$^+$ cells express mesenchymal markers and are close to the bile duct epithelium. Related to Figure 1.

A-G) Immunofluorescence analysis of WT (A left, B) and Pdgfra-H2B-GFP (A right, C-G) mouse livers indicates that the PDGFRα$^+$SCA1$^+$ cell population at the portal tract co-stains with CD34, desmin and Reelin, but not with α-SMA, CD31, VEGFR3, and F4/80. Images are presented as single z-stacks and nuclei are all counterstained with Hoechst (blue). PV, portal vein. Composite pictures were obtained by merging the single channel images in FIJI/ImageJ.

A) SCA1 (green) immunostaining with CD31 (red, left panel) and VEGFR3 (red, right panel) endothelial markers, and the ductal cell markers Osteopontin (OPN, white, left panel) or pancytokeratin (PCK, white membrane, right panel).

B) Consecutive (cons) 5µm-liver sections stained with the ductal marker Osteopontin (OPN, white) and the macrophage marker F4/80 (red) (left panel) or SCA1 (green) (right panel).

C) PDGFRα immunostaining (green) in Pdgfra-H2B-GFP (nuclear red) mouse livers indicate that the reporter faithfully recapitulates endogenous PDGFRα expression.

D) Representative single z-stack images of Pdgfra-H2B-GFP (nuclear red) mouse livers co-stained with SCA1 (green), the portal fibroblast marker CD34 (red) and the actin marker Phalloidin (white, membrane). Orange arrow, PDGFRα$^+$SCA1$^+$ Msc.

E) SCA1 (green) immunostaining with the hepatic stellate cell marker Reelin (red) in Pdgfra-H2B-GFP (white) mouse livers. Yellow arrow, PDGFRα$^+$SCA1$^+$ Msc.

F) Representative single z-stack images of Pdgfra-H2B-GFP (nuclear white) mouse livers co-stained with SCA1 (green), mesenchymal marker Desmin (red) and epithelial marker β-catenin (white, membrane). Yellow arrows, PDGFRα$^+$SCA1$^+$ Msc.

G) SCA1 (green) immunostaining with the pericyte marker α-SMA (red) and the epithelial marker β-catenin (white, membrane) in Pdgfra-H2B-GFP (white, nuclear) mouse livers. Yellow arrows, PDGFRα$^+$SCA1$^+$ Msc.

H) Violin plot graph representing the distribution, median and IQR of distances between PDGFRα$^+$SCA1$^+$ Msc and PDGFRα$^+$SCA1$^-$ Msc to the center of the nearest biliary duct in homeostatic liver sections (n=3). P-value was calculated using Mann Whitney test, $p<0.0001$ (****).

Cordero-Espinoza et al._Figure S2

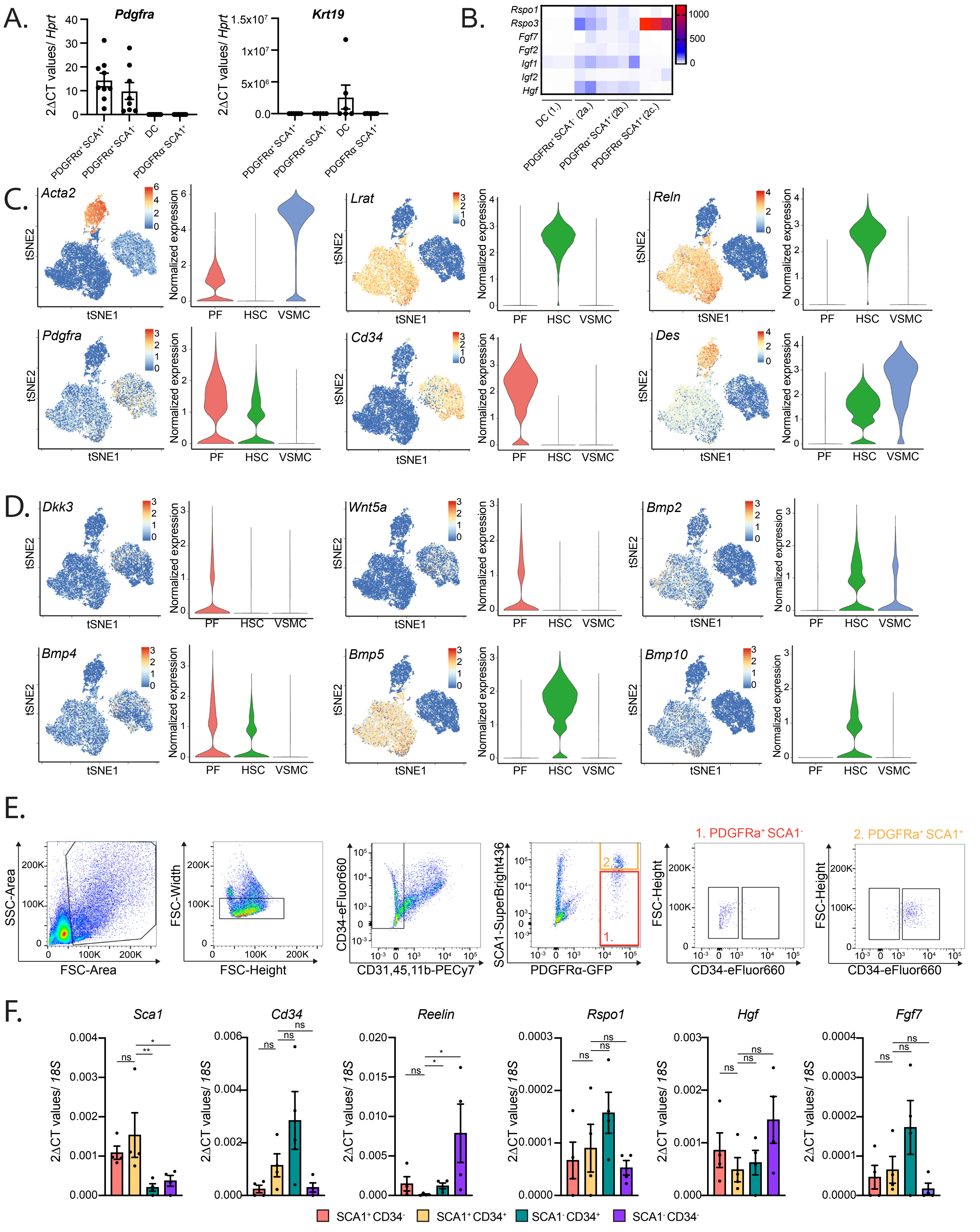

Figure S2. scRNAseq analysis on liver mesenchymal populations. Related to Figure 2.

A) mRNA expression levels of selected genes measured via RT-qPCR in freshly sorted DC and specified niche cells; Graph represents mean ± SEM on n=9 (Pdgfrα) or n=6 (Krt19) (DC, PDGFRα^{+}SCA1^{+}, PDGFRα^{-}SCA1^{+} Msc), n=8 (Pdgfrα) or n=5 (Krt19) (PDGFRα^{+}SCA1^{-} Msc) biological replicates (mice) from 3 independent experiments.

(B) Heatmap representing TPM values of the indicated secretome genes from n=3 biological replicates from the RNAseq analysis of ductal cells (DC), mesenchymal PDGFR^{+}SCA1^{+} and PDGFRα^{+}SCA1^{-} cells and stromal PDGFRα^{-}SCA1^{+} cells. The numbers (2a, 2b, 2c) correspond to sorted populations in Figure 2B.

C-D) scRNAseq analysis of sorted mouse hepatic mesenchymal cell populations published in Dobie *et al*., 2019. tSNE plots show the expression of the indicated genes in each mesenchymal cluster. Violin plots indicate the data point distribution of gene expression for the indicated genes. PF: portal fibroblasts, HSC: hepatic stellate cells. VSMC vascular smooth muscle cell.

(E) FACS sorting strategy to separate the portal fibroblasts from hepatic stellate cells (HSC) in the PDGFRα^{+}SCA1^{+} and PDGFRα^{+}SCA1^{-} fractions, based on the portal fibroblast specific marker CD34.

(F) qRT-PCR gene expression analysis of Sca1, Cd34 and Reelin markers, and selected secretome genes Rspo1, Hgf and Fgf7, in the specified mesenchymal subpopulations (PDGFRα^{+}SCA1^{+}CD34^{-}, red; PDGFRα^{+}SCA1^{+}CD34^{+}, yellow; PDGFRα^{+}SCA1^{-}CD34^{+}, green; PDGFRα^{+} SCA1^{-}CD34^{-}, purple). Graph represents the mean ± SEM of n=4 biological replicates in 3 independent experiments. p-values were obtained using Mann-Whitney test.

Cordero-Espinoza et al._Figure S3

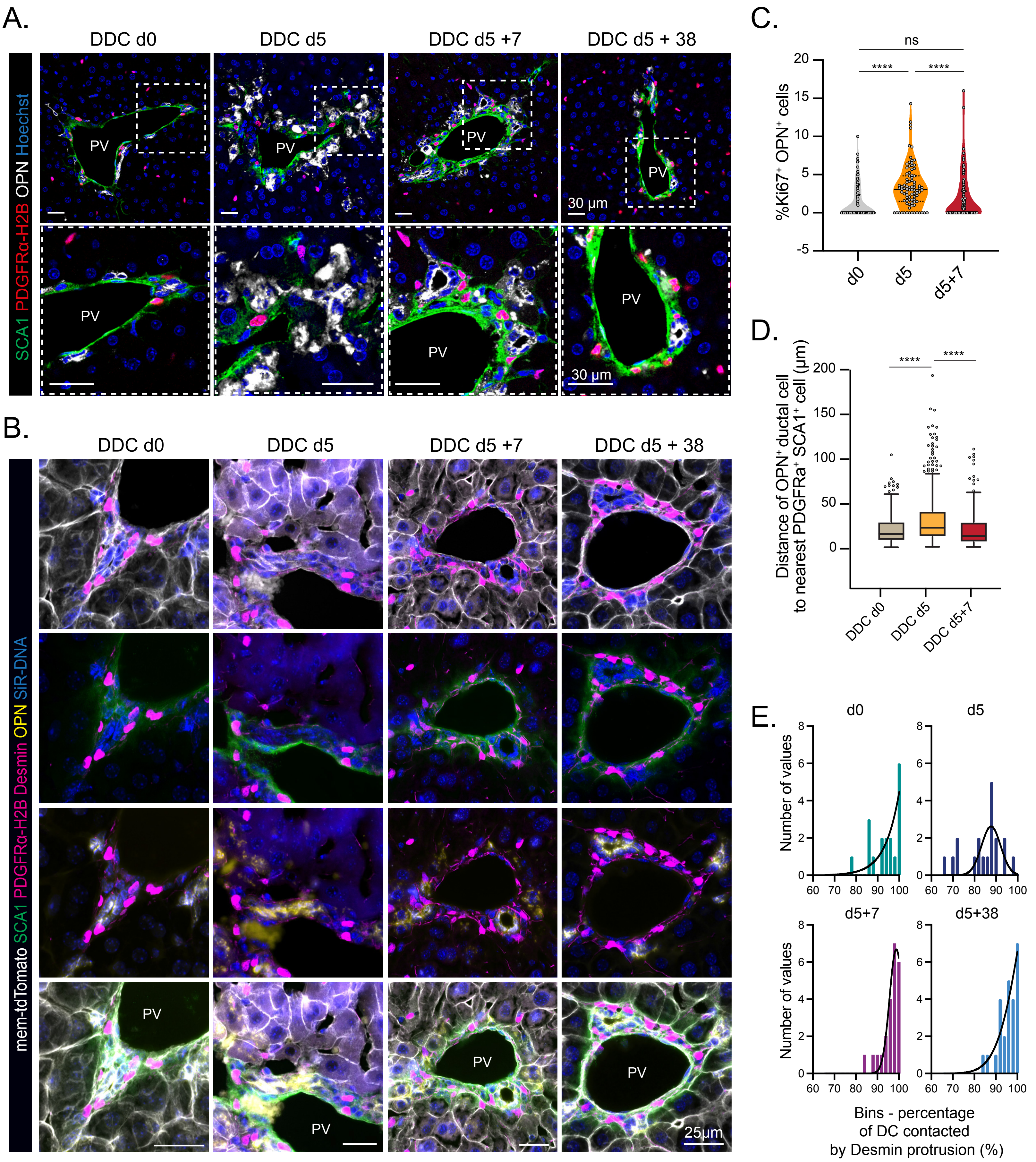

Figure S3. SCA1$^+$ mesenchyme in vivo during regeneration. Related to Figure 3.
A) Representative single z-stack images of livers from Pdgfra-H2B-GFP (nuclear red) mice damaged as above and stained for SCA1 (green) and OPN (white). Nuclei were counterstained with Hoechst (blue). PV, portal vein. Composite pictures were obtained by merging the single channel images in FIJI/ImageJ.
(B) Representative maximum intensity projection images of livers from Pdgfra-H2B-GFP/mTmG (nuclear magenta and membrane white) mice before (d0), during damage (d5) and and at different time points after damage (d5+7, d5+38) as detailed in methods. Sections were stained for desmin (magenta), SCA1 (green) and OPN (yellow). Nuclei were stained with SiR-DNA (blue). Composite pictures were obtained by merging the single channel images in FIJI/ImageJ.
C) Violin plot graph representing the data point distribution, median and IQR of the percentage of Ki67$^+$ OPN$^+$ ductal cells in undamaged (d0), d5 (damaged) and d5+7 (recovery) livers from n=3 independent experiments. p-values were obtained via Mann-Whitney t-tests. d0 vs d5 and d5 vs d5+7, p<0.0001 (****); d0 vs d5+7, p=0.1168 (ns).
D) Box and whiskers Tukey plot (median, whiskers are 1.5 interquartile range) represents the distance between OPN$^+$ DC and PDGFRα$^+$SCA1$^+$ Msc cells (see methods) in DDC-damaged livers at d0, d5 and d5+7 (n=2). Dots are outliers. P-values were obtained via Mann-Whitney tests. ****, p<0.0001.
(E) Frequency distribution and Gaussian curve of the percentages for contacts, presented in Figure 3B.

Cordero-Espinoza et al._Figure S4

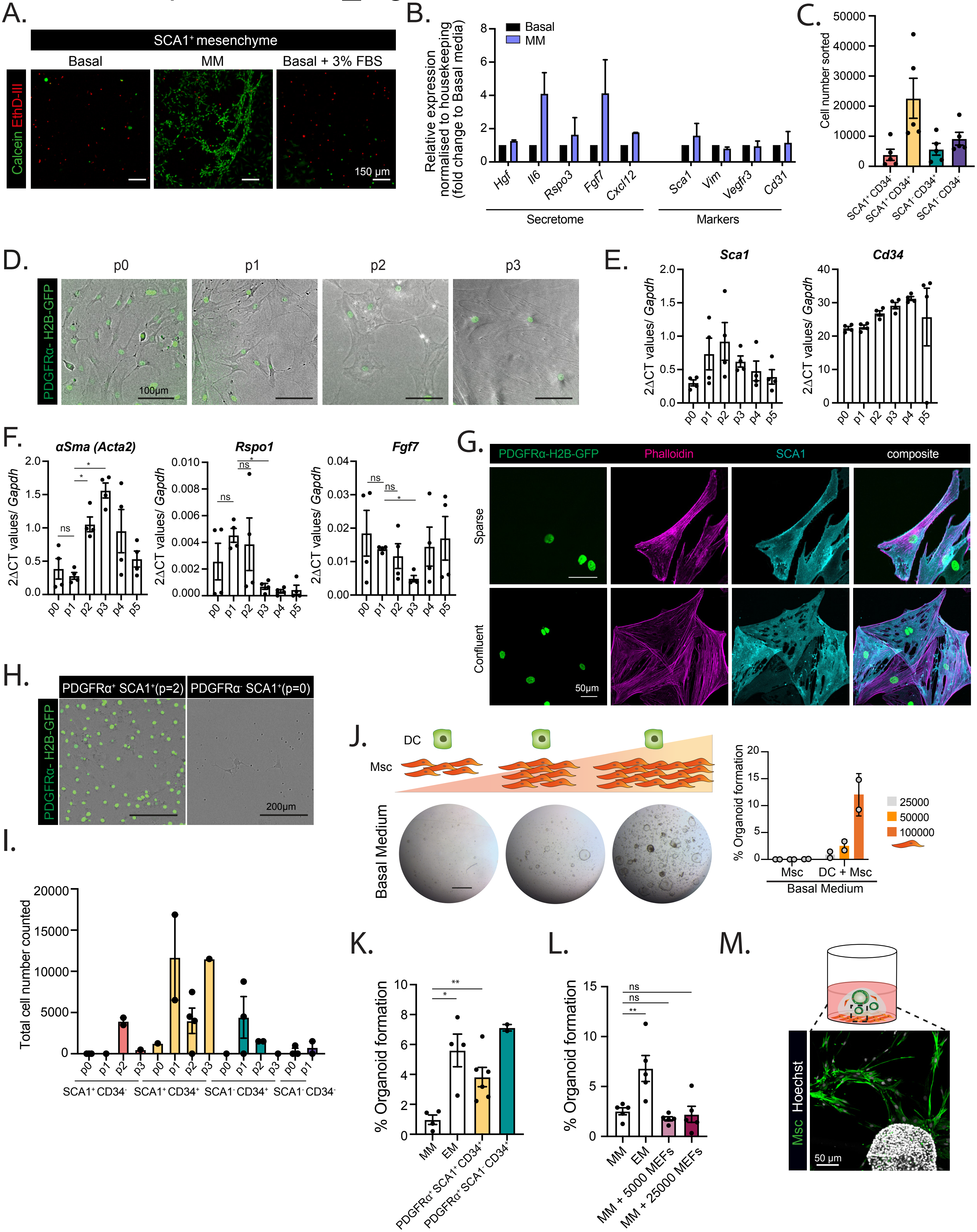

Figure S4. Growth and expansion of SCA1$^+$ mesenchymal cells in vitro. Related to Figure 4.

A) Cell viability assay indicates that SCA1$^+$ mesenchymal cells grow best in MM medium. SCA1$^+$ Msc cells were cultured within 3D Matrigel droplet in Basal (n=3), MM (n=4) and Basal + 3% FBS medium (n=3) and 6 days later cells were incubated with the cell viability dye calcein (4 μM, green) and the cell death dye EthD-III (8μM, red) and imaged using a fluorescence microscope. Composite pictures were obtained by merging the single channel images in FIJI/ImageJ.

B) Gene expression analysis of SCA1$^+$ Msc secretome and markers in presence or absence of 30% WNT 3a conditioned media in MM or Basal media supplemented with 3%FBS. Fold change normalized to basal media condition (n=2 biological and independent replicates).

C) Number of sorted cells from each isolation performed for the specified Msc subpopulations (PDGFRα$^+$SCA1$^+$CD34$^-$, red; PDGFRα$^+$SCA1$^+$CD34$^+$, yellow; PDGFRα$^+$SCA1$^-$CD34$^+$, green; PDGFRα$^+$SCA1$^-$CD34$^-$, purple)

D) Representative images of Msc cells in passage 0, passage1, passage 2 and passage 3 grown on plastic in MM showing brightfield and Pdgfra-H2BGFP expression (green). Composite pictures were obtained by merging the single channel images in FIJI/ImageJ.

E-F) mRNA expression of Sca1, Cd34, aSma, Rspo1 and Fgf7 by qRT-PCR analysis (n=4, from 2 independent experiments) of serially passaged SCA1$^+$ PDGFRα$^+$ cells. p-values were obtained using Mann-Whitney test, ns p> 0.1, * p=0.0286.

G) Representative images of Msc PDGFRα$^+$SCA1$^+$ cells in sparse and confluent culture conditions (passage 3), stained for marker SCA1 (cyan) and phalloidin (magenta) to visualize actin network. Nuclei from Pdgfra-H2B-GFP are also shown (green). Composite pictures were obtained by merging the single channel images in FIJI/ImageJ.

H) Brightfield and fluorescence images of PDGFRα$^+$SCA1$^+$ Msc following 2 serial passages (p2) of culture on plastic and with MM. Note that, PDGFRα$^-$SCA1$^+$ cells cannot be expanded under these culture conditions. Composite pictures were obtained by merging the single channel images in FIJI/ImageJ.

(I) Total cell number counted of the corresponding Msc populations counted at the time of passage after expansion.

(J) Organoid formation efficiency correlates with the number of mesenchymal cells in the co-culture. Increasing numbers of freshly sorted Msc cells were cultured alone or with EpCAM$^+$ DC in a 3D Matrigel droplet overlaid with medium w/o any growth factors (basal medium) and 10 days later organoid formation was assessed. Representative bright field images are shown. Scale bar, 100μm. Graph represents the mean ± SD of n=2 independent experiments.

K) Organoid formation efficiency assessed after 7 days of Matrigel droplet co-culture of 5000 freshly sorted ductal cells with 4000 mesenchymal cells of the specified fraction. p-values were obtained using Welch's test, p<0.01 (**), p<0.1 (*); n=5 for MM, EM and CD34$^+$ SCA1$^+$, and n=2 for CD34$^+$SCA1$^-$ cells.

L) Organoid formation efficiency assessed after 7 days of Matrigel droplet co-culture of 5000 freshly sorted ductal cells with 5000 or 25 000 MEF (mouse embryonic fibroblast) cells. P-values were obtained using Mann-Whitney test, p<0.01 (**), ns> 0.1.

(M) Representative image of an 8-day co-culture between ductal cells and mesenchymal cells (green) seeded within a 3D Matrigel droplet co-culture. Note that most of the mesenchymal cells attach to the bottom of the culture plate and spatially segregate from the DC-derived organoids, not establishing any cell-cell contact. Nuclei were counterstained with Hoechst (white). Composite pictures were obtained by merging the single channel images in FIJI/ImageJ.

Cordero-Espinoza et al._Figure S5

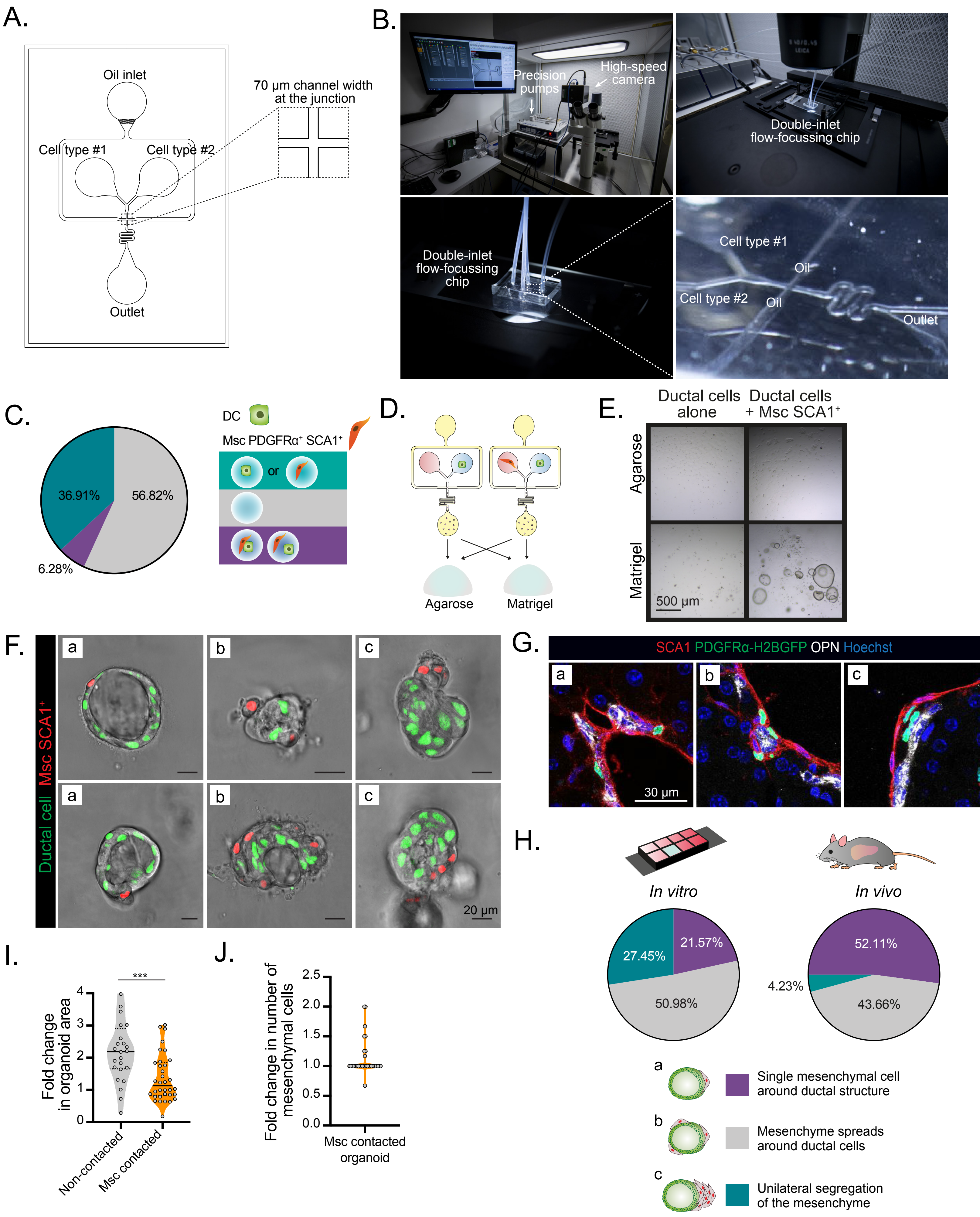

Figure S5. Msc-contacted organoids containing ductal and mesenchymal cells recapitulate in vitro the ductal : mesenchymal architecture of the portal tract. Related to Figure 5.

Organoid cells were encapsulated alone or with SCA1$^+$ Msc cells into agarose droplets using an FFD, seeded into 8-μ well dishes, imaged live for 24h at day 4 post-encapsulation and evaluated for the generation of organoids containing ductal and mesenchymal cells.

A) Design of the microfluidic double-inlet flow-focusing device (chip).

B) Pictures showing the microfluidic set up; precision pumps control the flow of liquids from syringes; high speed camera can monitor encapsulation in the double-inlet flow-focusing chip; close up of the chip; the whole apparatus is enclosed in a laminar flow hood.

C) Pie chart summarizing the frequency of microgels containing no cells (grey), only one cell-type (either DC or Msc PDGFRα$^+$SCA1$^+$ cells, teal), or both cell types (purple) at time t=0 following microfluidic encapsulation. n=5 independent experiments were performed.

D-E) Agarose microgels were seeded into 8-μ well dishes containing a 3D Matrigel or agarose layer and cultured in MM. D) Scheme of the experimental design. E) Representative brightfield images of organoid formation. Note that organoids were only generated when agarose microgels were embedded in Matrigel following a co-encapsulation with ductal and mesenchymal cells (n=3).

F) Representative single z-stack snapshots of Msc-contacted organoids at d4 post microfluidic encapsulation showing different ductal-mesenchymal cell dispositions categorized as a (1 mesenchymal cell attached), b (mesenchymal cells spread on the periphery of the organoid) or c (mesenchymal cells segregated to one side of the organoid). Composite pictures were obtained by merging the single channel images in FIJI/ImageJ.

G) Representative single z-stack images of homeostatic Pdgfra-H2B-GFP (nuclear green) mouse livers co-stained with SCA1 (red), Osteopontin (OPN, white) and counter-stained with Hoechst (blue). Categories as in F: a (1 mesenchymal cell around ductal epithelium), b (mesenchymal cells spread around the ductal epithelium) or c (mesenchymal cells segregated to one side of the ductal epithelium).

H) Pie chart summarizing the array of ductal-mesenchymal cell dispositions in vitro (left) and in vivo (right) from n=3 independent experiments (n=51 organoids and n=85 bile duct structures). Composite pictures were obtained by merging the single channel images in FIJI/ImageJ.

I) Violin plot graph representing the data point distribution, median and IQR of fold changes in organoid area in mesenchyme-contacted and non-contacted structures within a 24h-period of time-lapse imaging at d4 following microfluidic encapsulation. P-value was obtained by Mann-Whitney test. ***, p=0.0006, n=3 independent experiments.

J) Violin plot graph representing the data point distribution, median and IQR of the fold change of mesenchymal cell numbers in Msc-contacted organoids within a 24h-period of time-lapse imaging at d4 following microfluidic encapsulation, n=3 independent experiments.

Cordero-Espinoza et al._Figure S6

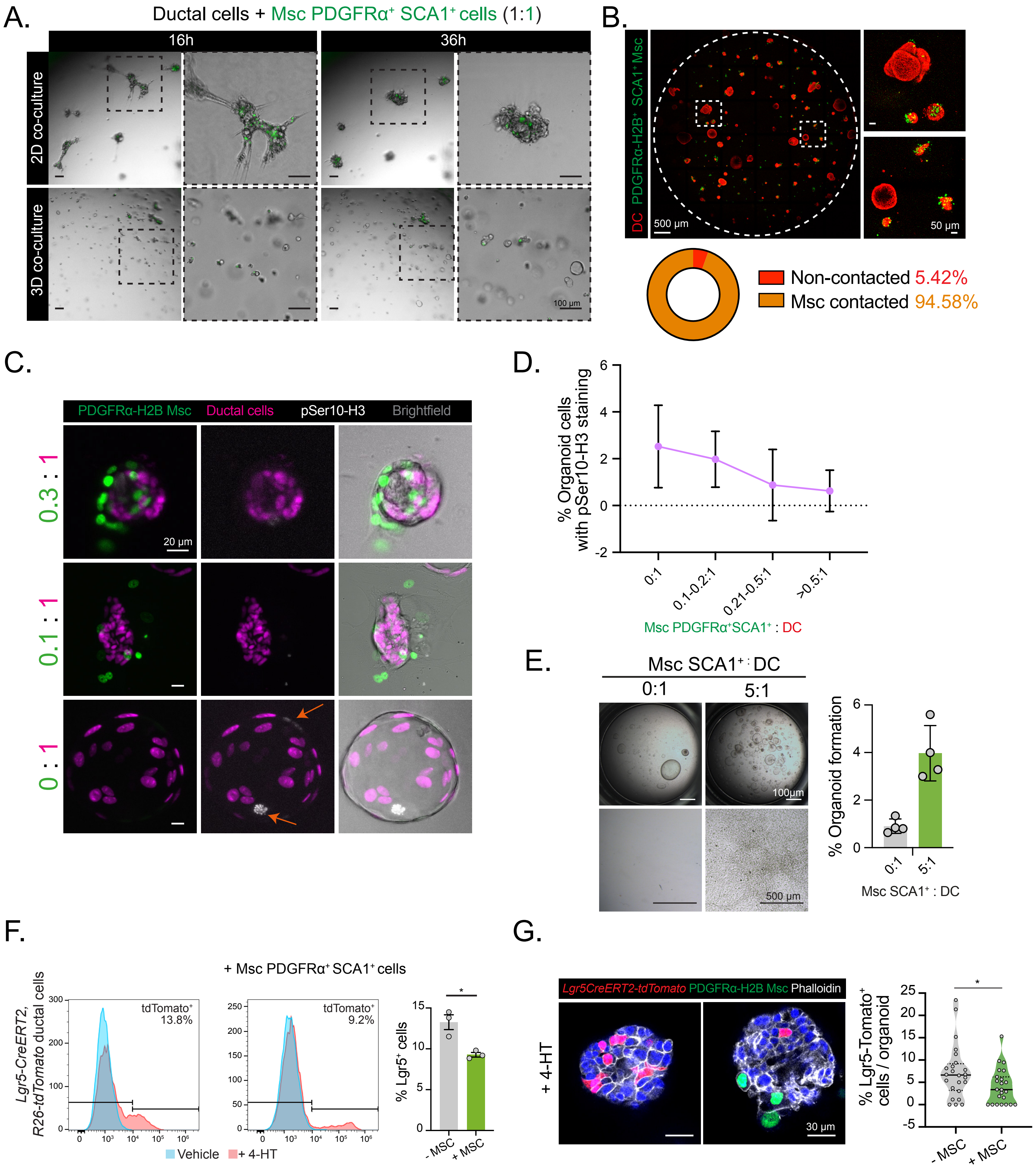

Figure S6. The ratio between ductal cells and mesenchymal cells in co-cultures determines the net outcome of ductal cell proliferation. Related to Figure 6.

A) Ductal cells (5,000 cells) from organoids were co-cultured with 5,000 PDGFRα-GFP$^+$ SCA1$^+$Msc (green, 1:1 ratio) in a 96-well plate by either culturing them on top of a well pre-coated with a Matrigel layer (top) or embedding them within a Matrigel droplet (bottom). Representative pictures at 16h and 36h after seeding are shown. Composite pictures were obtained by merging the single channel images in EVOS software.

B) Aggregation efficiency of nuclear tdTomato$^+$ DC (red) and PDGFRα-GFP$^+$ SCA1$^+$ Msc (green) seeded at a 1:1 ratio (5,000 cells each) on a Matrigel layer. Representative images of one of n=4 independent biological replicates are shown. Composite pictures were obtained by merging the single channel images in FIJI/ImageJ.

C-D) Mitoses decrease with increasing co-culture ratios of Msc to DCs. C) Msc-contacted (PDGFRα$^+$SCA1$^+$ Msc, nuclear green) and non-contacted organoids (nuclear magenta), stained for phospho-Serine10-Histone H3 (pSer10-H3, marker of mitosis, white); ratio of Msc to DC is specified for each structure; orange arrows point to dividing cells. Composite pictures were obtained by merging the single channel images in FIJI/ImageJ. D) Graph represents mean ± SEM of n=3 independent experiment quantification of detected pSer10-H3 staining in organoid structures has been divided into bins specified by the ratio of Msc to DC cells in each structure.

E) Freshly sorted EpCAM$^+$ DC were cultured in a transwell alone or with SCA1$^+$Msc at a 1:5 ratio for 8 days in MM. Note that in the absence of cell-cell contact, SCA1$^+$ mesenchymal cells do not inhibit ductal cell proliferation even at a >10-fold higher ratio (1:5) than the homeostatic ratio. Representative images of the top and bottom parts of the transwell are shown. Graph represents the quantification of organoid formation. Data is plotted as mean ± SD of n=3 independent experiments.

F) Single Lgr5CreERT2, R26-tdTomato ductal organoid cells were cultured alone or co-cultured with PDGFRα-GFP$^+$SCA1$^+$ Msc cells (1:1 ratio) on top of Matrigel and overlaid with EM + WNT3a CM medium. On day 3, cultures were incubated with 10μM of 4-hydroxytamoxifen (4-HT) and analyzed for percentage of tdTomato$^+$ organoid cells via flow cytometry 24h later. Graph represents the mean ± SD (n=3) of the number of Lgr5$^+$ cells quantified by FACS. Note that upon contact-permissive co-culture the number of Lgr5$^+$ cells is significantly reduced even in the presence of all growth factors and WNT3a ligand. P-value was obtained by Student t-test. *, p=0.0137.

G) PDGFRα-GFP$^+$ SCA1$^+$ Msc cells (geen) were co-cultured with single Lgr5CreERT2/R26-tdTomato liver organoid cells at a 1:0.5 ratio in complete EM + WNT3a CM medium in a 96-well plate. At day 3 of culture, cells were treated with 4-HT and fixed/stained 24h later. Representative single z-stack immunofluorescence images of same-well organoids counter-stained with Hoechst (blue) and Phalloidin (white). Violin plot graph represents the percentage of Lgr5-tdTomato$^+$ cells/organoid in Msc-Contacted vs non-contacted organoids. Note that in Msc-contacted organoids, where cell-cell contact is established, the number of Lgr5$^+$ cells is significantly reduced even in the presence of all growth factors and WNT3a ligand in the medium. P-value was obtained by Mann-Whitney test. *, p=0.0348 from n=23 organoids each from two independent experiments. Composite pictures were obtained by merging the single channel images in FIJI/ImageJ.

Cordero-Espinoza et al._Figure S7

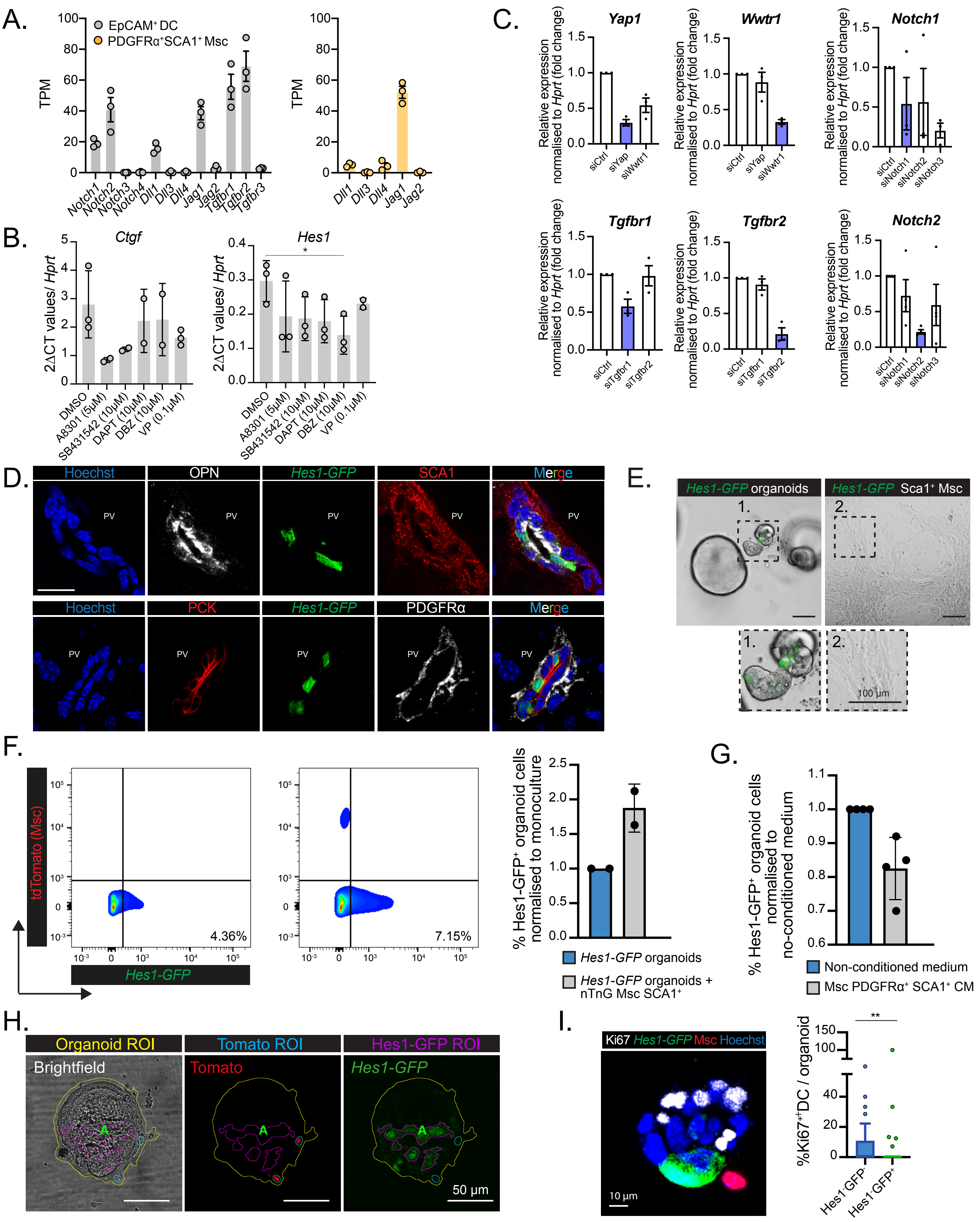

Figure S7. Cell-cell contact from PDGFRα$^+$SCA1$^+$ Msc inhibits DC proliferation via Notch signaling. Related to Figure 7.
A) Gene expression analysis on selected genes of the Notch and Tgfb pathway in freshly sorted EpCAM$^+$ DC (grey bars) and PDGFRα$^+$SCA1$^+$ Msc cells (orange bars) subject to RNA sequencing. Graphs represent mean ± SEM of n=3 mice.
B) Ductal organoid cells were treated with DMSO, A8301 (5μM), SB431542 (10μM), DAPT (10μM), DBZ (10μM) or Verteporfin (VP, 0.1μM) for 24h and mRNA expression of Ctgf and Hes1 was measured via RT-qPCR. Graph shows mean ± SD of n=3 independent experiments. P values were calculated with a Student t-test. All treatments were compared to DMSO control. For Ctgf expression: A8301, p=0.1132 (ns); SB431542, p=0.1713 (ns); DAPT, p=6207 (ns); DBZ, p=0.6621 (ns); VP, p=0.1688 (ns). For Hes1 expression: A8301, p=0.2086 (ns); SB431542, p=0.0953 (ns); DAPT, p=0.0799 (ns); DBZ, p=0.0285 (*); VP, p=0.2419 (ns).
C) Ductal organoid cells (50,000) were transfected with siRNAs oligos against the indicated genes and efficiency of knockdown was assessed 24h later by determining the expression of the corresponding genes via RT-qPCR. Graph represents the mean ± SEM of n=3 biological replicates.
D) Single z-stack images of Hes1-GFP mouse livers immunostained against OPN (white) and SCA1 (red) (top) or PCK (red) and PDGFRα (white) (bottom) and counterstained with Hoechst. Note that Hes1-GFP expression (green) is restricted to the ductal compartment (OPN$^+$ or PCK$^+$ cells). Composite pictures were obtained by merging the single channel images in FIJI/ImageJ.
E) Ductal cells (DC) and SCA1$^+$ Msc were isolated from Hes1-GFP mice and cultured in EM and MM respectively. Representative images of GFP fluorescence are shown (DC, n=4; SCA1+ Msc n=2). Composite pictures were obtained by merging the single channel images in EVOS software.
F) Single Hes1-GFP ductal organoid cells were cultured alone (left panel) or with nuclear tdTomato$^+$ SCA1$^+$ Msc cells at 1:1 ratio in growth factor rich medium (EM + WNT3a CM medium, right panel) and on top of a well coated with a layer of Matrigel. On day 8, the cultures were analyzed for Hes1-GFP expression via flow cytometry. Graph presents mean ± SD of the number of Hes1-GFP$^+$ organoid cells in co-culture vs monocultures. n=2 independent experiments.
G) Single Hes1-GFP ductal organoid cells were cultured in conditioned media from PDGFRα$^+$SCA1$^+$ Msc cells or non-conditioned media control (refreshed every 48h) for 8 days. On day 8, the cultures were analyzed for Hes1-GFP expression via flow cytometry. Graph presents Mean ± SD of the number of Hes1-GFP$^+$ cells (n=4).
H) Representative signal masks generated by a custom-made ImageJ script detecting organoid area (yellow outline), nuclear tdTomato fluorescence (cyan outline) and Hes1-GFP fluorescence (purple outline) in single z-stack images of Hes1-GFP organoid cells co-cultured with nuclear tdTomato$^+$ SCA1$^+$ Msc cells at 1:0.5 ratio (DC:Msc) in mesenchymal medium (MM).
I) Ki67 immunostaining (white) in 5-day Matrigel co-cultures between Hes1-GFP organoid cells (green) and nuclear tdTomato$^+$ SCA1$^+$ Msc cells (red, seeded at 1:0.5 DC:Msc) (left). Quantification of the percentage of Ki67$^+$ cells in Hes1-GFP$^-$ vs Hes1-GFP$^+$ ductal cells per organoid. Tukey box plot displaying the median and IQR (n=3). p=0.0076, Mann Whitney test (right). Composite pictures were obtained by merging the single channel images in FIJI/ImageJ.
