# Supplementary material for: Dynamic cell contacts between periportal mesenchyme and ductal epithelium act as a rheostat for liver cell proliferation
Source: Cell Stem Cell. 2021 Nov 4;28(11):1907–1921.e8. doi: 10.1016/j.stem.2021.07.002 (PMC8577825; doi:10.1016/j.stem.2021.07.002)
Supplement: Document S2 — Article plus supplemental information [file mmc9.pdf]

Cell Stem Cell

Article

Dynamic cell contacts between periportal mesenchyme and ductal epithelium act as a rheostat for liver cell proliferation

Graphical abstract

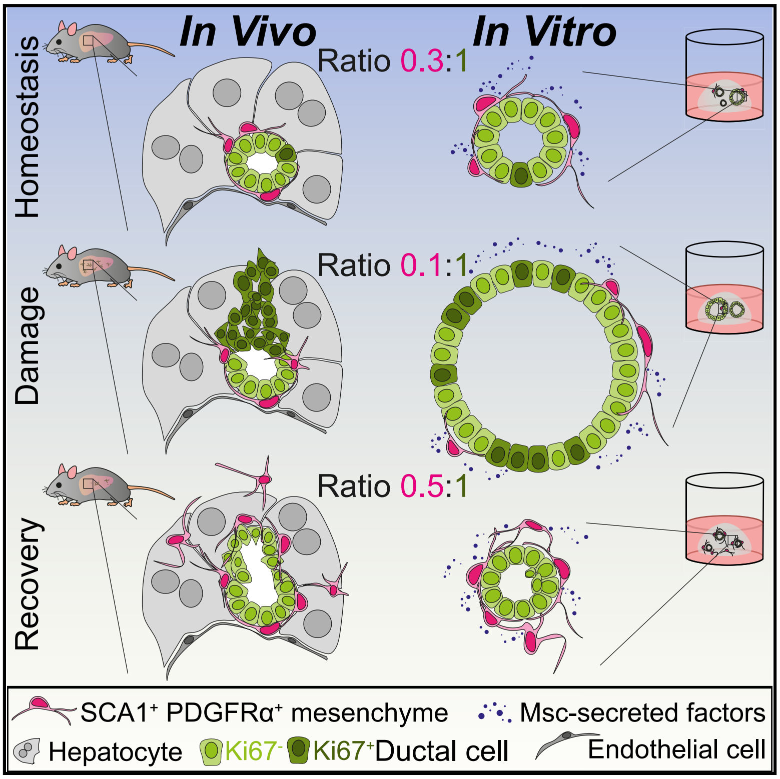

Highlights

- The numbers of mesenchyme-ductal cell contacts change during damage regeneration
- Ductal-mesenchyme organoids recapitulate cell interactions and *in vivo* architecture
- Mesenchymal cells induce ductal epithelium cell proliferation via soluble factors
- Mesenchymal cells impair ductal epithelium cell proliferation via cell-cell contacts

Authors

Lucía Cordero-Espinoza, Anna M. Dowbaj, Timo N. Kohler, ..., Neil C. Henderson, Florian Hollfelder, Meritxell Huch

Correspondence

huch@mpi-cbg.de

In brief

Huch and colleagues describe that during liver regeneration, ductal and mesenchymal cells proliferate with different tempos, resulting in changes in the cellular contacts between both populations. By developing organoid co-cultures that recapitulate ductal-mesenchymal interactions, they provide proof that the number of cell contacts matters and either promotes or arrests epithelial proliferation.

Cordero-Espinoza et al., 2021, Cell Stem Cell *28*, 1907–1921
November 4, 2021 © 2021 The Authors. Published by Elsevier Inc.
https://doi.org/10.1016/j.stem.2021.07.002

Cell Stem Cell

CellPress
OPEN ACCESS

Article

Dynamic cell contacts between periportal mesenchyme and ductal epithelium act as a rheostat for liver cell proliferation

Lucía Cordero-Espinoza,[1,2,3,11] Anna M. Dowbaj,[4,11] Timo N. Kohler,[2,5] Bernhard Strauss,[1] Olga Sarlidou,[1] German Belenguer,[4] Clare Pacini,[6] Nuno P. Martins,[4] Ross Dobie,[7] John R. Wilson-Kanamori,[7] Richard Butler,[1] Nicole Prior,[4,10] Palle Serup,[9] Florian Jug,[4] Neil C. Henderson,[7,8] Florian Hollfelder,[5] and Meritxell Huch[1,2,3,4,12,*]

[1]Wellcome Trust/Cancer Research UK Gurdon Institute, Cambridge CB2 1QN, UK
[2]Wellcome Trust–Medical Research Council Stem Cell Institute, Cambridge CB2 1QR, UK
[3]Department of Physiology, Development and Neuroscience, University of Cambridge, Cambridge CB2 3DY, UK
[4]Max Planck Institute of Molecular Cell Biology and Genetics, Dresden 01307, Germany
[5]Department of Biochemistry, University of Cambridge, Cambridge CB2 1GA, UK
[6]Wellcome Sanger Institute, Wellcome Genome Campus, Hinxton, Cambridge CB10 1SA, UK
[7]Centre for Inflammation Research, The Queen's Medical Research Institute, University of Edinburgh, Edinburgh EH16 4TJ, UK
[8]MRC Human Genetics Unit, Institute of Genetics and Cancer, University of Edinburgh, Edinburgh EH4 2XU, UK
[9]Novo Nordisk Foundation Center for Stem Cell Biology (DanStem), University of Copenhagen, Copenhagen 2200, Denmark
[10]Present address: School of Biological Sciences, Highfield Campus, University of Southampton, Southampton SO17 1BJ, UK
[11]These authors contributed equally
[12]Lead contact
*Correspondence: huch@mpi-cbg.de
https://doi.org/10.1016/j.stem.2021.07.002

SUMMARY

In the liver, ductal cells rarely proliferate during homeostasis but do so transiently after tissue injury. These cells can be expanded as organoids that recapitulate several of the cell-autonomous mechanisms of regeneration but lack the stromal interactions of the native tissue. Here, using organoid co-cultures that recapitulate the ductal-to-mesenchymal cell architecture of the portal tract, we demonstrate that a subpopulation of mouse periportal mesenchymal cells exerts dual control on proliferation of the epithelium. Ductal cell proliferation is either induced and sustained or, conversely, completely abolished, depending on the number of direct mesenchymal cell contacts, through a mechanism mediated, at least in part, by Notch signaling. Our findings expand the concept of the cellular niche in epithelial tissues, whereby not only soluble factors but also cell-cell contacts are the key regulatory cues involved in the control of cellular behaviors, suggesting a critical role for cell-cell contacts during regeneration.

INTRODUCTION

The adult liver epithelium comprises hepatocytes and biliary ducts lined by liver ductal cells (DCs, also known as cholangiocytes). The epithelium is mostly mitotically dormant in homeostasis yet proliferates swiftly upon damage, enabling rapid regeneration (Miyajima et al., 2014). Although hepatocytes comprise the bulk of the regenerative response (Malato et al., 2011), DCs also respond to injury (Furuyama et al., 2011). In addition, severe tissue damage and hepatocyte senescence inducecellular plasticity in the ductal compartment and endow the otherwise unipotent cholangiocytes with the capacity to replace lost hepatocyte mass (Choi et al., 2014; Raven et al., 2017). Healthy adult DCs can be expanded *in vitro* as self-renewing liver organoids in a 3D extracellular matrix (Matrigel) and a defined cocktail of growth factors (R-Spondin-1 [RSPO1], Fibroblast Growth Factor 10 [FGF10], epidermal growth factor [EGF], and Hepatocyte Growth Factor [HGF]; Huch et al., 2013, 2015) that recapitulate the transient mitogenic milieu of the regenerating liver (Apte et al., 2008). Using this model system, we have shown that liver ductal organoids recapitulate many aspects of liver regeneration in a dish (Aloia et al., 2019). Notwithstanding, across multiple mammalian tissues, regeneration relies on the dynamic crosstalk between the epithelium and its respective tissue microenvironment (Gurtner et al., 2008). The contribution of the latter in the ductal-mediated regeneration of the liver are largely unknown.

The patterning of hepatic epithelium throughout development is dependent on cues from apposed mesenchymal tissues (Zaret, 2002). In the adult liver, the hepatic mesenchymal pool, whose ontogeny traces back to the septum transversum mesenchyme [STM] (Zorn, 2008), diversifies into centro-lobular fibroblasts and smooth muscle cells, lobule-interspersed hepatic stellate cells (HSCs), and a portal tract (PT)-restricted population referred to as portal fibroblasts (PFs) (Lepreux and Desmoulière, 2015). The physiology of these cells has been appraised in the context of various disease states (Mederacke et al., 2013;

This is an open access article under the CC BY license (http://creativecommons.org/licenses/by/4.0/).

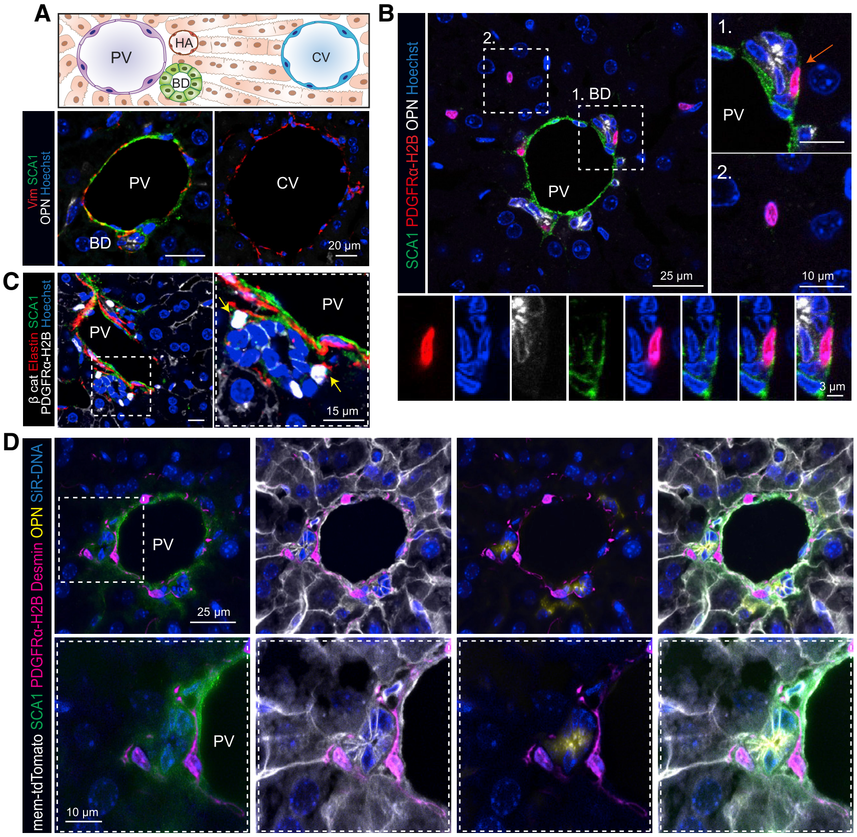

Figure 1. PDGFRα⁺SCA1⁺ mesenchymal cells reside near the portal tract

(A) SCA1 marks exclusively the portal tract region of the liver lobule. Top: schematic of a liver lobule, spanning from the portal tract (formed by the portal vein [PV], hepatic artery [HA], and bile duct [BD]) to the central vein (CV) area. Bottom: representative composite single z stack images of liver sections, stained for SCA1 (green), Vimentin (Vim, red), the ductal marker osteopontin (OPN, white) and nuclei (Hoechst, blue).

(B) Representative composite single z stack images of *Pdgfra-H2B-GFP* (nuclear red) mouse livers co-stained with SCA1 (green) and the ductal marker osteopontin (OPN, white). PDGFRα^{+}SCA1^{+} cells (B1, close up underneath) are in close proximity to the ductal epithelium (orange arrow), while PDGFRα^{+}SCA1^{-} cells (B2) are spread throughout the parenchyma.

(C) Representative composite single z stack images of *Pdgfra-H2B-GFP* (nuclear white) mouse livers co-stained with SCA1 (green), elastin (red), and β-catenin (white, membrane). Yellow arrow, PDGFRα^{+}SCA1^{+}.

(D) Representative composite maximum intensity projection image of PDGFRα^{+}SCA1^{+} cells (nuclear magenta) contacting liver ductal cells (OPN, yellow) through desmin (magenta) membrane protrusions; SCA1 staining (green), membrane marker tdTomato (white), and DNA (SiR-DNA, blue).

See also Figure S1 and Video S1.

Ramachandran et al., 2019). *In vivo* inhibition of HSC activation exacerbates liver damage while diminishing DC expansion (Pintilie et al., 2010; Shen et al., 2011). Similarly, Thy1^{+} HSCs and PFs have been identified as a source of Fibroblast Growth Factor 7 [FGF7] that sustains DC proliferation during regeneration (Takase et al., 2013), while Jagged1^{+} myofibroblasts direct ductal lineage differentiation in mouse models of chronic liver damage (Boulter et al., 2012). Although these studies highlight discrete cases of mesenchymal-to-ductal cell signaling, they do not address the dynamic interactions between these lineages in homeostasis or throughout the different phases of the regenerative response. *In vitro* co-culture models with different stromal populations have been devised to enhance the stability and functions of hepatocytes (Berger et al., 2015; Bhatia et al., 1998; Coll et al., 2018; Davidson et al., 2017; Nguyen et al., 2015; Ouchi et al., 2019; Taymour et al., 2021; Ware et al., 2017). However, ductal-mesenchymal co-cultures have not been reported yet. In addition, current *ex vivo* adult liver organoid models are epithelial centric and fail to recapitulate the multicellular complexity of the adult tissue (Prior et al., 2019), hampering an in-depth understanding of the stromal niche-to-epithelial cell interactions during homeostasis and regeneration.

Here, we describe that a subpopulation of peri-portal mesenchymal cells (labeled by platelet-derived growth factor receptor alpha [PDGFRα] and stem cell antigen 1 [SCA1]) acts as a rheostat that regulates the proliferation capacity of DCs. Mesenchymal-secreted mitogens support organoid formation and expansion. However, direct mesenchymal-to-ductal cell contact abolishes DC proliferation in a mesenchyme-dose-dependent manner through mechanisms that involve, at least in part, Notch signaling activation. Hence, our results indicate that the number of cellular contacts between epithelium and mesenchyme, rather than the absolute number of cells in both populations, controls DC proliferation dynamics.

RESULTS

Periportal PDGFRα^{+}SCA1^{+} mesenchymal cells surrounding the duct epithelium express a pro-regenerative growth factor signature

The process of tissue regeneration is a joint endeavor between the epithelium and surrounding stroma. Accordingly, we first sought to characterize the proximate neighbors of the ductal epithelium, which we hypothesized could act as a regulatory niche for DC-driven regeneration. Liver ductal cells (DCs; also known as cholangiocytes) reside at the PT area of the liver lobule (Figure 1A), spatially separated from the mid-lobular and central vein (CV) zones. We found that the hematopoietic and cancer stem cell and surface marker SCA1 (encoded by the gene *Ly6a*; Upadhyay, 2019) labeled cells exclusively localized at the PT, in proximity to and including the biliary ductal epithelium, as identified by osteopontin (OPN). In contrast, SCA1 expression was absent or below detection limit in the remainder of the liver parenchyma (Figures 1A, 1B, S1A, and S1B). SCA1 expression was also detected in the CD31^{+} endothelium lining the portal vein, but not the VEGFR3^{+} sinusoidal

endothelial network or liver-resident macrophages (F4/80$^+$) (Figures S1A and S1B).

To determine whether SCA1$^+$CD31$^-$OPN$^-$ cells encompassed a population of periportal mesenchyme, we analyzed the expression of SCA1 in livers derived from *Pdgfra-H2B-GFP* mice, which readily report expression of the archetypal mesenchymal marker PDGFRα (Figure S1C). We found that SCA1 labels a subpopulation of mesenchymal cells that surround and directly contact the ductal epithelium (Figures 1B–1D and S1D–S1G; Video S1) and express mesenchymal markers such as CD34, elastin, desmin, and reelin (Figures 1C, 1D, and S1D–S1F). PDGFRα^+SCA1$^+$ cells were located at a median distance of 8 μm from the center of the biliary duct, whereas this distance was more than tripled for the PDGFRα^+SCA1$^-$ fraction (Figure S1H). Accordingly, we utilized PDGFRα^+SCA1$^+$ expression as a proxy for identifying the mesenchymal cells nearest to the biliary epithelium and focused on this population from here onward. Pericytes (αSMA$^+$) appeared to be distinct from the PDGFRα^+SCA1$^+$ mesenchyme (Figure S1G).

For an in-depth analysis of the peri-ductal associated stroma, we isolated PDGFRα^+SCA1$^+$ as well as PDGFRα^-SCA1$^+$, PDGFRα^+SCA1$^-$, and EpCAM$^+$ DCs from healthy murine livers and obtained their transcriptional profile (Figures 2A and 2B). Transcriptome and qRT-PCR analysis confirmed that the PDGFRα^+SCA1$^+$ and PDGFRα^+SCA1$^-$ cells expressed a clear mesenchymal gene signature, including markers such as *Pdgfra*, *Pdgfrb*, *Eln*, and *Cd34* and various collagens (*Col1a1* and *Col1a2*). In contrast, endothelial (e.g., *Kdr*) genes were highly expressed in the PDGFRα^-SCA1$^+$ fraction, while ductal (e.g., *Krt19*)-specific genes were highly expressed in the EpCAM$^+$ fraction, as expected. Endothelial and DC markers were weakly expressed or absent in the PDGFRα^+SCA1$^+$ cells (Figures 2C, 2D, S2A, and Data S1), while some mitogens such as *Rspo1* and *Hgf* were expressed (Figure S2B and Data S1). Moreover, Thy1 (Katsumata et al., 2017) and *Lrat* (Mederacke et al., 2013), purported PF and HSC markers, respectively, were also expressed in both PDGFRα^+SCA1$^+$ and SCA1$^-$ populations (Figures 2C and 2D) while reelin (*Reln*), a well-known HSC marker, was mainly present in the PDGFRα^+SCA1$^-$ fraction (Figures 2C and 2D), in agreement with our immunostaining analysis (Figure S1E). These results thereby suggested a high degree of mesenchymal cell heterogeneity.

To gain deeper insight into this heterogeneity and increase the resolution of the SCA1$^+$ mesenchymal cell expression profile, we utilized our published single-cell RNA sequencing (scRNA-seq) data of murine liver mesenchyme (Dobie et al., 2019), where three distinct mesenchymal cell clusters are readily identified (Figure 2E): an *Acta2*-enriched vascular smooth muscle cell (VSMC) population, an HSC cluster marked by *Lrat*- and *Reelin*-positive cells, and a PF cluster marked by *Cd34*-expressing cells (Figure S2C). High *Ly6a* (SCA1)$^+$ cells were identified in the PF cluster, while cells expressing weaker levels were detectable within the HSC fraction (Figure 2E). Notably, both HSCs and PFs expressed various mitogens and growth factors, including *Rspo1*, *Rspo3*, *Fgf7*, and *Hgf* (Figures 2F and S2D), all essential for duct and hepatocyte specification (Rossi et al., 2001), regeneration (Hu et al., 2007; Kan et al., 2009; Yang et al., 2014), and organoid formation (Huch et al., 2013, 2015). These results were confirmed in sorted PDGFRα^+SCA1$^+$ cells (Figures 2G and S2B) as well as in PDGFRα^+SCA1$^+$ cells after sub-fractioning into PFs and HSCs using the bona-fide PF marker CD34 (PFs, CD34$^+$; HSCs, CD34$^-$) (Figures S2E and S2F).

Collectively, these results suggested that PDGFRα^+SCA1$^+$ mesenchymal cells (hereafter SCA1$^+$Msc cells) represent a periportal, duct-contacting, mesenchymal subpopulation that expresses markers of both PFs and HSCs and is enriched in paracrine mitogens capable of modulating DC expansion.

The cellular ratios and cell contacts between DC and SCA1$^+$ mesenchyme change dynamically during the damage-repair response and negatively correlate with DC proliferation

In vivo, the damaged-induced proliferation of DCs is facultative and arrests once the tissue is regenerated, thus warranting the return to homeostasis (Cordero-Espinoza and Huch, 2018). Having observed that SCA1$^+$Msc cells express mitogens known to regulate DC expansion during regeneration and organoid formation, we hypothesized that the relative abundance and contact between both populations could dictate the proliferative state of the ductal epithelium.

To test this hypothesis, we modeled acute liver damage by feeding mice with 0.1% 3,5-diethoxycarbonyl-1,4- dihydrocollidine (DDC) for 5 days, followed by a recovery period in normal diet for 7 and 38 days (Figures 3A, S3A, and S3B), and quantified total cell numbers, cell ratios, and cell contacts between DCs and SCA1$^+$Msc (Figures 3B–3D). In healthy tissue, DCs display limited proliferation (Figure S3C). SCA1$^+$Msc and DC (OPN$^+$) co-exist periportally within close proximity ($\sim$11 μm) (Figures 3A, S3A, S3B, and S3D), with a median population ratio of 0.3 Msc cells per 1 DC (0.3:1, from hereon) (Figure 3C) and the majority of DCs ($\sim$93%) contacted by a SCA1$^+$Msc cell (Figures 3A, 3D, and S3E). Following tissue damage (DDC day 5), DCs, but not PDGFRα^+ (SCA1$^+$ or SCA1$^-$) cells, increase in number (Figures 3B and S3C). This results in a significant drop in the PDGFRα^+SCA1$^+$/DC cell ratio (from 0.3:1 to 0.1:1) (Figure 3C), increased distance between both compartments (from $\sim$11 μm to $\sim$30 μm), and a significant decrease in the number of cell contacts between both populations (from $\sim$93% to $\sim$84%) (Figures 3D and S3E). By the early phase of recovery (DDC day 7, after recovery), the DC pool was still enlarged (Figure 3B), but the percentage of proliferating cells had diminished significantly to its pre-damage condition (Figure S3C). This coincided with an increase in the absolute number of SCA1$^+$Msc cells (Figure 3B), a raise in the cell ratios from 0.1:1 to 0.5:1 (Figure 3C), a return to the baseline distance (Figures 3A, S3A, S3B, and S3D), and a reestablishment of the cell contacts (from $\sim$84% to 96%) (Figures 3A and 3D). At day 38 of recovery (termination phase), both DCs and SCA1$^+$Msc cells returned to their steady-state numbers, ratios, spatial disposition, and cell contacts (Figures 3A–3D, S3A, S3B, and S3E). Of note, the PDGFRα^+SCA1$^-$ mesenchymal compartment did not increase its numbers during the entire damage-regenerative response (Figure 3B).

Taken together, these results indicate that both cell populations proliferate, but at different tempo; the DCs proliferate first, during damage (day 5), followed by the mesenchymal cells after the damage is removed (day 7, after recovery). This asynchrony results in a significant and dynamic change in the distance, cellular ratios, and number of cell contacts between both populations.

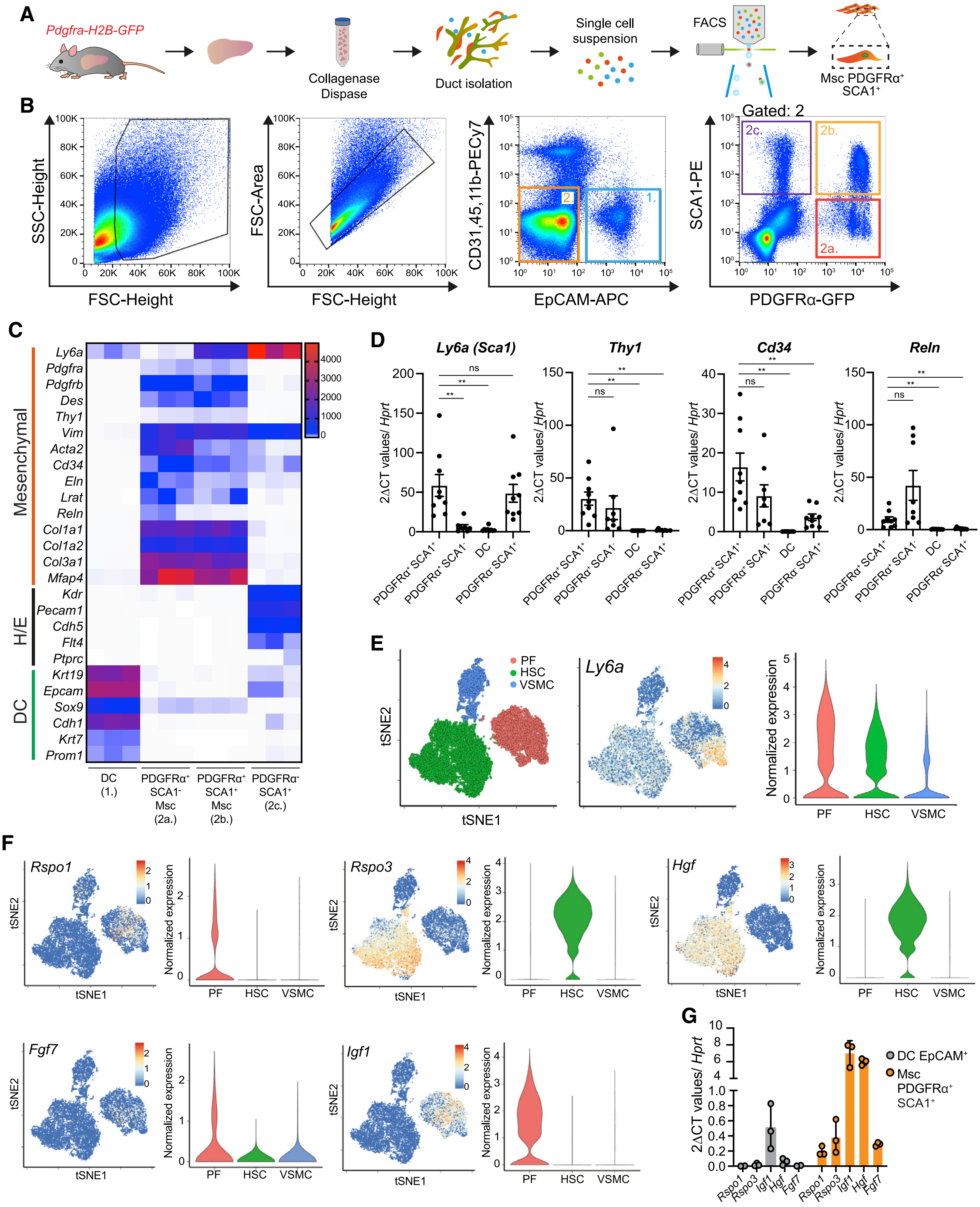

Figure 2. Periportal SCA1⁺Msc cells express a pro-regenerative growth factor signature

(A and B) Isolation of EpCAM⁺ ductal cells (DCs; gate 1), PDGFRα⁺SCA1⁻ (gate 2a), and PDGFRα⁺SCA1⁺ (gate 2b) Msc and PDGFRα⁻SCA1⁺ (gate 2c) stromal cells from *Pdgfra-H2B-GFP* mouse livers. (A) Experimental design. (B) Representative FACS plots.

(C) RNA sequencing (RNA-seq) analysis of the populations in (B). Heatmap represents the TPM (transcripts per million) values from the RNA-seq for the indicated genes (n = 3 biological replicates). H/E, hematopoietic/endothelial cell markers.

(D) RT-qPCR expression analysis of selected genes from freshly sorted DCs and specified niche cells in (B). Graph represents mean ± SEM of n > 8 biological replicates (mice) from n = 3 independent experiments. Unpaired t test with Welsch correction (**$p < 0.01$; ns, $p > 0.01$).

(E and F) scRNA-seq analysis of mouse hepatic Msc populations reported in Dobie et al. (2019). tSNE [t-distributed stochastic neighbor embedding] (left) and violin plots (right) indicating the *mRNA* expression levels for SCA1 (*Ly6a*; E) or the indicated growth factors (F). PF, portal fibroblast; HSC, hepatic stellate cell; VSMC, vascular smooth muscle cell.

(G) Gene expression analysis of selected secreted growth factor genes in the indicated sorted populations. Graph represents mean ± SD of n = 3 biological replicates (mice).

See also Figure S2.

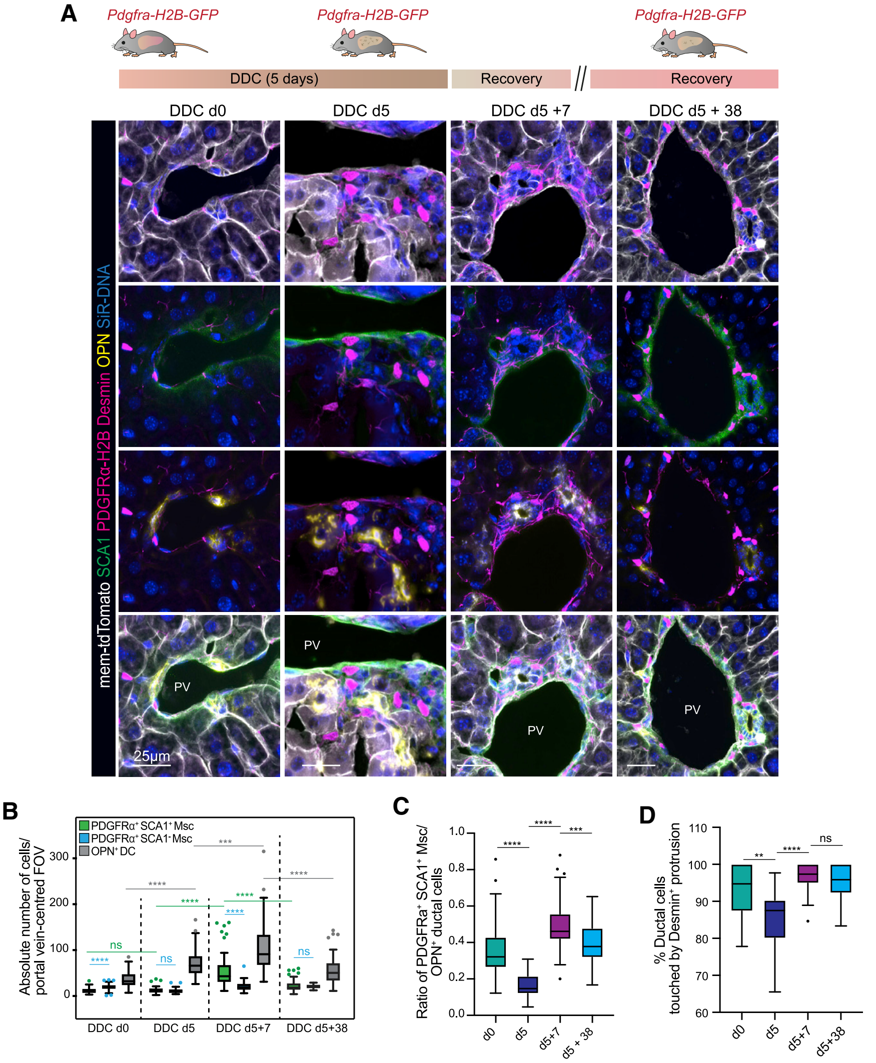

Figure 3. Relative abundance and cell contacts between DCs and PDGFRα$^+$SCA1^{+-} Msc cells change dynamically during the damage-regenerative response

(A–D) The number, distribution, and cell contacts between DCs, SCA1$^+$Msc cells, and SCA1$^-$ Msc cells were quantified before and after inducing liver injury and at days 7 and 38 of recovery.

(A) Top: scheme of experimental approach. Bottom: representative maximum intensity projection composite images of livers from *Pdgfra-H2B-GFP/mTmG* mice stained for desmin (magenta), SCA1 (green), and OPN (yellow), *Pdgfra-H2B-GFP* (nuclear; magenta), membrane tdTomato (white), and nuclei (SiR-DNA, blue).

(B) Box-and-whisker Tukey plot (median, whiskers are 1.5 interquartile range) of the absolute number of mesenchymal (PDGFRα$^+$SCA1$^+$ and PDGFRα$^+$ SCA1$^-$) and ductal (OPN$^+$) cells per field of view (FOV) of PV-centered composite confocal images from DDC-damaged livers at day 0 (n = 3), day 5 (n = 3), day 5 plus 7 days recovery (n = 3), and day 5 plus 38 days recovery (n = 2). Dots represent outliers. Mann-Whitney tests; ****$p < 0.0001$; ***$p < 0.001$; ns, $p > 0.05$.

(C) Box-and-whisker Tukey plot (median, whiskers are 1.5 interquartile range) of the ratio of the number of PDGFRα$^+$SCA1$^+$Msc cells relative to DCs. Dots, outliers. Mann-Whitney t tests; ****$p < 0.0001$; ***$p < 0.001$.

(D) Box-and-whisker Tukey plot (median, whiskers are 1.5 interquartile range) represents the percentage of DCs contacted by a desmin protrusion in DDC-damaged livers (n $\geq$ 3 mice). Dots, outliers. Mann-Whitney test; **$p < 0.001$; ****$p < 0.0001$.

See also Figure S3.

Accordingly, we can hypothesize a scenario whereby in steady state, SCA1$^+$Msc cells hold the ductal epithelium in a nonproliferative state; in contrast, upon damage, DC proliferation is prioritized, presumably by mitogenic signals, which results in a temporary drop in the steady-state ratios and cell contacts. Upon cessation of damage, mesenchymal cells expand and reestablish physical contacts with the ductal epithelium, which eventually reinstates the homeostatic, nonproliferative, steady state.

Mesenchyme-secreted factors activate DC proliferation and organoid formation

To test the hypothesis that the relative abundance and cell contacts between the two populations could control the proliferative state of the DCs during regeneration, we studied how dynamic changes in Msc/DC numbers and cell contacts impact epithelial cell behavior. For that, we opted to manipulate the cellular ratios between both populations *in vitro*, in organoid co-cultures, where experimental conditions can be controlled.

Like the regenerating tissue, organoids also depend on key growth factors that mimic the mitogenic microenvironment of the damaged liver. Considering that the SCA1$^+$Msc population expressed a battery of mitogens (Figures 2D–2G, S2B, and S2D) required for liver organoids to expand (Huch et al., 2013), we first sought to determine whether these cells would support the growth of liver ductal organoids *in vitro*. For that, we first identified culture conditions that would enable the maintenance of these mesenchymal cells *in vitro*. We selected AdDMEM/F12 supplemented with FBS and WNT3A (hereafter called mesenchymal medium [MM]) and culturing on plastic to enhance mesenchymal cell viability (Figures S4A and S4B). Given the low yield of primary PDGFRα$^+$SCA1$^+$ and PDGFRα$^+$SCA1$^-$ mesenchymal cells isolated from murine livers (Figure S4C), we investigated if our optimized culture conditions would enable expansion of these cells prior to co-culture. We readily expanded PDGFRα$^+$SCA1$^+$ cells (~2 months in culture, passage 5) (Figures S4D–S4G), while the endothelial PDGFRα$^-$SCA1$^+$ cells could not be consistently grown (Figure S4H). Notably, sub-fractioning the PDGFRα$^+$SCA1$^+$ cells into HSCs and PFs using CD34 (PFs, CD34$^+$ and HSCs, CD34$^-$), indicated that the cells that expanded in culture were mainly PFs (CD34$^+$) (Figure S4I). Similarly, from the PDGFRα$^+$SCA1$^-$ fraction, only PF (CD34$^+$) cells could be expanded, but at much lower efficiency (Figure S4I). Expanded PDGFRα$^+$SCA1$^+$ cells conserved their morphology,

secretome, and marker expression for the first three passages in culture (Figures S4D–S4G) but at passages 2–3 became activated (α*Sma*$^+$) (Rockey et al., 1992) and lost *Rspo1* expression (Figure S4F).

Next, we co-isolated SCA1$^+$Msc and EpCAM$^+$ DCs and embedded them together inside of 3D Matrigel droplets that were overlaid with MM medium (devoid of growth factors). Interestingly, we observed that co-cultures with SCA1$^+$Msc cells sustained organoid formation at an efficiency close to 4%, which was comparable to controls receiving the media supplemented with all growth factors and 4-fold higher than DC alone (Figure 4A). This effect was independent of WNT3A and FBS in the MM medium, since similar results were obtained when basal medium devoid of these components was used (Figure S4J). Culturing SCA1$^+$Msc cells on their own did not generate organoids, as expected (Figure 4A, Msc alone panel). Notably, sub-fractioning for CD34$^+$ PFs indicated that both SCA1$^+$ and SCA1$^-$ PFs were able to support organoid growth when expanded (Figure S4K), in contrast to control mouse embryonic fibroblasts (MEFs) (Figure S4L).

However, considering (1) the low yield of all the sub-fractioned populations, (2) the inability to expand the CD34$^-$ cells in our medium, (3) that only the PDGFRα^+SCA1$^+$CD34$^+$ fraction can be readily expanded further than P1, and (4) that the majority of the PDGFRα^+SCA1$^+$ cells are CD34$^+$ ($\sim$85% of all SCA1$^+$ cells are CD34$^+$), from here on, we only used PDGFRα^+SCA1$^+$ cells without sub-fractioning for CD34.

To decipher whether the organoid-supportive ability of the SCA1$^+$Msc cells relied on close proximity or soluble growth factors, we co-cultured DC and PDGFRα^+SCA1$^+$ cells within transwell-fitting plates to prevent cell contact between both populations (Figures 4B–4E). Under these conditions, we observed a remarkably similar 4-fold increase in organoid formation efficiency in DC/SCA1$^+$Msc co-cultures compared to DCs alone (Figure 4B), suggesting that the secreted growth factor repertoire of the SCA1$^+$Msc cells was directly responsible for supporting DC proliferation and organoid formation. To determine the nature of the organoid structures formed upon co-culture, we compared their molecular identity to that of control organoids grown in growth-factor-rich medium. For that, we performed RNA sequencing of DCs immediately after sorting (day 0) and following 15 days of culture alone in expansion medium (EM; medium supplemented with growth factors) or in a transwell co-culture with SCA1$^+$Msc cells in MM (medium devoid of growth factors) (Figures 4C–4E). Control organoids exposed to MM alone displayed minimal growth and could not be sequenced. The use of transwells enabled us to obtain the expression profile of each population independently before and after co-culture. Hierarchical clustering analysis revealed that organoids supported by SCA1$^+$Msc cells closely resembled organoids cultured in EM (Figure 4D). They expressed progenitor (*Tacstd2*, *Sox9*, and *Lgr5*) as well as DC markers (*Krt19* and *Epcam*) (Figure 4E), suggesting that SCA1$^+$Msc cells are capable of activating differentiated DCs to a proliferative state that enables organoid formation. Notably, the expression prolife, including lineage markers and secretome, of the SCA1$^+$Msc remained relatively unaltered upon 15 days in culture, either alone or when co-cultured with DCs in a transwell (Figure 4E), in agreement with our characterization upon passages (Figures S4D–S4G) and arguing against a phenotypic transformation *in vitro*. Remarkably, conditioned medium from serially passaged mesenchymal cells supported organoid formation at a similar mean efficiency (3.3%) compared with non-expanded SCA1$^+$Msc cells, suggesting that the cells remain functional even upon expansion (compare Figures 4F with Figures 4A and 4B). Immunofluorescence analysis indicated that the mesenchymal-supported organoids were formed by a single-layer epithelium of proliferative DCs (Krt19$^+$, Ki67$^+$), similar to organoids grown in complete medium (Figure 4G).

Collectively, these results highlighted that both freshly isolated and *in-vitro*-expanded SCA1$^+$Msc cells secrete pro-mitogenic factors that enable the activation of differentiated DCs into self-renewing liver organoids *in vitro*.

SCA1$^+$ mesenchymal cells dually control DC behavior by promoting or arresting DC proliferation in a cell-contact-dependent manner

The capacity of the PDGFRα^+SCA1$^+$ mesenchyme to induce DC proliferation and organoid formation *in vitro* resembled the context of a regenerating liver yet was seemingly at odds with the low proliferative index of the ductal epithelium in homeostasis, from where both cell populations derived. This led us to reexamine the fidelity of our culturing methods and to redesign a culture system that would recapitulate physiological liver architecture. At the PT, SCA1$^+$Msc cells are found in the immediate vicinity of DCs, physically wrapping the ductal epithelium (refer to Figures 1B–1D). Recapitulating this cell contact was crucial to characterize the cell interactions beyond paracrine signaling, yet such cell proximity became unavoidably disrupted following cell sorting, and our culturing methods using Matrigel droplets (Figure 4A), transwells (Figures 4B–4E), and conditioned media (Figure 4F) failed to reestablish it (Figure S4M).

Aiming to reconstitute the ductal-to-mesenchymal cell architecture of the PT *in vitro*, we tested a microfluidics-based approach for co-encapsulating ductal and mesenchymal cells into microgel droplets (70 μm in diameter), such that by restricting the spatial surroundings of the two cell types, we would increase the probability of their physical aggregation. As an experimental setup, we utilized ductal organoids and *in vitro* expanded SCA1$^+$Msc cells. The epithelial (GFP$^+$) and mesenchymal (tdTom$^+$) populations were resuspended in agarose and loaded separately onto custom-designed microfluidic flow-focusing devices (FFDs; Figures 5A, S5A, and S5B). As the encapsulation process follows a Poisson distribution, multiple permutations were observed, including separate encapsulation of both cell types and a large number of gels without cells (Figures 5A and S5C). Co-encapsulation (i.e., the presence of both cell types in one microgel) occurred in $\sim$6% of all events (Figure S5C).

Following encapsulation, the agarose microgels were embedded into Matrigel and cultured in MM for 4–5 days to allow organoid growth. No organoids were formed when seeding the microgels into agarose or in Matrigel, when encapsulation was performed in the absence of mesenchyme (Figures S5D–S5E). We detected the formation of multicellular complex organoids containing both ductal and mesenchymal cells that had established contact at an efficiency of $\sim$25% (Figure 5B). The layout of the Msc-ductal structures was reminiscent of the *in vivo* spatial arrangement, with the mesenchymal cells positioned on

CellPress
OPEN ACCESS

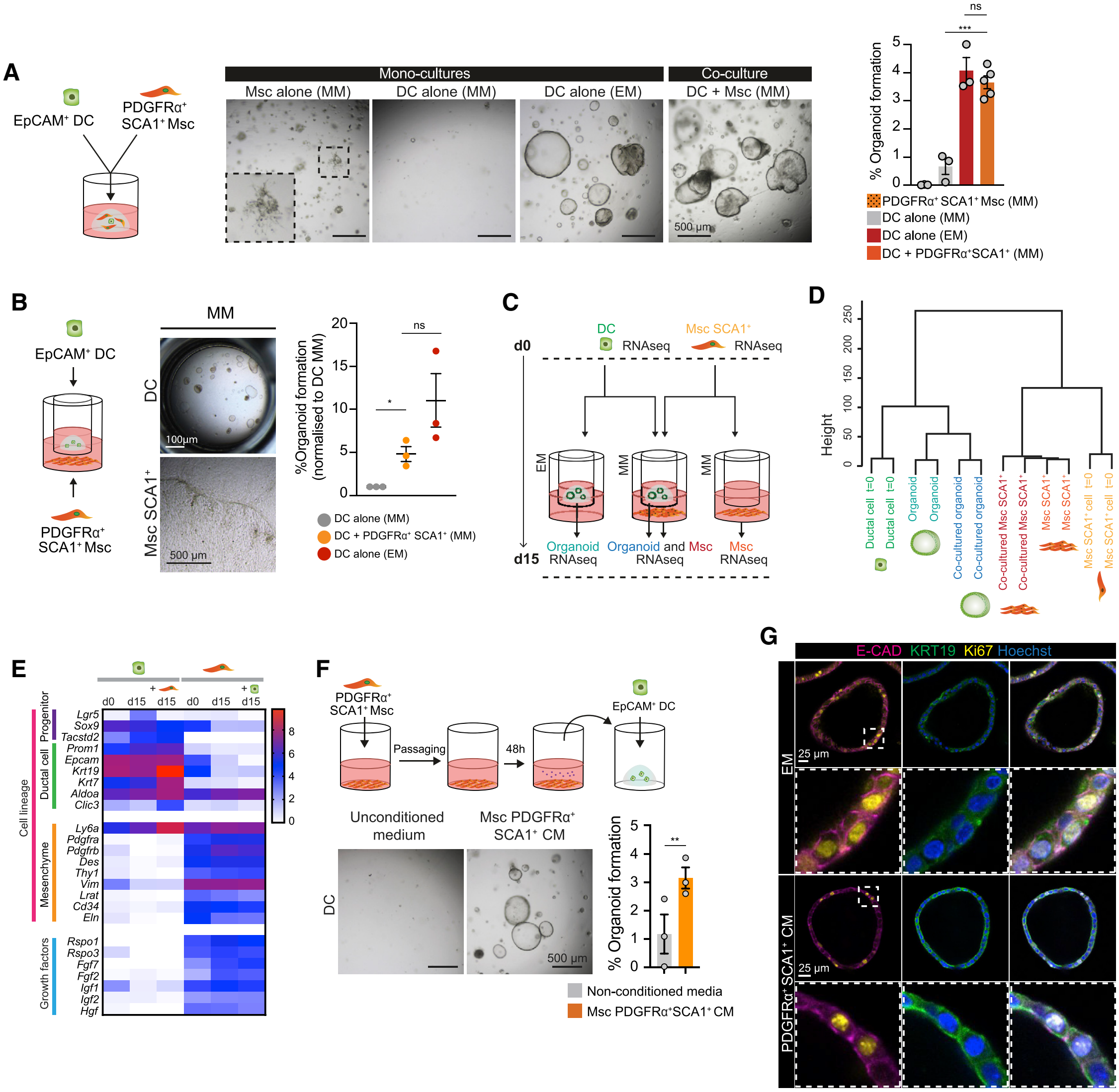

Figure 4. PDGFRα+SCA1+ mesenchymal cells support organoid formation via secreted growth factors

(A) Organoid-formation efficiency of EpCAM+ DCs and PDGFRα+SCA1+Msc cells cultured either alone (monoculture) or together (co-culture, 3,600 DCs and 18,000 Msc) in mesenchymal medium (MM) or DCs cultured alone in complete expansion medium (EM). Left: experimental design. Middle: representative brightfield images. Right: graph representing mean ± SEM of the percentage of organoid formation at day 10 obtained from at least n = 3 independent biological replicates. Student's t test; ***$p < 0.001$; ns, $p > 0.1$.

(B) Organoid-formation efficiency of freshly sorted EpCAM+ DCs seeded on a transwell insert alone in EM or MM or co-cultured for 10 days with freshly sorted PDGFRα+SCA1+ cells in MM. Left: schematic of a transwell co-culture. Middle: representative brightfield images of a transwell co-culture at day 10. Organoids, upper chamber. Msc, bottom chamber. Graph represents mean ± SEM of the percentage of organoid formation obtained from n = 3 independent biological replicates. Student's t test; *$p < 0.1$; ns, $p > 0.1$.

(C–E) RNA-seq analysis of DCs and SCA1+Msc sorted cells collected at day 0 (prior to culture), cultured alone or co-cultured in a transwell (day 15). (C) Experimental design. (D) Unsupervised clustering analysis of global mRNA expression in DCs and SCA1+Msc cells. (E) Heatmap representing the mean $\log_2$(TPM+1) value of the indicated genes from n = 2 independent biological replicates.

(F) PDGFRα+SCA1+ conditioned medium (CM) or unconditioned MM was added to freshly sorted EpCAM+ cells, and organoid formation was assessed at day 10. Top: experimental design. Bottom: representative brightfield images. Graph represents the percent organoid formation efficiency at day 10. Results are shown as mean ± SEM of n = 3 independent experiments, each with two biological replicates. Unpaired t test with Welsch correction; **$p < 0.01$.

(G) Immunofluorescence analysis of organoids derived from sorted EpCAM+ DCs cultured for 10 days in complete medium (EM) or Msc CM. Single composite z stack images of organoids stained for E-cadherin (magenta), KRT19 (green), proliferation (Ki67, yellow), and nuclei (blue). Representative images of n = 3 independent experiments.

See also Figure S4.

the basal surface of the biliary epithelium (compare Figures 5C and S5F with Figure 1B). We observed a preferential radial distribution (~51%) of the mesenchyme around the ductal structure, as it occurs *in vivo* (Figures S5F–S5H, category b), but we also encountered unilateral segregation of multiple SCA1+Msc cells (~28% of cases) (Figures S5F–S5H, category c). The organoids

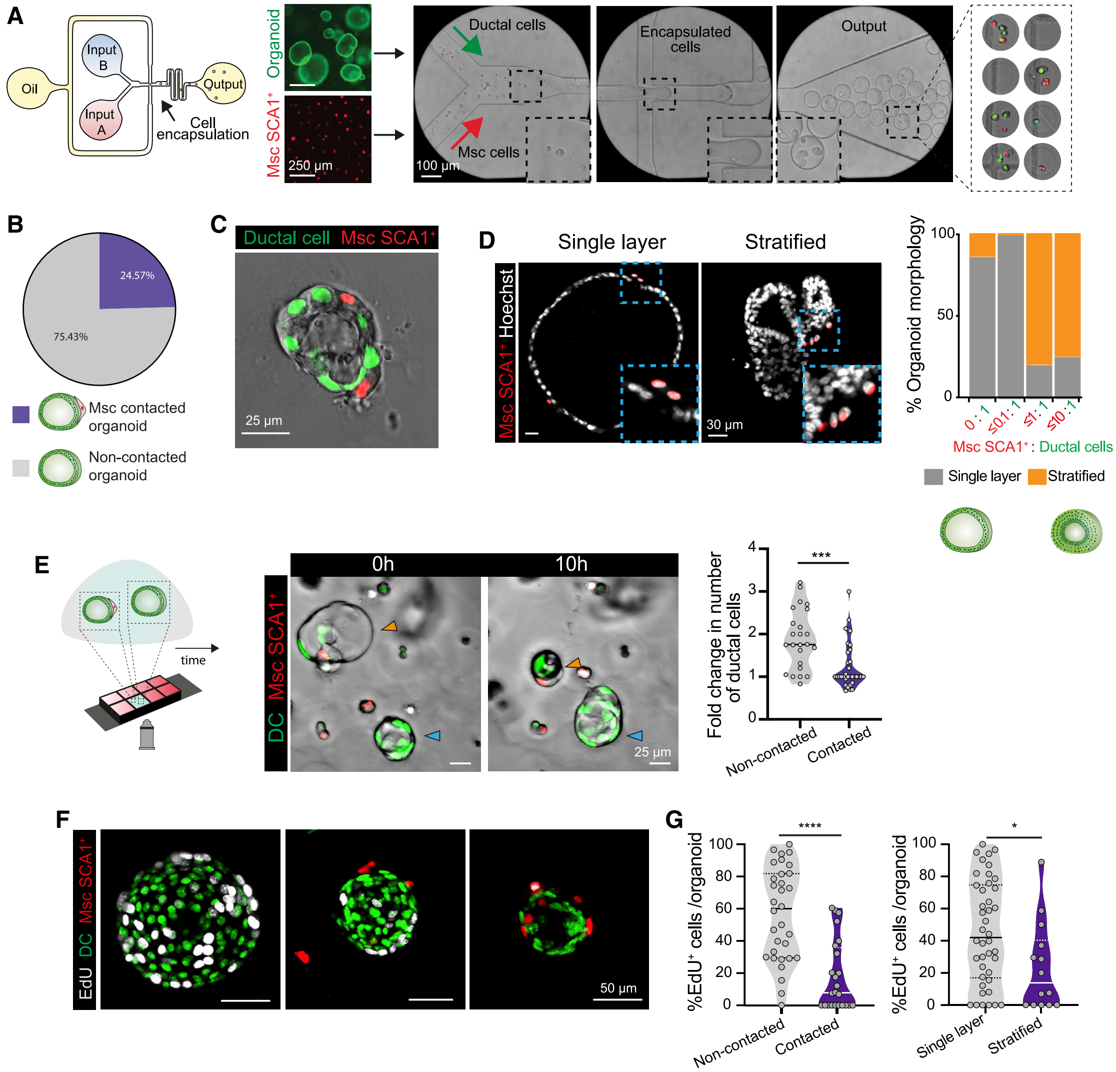

Figure 5. Mesenchymal-ductal organoids recapitulate the *in vivo* duct/Msc architecture of the portal tract

(A) Microfluidic setup for cell encapsulation of DCs and Msc, with a flow-focusing device (FFD) containing two separate inlets for cell loading (input A and input B, in aqueous phase), one inlet for the continuous phase (oil) and one outlet. Representative brightfield images of encapsulated microgels are shown.

(B) Frequency of formation of Msc-contacted organoids (containing DCs + SCA1^{+}Msc) at day 4 following encapsulation. n = 4 independent experiments.

(C) Representative composite single z stack image of a Msc-contacted organoid at day 4 post-encapsulation exhibiting a single-layer ductal (nuclear GFP^{+}) epithelium surrounded by mesenchymal (nuclear tdTom^{+}) cells on the periphery. See Figure S5F for additional examples.

(D) Representative composite single z stack immunofluorescence images of Msc-contacted organoids exhibiting cystic/single-layer epithelium (left) and stratified epithelium (right). Msc, red; nuclei, white. Percentage of organoid morphologies observed according to the ratio of SCA1^{+}Msc per DC. Data are presented as mean from n = 3 independent experiments.

(E) Time-lapse imaging (24 h) of Msc-contacted (nuclear GFP^{+} and nuclear tdTom^{+}) versus non-contacted (nuclear GFP^{+}) organoids grown within the same Matrigel droplet and culture medium. Left: experimental design. Middle: stills of a time-lapse at day 4 after co-encapsulation. Non-contacted organoid grows (blue arrowhead), while the Msc-contacted organoid collapses (orange arrowhead). Violin plot indicates the data-point distribution, median, and interquartile range (IQR) of the fold change on number of DCs following 24 h of imaging in non-contacted versus Msc-contacted structures obtained from n = 3 experiments. Dot, independent organoid. Mann-Whitney test; ***$p < 0.001$.

(F) Representative composite maximum projected z stacks images of organoids immunostained for EdU (white) assessed in day 5 co-cultures following incubation with 10 μM EdU for 16 h.

(G) Violin graphs represent the distribution, median, and IQR of the percentage of EdU^{+} DCs in non-contacted versus Msc-contacted organoids (left) and in single-layer/cystic versus stratified organoids (right) presented in (F) from n = 3 independent biological replicates. Mann-Whitney test; ****$p < 0.0001$; *$p < 0.1$. See also Figure S5 and Videos S2, S3, and S4.

in contact with mesenchymal cells exhibited additional diversity in terms of their epithelial architecture. We found some complex organoids retaining the single-layer epithelial architecture, with cells encircling a central lumen, typical of ductal organoids, while others presented a pseudo-stratified epithelium (Figure 5D). We noted that these two types of architectural arrangements

CellPress
OPEN ACCESS

correlated with the proportions of Msc and DCs within the complex organoids, such that structures with an Msc/DC ratio of $\leq$ 0.1 retained their single-layer epithelial architecture, but in ratios of >0.1, a stratified epithelium developed (Figure 5D).

To probe the effect of mesenchymal cell contact on DC expansion, we tracked individual organoids in culture and performed time-lapse imaging from day 4–5 of seeding, when the process of organoid formation had commenced. Surprisingly, we found a very interesting dichotomous behavior. The majority of non-Msc-contacted organoids (GFP$^+$ only) augmented in cell numbers and organoid area as time progressed (Figures 5E and S5I; Videos S2 and S3), as expected from being in the presence of mesenchyme-derived mitogens and reminiscent of our conditioned medium and transwell experiments (Figures 4B–4G). In stark contrast, mesenchyme-contacted organoids exhibited a significant paucity in growth (Figures 5E and S5I; Videos S2 and S4). This correlated with a reduced proliferative potential in the ductal compartment, assayed by EdU incorporation, which was exacerbated with higher doses of Msc contact (Figures 5F and 5G). Moreover, we noted a correlation between pseudo-stratified epithelial organization and decreased DC proliferation (Figure 5G). Mesenchymal cells forming part of multicellular complex organoids, on the other hand, rarely increased in numbers over the time assayed (Figure S5J).

These observations suggested that SCA1$^+$Msc cells regulate DC behavior in two ways: secreting pro-proliferative signals yet inducing growth arrest via cell contact.

SCA1$^+$Msc cells mediate DC proliferation arrest through Notch cell-cell contact inhibition

Having observed the paradoxical behavior of the SCA1$^+$Msc population *in vitro*, we hypothesized that it is the number of contacts and ratios between the two populations which ultimately control the DC state.

To test this, we opted to recapitulate the regeneration spectrum of PDGFRα^+SCA1$^+$/DC ratios *in vitro*. The microfluidics-based encapsulation method allowed this to a certain extent (Figure 5), but there was no exogenous control on the final output of the aggregated Msc/DCs, which hampered the systematic analysis of cell interactions at different ratios. We thus devised a contact-permissive co-culture method (Figures 6A and S6A), which generated multicellular organoids at an efficiency of 94.6% at a 1:1 ratio (Figure S6B), from which we inferred that the cell contact between both populations was directly proportional to the ratio seeded. This allowed the systematic study of cell interactions, both paracrine and cell bound, at the whole-population level instead of on an organoid-per-organoid basis as required with the microfluidics approach.

Using this co-culture method, we seeded increasing numbers of SCA1$^+$Msc (GFP$^+$) cells with a fixed number of sorted DCs (tdTom$^+$) both in MM medium (devoid of growth factors) and in complete medium (EM) (Figures 6B–6D). In MM, the ratio of 0.1:1 (PDGFRα^+SCA1$^+$ cells/DCs) resulted in a 3.8-fold increase in organoid formation relative to DCs alone (Figures 6B–6D), resembling the organoid formation efficiency obtained when DCs were co-cultured in transwell or using condition medium (see Figures 4B and 4F). The DC expansion at 0.1:1 was gradually reversed as epithelial-mesenchymal contacts augmented until nearly abolishing organoid growth at ratios 1:1 and higher (Figures 6B–6D). Remarkably, this effect could not be compensated by a mitogen-rich microenvironment (Figures 6B–6D, EM), highlighting the strong cytostatic effect of the contacting mesenchyme. Moreover, at ratios higher than 0.1:1 (Msc/DCs), we observed a negative correlation between mesenchymal cell dosage and the total number of dividing DCs, while the number of apoptotic DCs significantly increased (Figures 6E–6H, S6C, and S6D). Importantly, this phenotype was indeed reliant on physical contact between the two cell types, given that transwell co-cultures at a 5:1 ratio robustly promoted, instead of inhibited, organoid expansion (compare Figure S6E with Figures 6C and 6D). Interestingly, *in vitro* lineage tracing of *Lgr5*$^+$ progenitors –which are activated from differentiated DCs upon organoid culture – revealed a decreased percentage of proliferating *Lgr5*$^+$ cells in organoids contacted by the mesenchyme, even in the presence of complete medium containing RSPO1 and supplemented with WNT3a (EM + WNT) (Figures S6F and S6G).

Collectively, these results suggest a potential mechanism whereby it is the relative abundance of contacts between DCs and their mesenchymal niche cells what curtails the size of the ductal pool. Taking into account the strong correlation between the *in vivo* and *in vitro* results (see Figure 3 versus Figure 6) regarding the number of cellular ratios, cell contacts, and ductal proliferation, our co-culture results suggest a potential scenario whereby *in vivo*, during the damage-regenerative response, the Msc population could act as a direct upstream regulator of the ductal proliferative state.

To investigate the molecular basis for this contact inhibition, we examined our RNA-seq data in quest for proximity-based or juxtacrine signaling pathways wherein receptor(s) and ligand(s) could be paired between the two cell populations. We found that DC expressed *Notch1* and *Notch2*, *Tgfbr1* and *Tgfbr2* and the Hippo pathway downstream effectors *Yap1* and *Wwtr1*(TAZ), while SCA1$^+$Msc expressed Notch and Tgfb ligands (Figures 7A and S7A; Data S1). Then, we devised a small-scale screening assay using small-molecule inhibitors of these pathways where sorted DC were pre-treated with vehicle or the indicated inhibitor(s) prior to being co-cultured with SCA1$^+$Msc (Figure 7B). Pre-treated DCs were cultured alone or in the presence of SCA1$^+$Msc cells in a ratio expected to suppress organoid growth, and results were normalized to DC alone, to account for non-mesenchymal derived phenotypes (Figure 7C). Compared to controls, pre-treatment with the gamma secretase inhibitors DAPT and DBZ and the YAP inhibitor verteporfin (VP) yielded a significant increase in organoid formation, while TGFβ inhibitors had no significant effect (Figures 7C, 7D, and S7B). Interestingly, the combination between Notch and TGFβ inhibitors (DAPT or DBZ + A8301) also rescued organoid growth (Figure 7C). Notably, organoids arising from DAPT pre-treated DC (labeled by tdTom$^+$) were proliferative while still retaining physical interactions with the SCA1$^+$Msc cells (Figure 7E).

To identify the potential effectors/receptors that regulate the mesenchymal cell contact inhibition, we next performed a small-scale siRNA knockdown of some components of the pathways in DCs during co-culture with Msc. Notably, *Notch2*, but not *Notch1* knockdown significantly increased DC expansion (Figures 7F and S7C), while *Notch3* (Figures 7A and S7A), showed no effect, as expected for not being expressed.

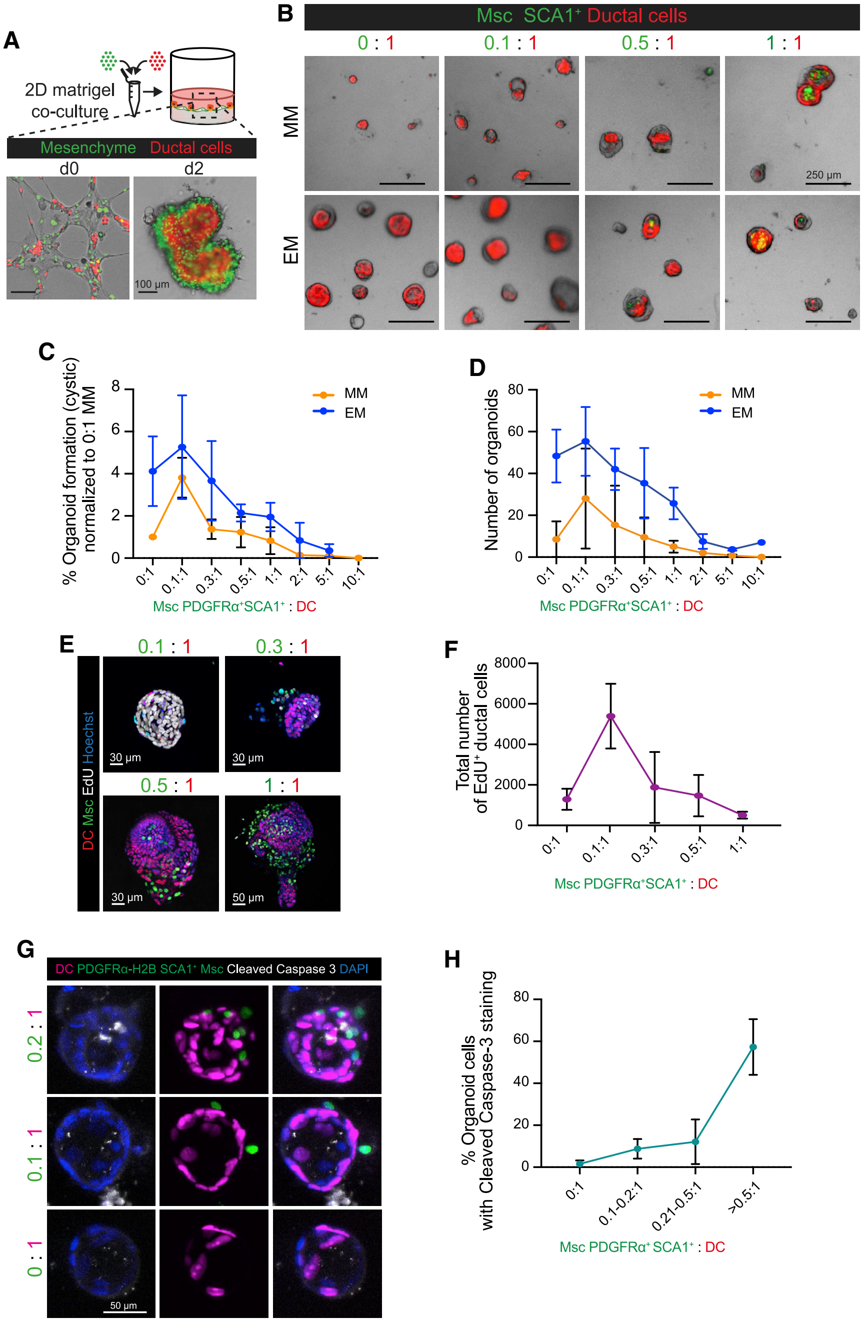

Figure 6. Dosage of cell contacts between PDGFRα[+]SCA1[+]Msc and DCs determines the net outcome of DC proliferation

(A) DCs (nuclear tdTom[+], red) were mixed at different ratios with PDGFRα[+]SCA1[+]Msc cells (nuclear GFP[+], green) and seeded on top of a 2D layer of Matrigel. Representative composite image of a 1:1 ratio co-culture at days 0 and 2.

(B and C) Freshly sorted DCs (red) were co-cultured with increasing ratios of SCA1[+]Msc cells in either growth-factor-devoid medium (MM) or growth-factor-rich medium (EM) for 8 days. (B) Representative composite images.

(C) Quantification of cystic/single-layer organoid formation efficiency at the indicated ratios in MM (orange) or EM (blue) at day 8, normalized to the DCs alone in MM (ratio 0:1). Graphs denote mean ± SD of n = 3 (EM) and n = 4 (MM) independent experiments. (D) Total numbers of organoids from (C).

(E and F) DC co-cultured with increasing numbers of SCA1[+]Msc cells were incubated with 10 μM EdU at day 6, and the number of proliferating cells was quantified 16 h later. (E) Representative composite images; EdU, white; nuclei, blue. (F) Graph representing the total number of EdU[+] DCs in the co-cultures at the indicated ratios. Mean ± SD of n = 2 independent experiment with >22 organoids per condition.

(G) Representative composite maximum intensity projection of Msc-contacted (nuclear green) and non-contacted organoids (nuclear magenta), stained for cleaved caspase-3 (white) at day 7. Nuclei, blue; the ratio of Msc to DC is specified for each structure.

(H) Percentage of DCs stained with cleaved caspase-3 in non-contacted or Msc-contacted structures; graph shows mean ± SD of n = 3 independent experiments with bins specified by the ratio of Msc to DC in each organoid structure.

See also Figure S6.

Given that Notch signaling is a well-known inducer of mature DC fate in hepatoblasts (Hofmann et al., 2010; Sparks et al., 2010; Zong et al., 2009) and adult hepatocytes (Jeliazkova et al., 2013), we decided to focus on this pathway as one of multiple potential mechanisms through which the mesenchyme modulates DC behavior in adulthood. To visualize Notch signaling in DCs upon Msc contact, we used *Hes1-GFP* mice (Klinck et al., 2011). In homeostatic livers, Hes1 expression was heterogeneous among DCs, even when all DCs were physically wrapped by SCA1[+]Msc (Figure S7D). DCs sorted from *Hes1-GFP* mice and cultured in complete medium generated organoids with limited GFP fluorescence (Figure S7E, 1), while isolated SCA1[+]Msc cells had undetectable *Hes1* expression (Figure S7E, 2). Co-culturing *Hes1-GFP* organoid cells with nuclear tdTom[+]SCA1[+]Msc cells at 1:1 ratio led to a higher percentage of *Hes1*-GFP DC relative to DC monocultures (Figure S7F). This was specific to co-cultures where cell contacts had been established, since Notch activation was not recapitulated upon addition of mesenchymal conditioned medium to DCs (Figure S7G). To better assess if contact was required for Notch signaling, we co-cultured *Hes1-GFP* ductal organoids with nuclear tdTom[+]SCA1[+]Msc cells at a 0.5:1 ratio so as to generate a mix of Msc-contacted and non-contacted organoid structures within the same well. Under these conditions, we found increased *Hes1-GFP* fluorescence in Msc-contacted structures (Figures 7G, 7H, and S7H). Notably, by using SCA1[+]Msc cells expressing a membrane-anchored tdTomato, we confirmed that mesenchymal-to-epithelial membrane contact activates Hes1-GFP expression in DCs (Figure 7I), albeit not in all cases (reminiscent

CellPress
OPEN ACCESS

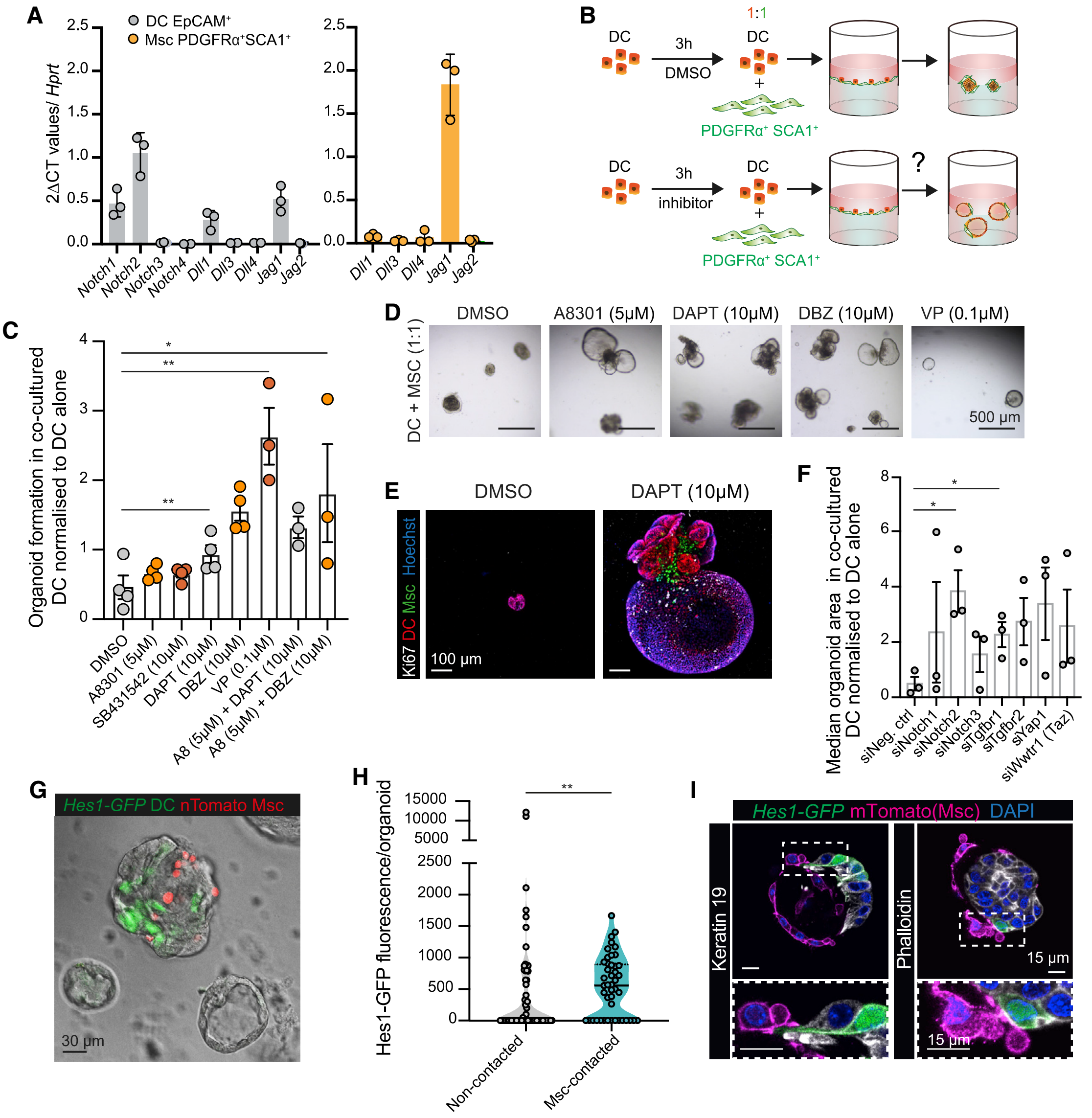

Figure 7. Cell contact from PDGFRα⁺SCA1⁺Msc cells inhibits DC proliferation via Notch signaling

(A) RT-qPCR gene expression analysis on selected genes of the Notch pathway in freshly sorted EpCAM⁺ DCs (gray bars) and PDGFRα⁺SCA1⁺Msc cells (orange bars). Graphs represent mean ± SD of n = 3 independent experiments.

(B–E) Freshly sorted EpCAM⁺ DCs (5,000 cells) were pretreated for 3 h with DMSO or the indicated inhibitors prior to being co-cultured at 1:1 ratio with 5,000 SCA1⁺Msc cells in MM. (B) Experimental design. (C) Graph represents cystic/single-layer organoid formation at day 10 in the DC/SCA1⁺Msc co-cultures normalized to that of the respective DC monocultures. Graphs display mean ± SEM from n ≥ 3 independent experiments. Student's t test (all treatments compared to the DMSO). **p < 0.01; *p < 0.1; ns, p > 0.1. (D) Representative bright-field images from day 10 co-cultures. (E) Maximum projected composite images of chimeric organoids (DC, red; Msc, green) immunostained for Ki67 (white) and nuclei (blue) at day 10 after DAPT treatment.

(F) Freshly sorted DCs (5,000 cells) were transfected with the indicated small interfering RNA (siRNA) and cultured alone or with 2,500 SCA1⁺Msc cells in MM at a 0.5:1 ratio (Msc/DCs). Organoid formation was assessed at day 10. Bar graph represents mean ± SEM of median organoid area normalized to the respective DC monocultures from n = 3 independent experiments. Student's t test (compared to control); *p < 0.1; ns, p > 0.1.

(G–I) Co-cultures between DCs sorted from *Hes1-GFP* mouse livers (green) and SCA1⁺Msc cells (ntdTom⁺, red) seeded at a 0.5:1 (Msc/DCs). The number of Hes1-GFP⁺ cells was assessed at day 8. (G) Representative bright-field and composite fluorescence image showing a contacted organoid (gray and red) with active Hes1-GFP (green) and non-contacted, Hes1-GFP⁻ organoids (gray).

(H) Graph represents the Hes1-GFP mean fluorescence intensity and area per z stack, normalized to total area, in non-contacted versus Msc-contacted organoids. Data are presented as violin plots showing data-point distribution, median, and IQR of n = 2 independent experiments (n = 46 Msc-contacted and 68 non-contacted organoids). Mann-Whitney test; **p < 0.01.

(I) Single z stack composite images of membrane tdTomato⁺ SCA1⁺Msc cells (magenta) establishing cell-cell contact with Hes1-GFP DCs. DC membranes were immunostained with Keratin-19 (white, left) or Phalloidin (white, right) and nuclei (blue).

See also Figure S7.

of native tissues, Figure S7D), likely due to this being a snapshot of an otherwise dynamic process. In addition, we found that activation of Notch signaling significantly diminished DC proliferation (Figure S7I).

Altogether, these results suggest a mechanism by which, at least *in vitro*, SCA1$^+$Msc cells induce DC proliferation arrest, in part by the juxtacrine activation of Notch signaling in DCs.

DISCUSSION

The regenerative capacity of the liver epithelium bespeaks not only cell-intrinsic plasticity but also an instructive microenvironment capable of guiding epithelial fate choices (Boulter et al., 2013). Previous work had reported on a hepatic population of SCA1$^+$ cells residing at the PT, which expanded in damaged livers (Clayton and Forbes, 2009) and contributed to fibrosis (Katsumata et al., 2017). Here, we identify PDGFRα^+SCA1$^+$ cells as a periportal mesenchymal subpopulation whose stoichiometry with respect to the ductal compartment, dynamic in regeneration, dictates its behavior as a pro-proliferative or a cytostatic niche. We demonstrate a very interesting paradox behind the relationship between mesenchymal and neighboring ductal populations. While PDGFRα^+SCA1$^+$ mesenchymal-secreted factors induce DC proliferation, cell contact is cytostatic. This growth-inhibitory effect overrides mitogenic signals, as it cannot be rescued by supplementation of a mitogen-rich medium. Our results might reconcile the apparent dichotomy behind a regenerative, pro-proliferative cellular environment and a pro-quiescent, post-mitotic homeostatic cellular environment. We suggest a new concept whereby it is the number of cell-cell contacts with the mesenchymal niche that determines the outcome (proliferation or cellular arrest) of DC behavior. While we formally demonstrate this paradox *in vitro*, using organoid co-cultures, future studies will aim at elucidating this mechanism *in vivo*.

It is well established that juxtacrine or contact-dependent signaling like that of Notch occurs either between adjacent cells or proximate neighbors aided by filopodia extensions (Cohen et al., 2010; De Joussineau et al., 2003); in contrast, secreted ligands such as FGF (Christen and Slack, 1999) and HGF (Patel et al., 2015) are diffusible and span a signaling range of multiple cell diameters (Perrimon et al., 2012). The integration of paracrine and juxtacrine signals antagonistic to each other can potentially explain part of the population dynamics between DC and SCA1$^+$Msc cells as follows: a low mesenchymal-to-DC ratio (0.1:1) maximizes DC proliferation via soluble factors while limiting mesenchymal contact to a few DCs; higher ratios, on the other hand, engage more DCs via juxtacrine signaling and eventually abolish DC proliferation. This is reminiscent of the concept of stem cell niche occupancy, whereby restrictions of niche factors, including abundance and signaling range, cause cells to compete with one another and regulate population asymmetry (Klein and Simons, 2011; Stine and Matunis, 2013). We showed an asynchrony in the DC and Msc expansion upon damage, with the DC expansion preceding that of SCA1$^+$Msc cells, thereby reducing the ratio and the number of cell contacts. An outstanding question is the stimulus that first signals for DC proliferation and spreading. Future in-depth studies will aim at addressing this question.

The ductal-mesenchymal organoids developed here were instrumental for deciphering the population dynamics and molecular crosstalk between both populations but also hinted at the possibility of modeling adult liver histoarchitecture in a dish. Complex liver buds have been previously generated using pluripotent stem cell [PSC]-derived epithelium and mesenchymal populations (Ouchi et al., 2019; Takebe et al., 2013), but not with primary adult liver populations. In our study, DC and SCA1$^+$Msc displayed cohesiveness by spontaneously aggregating with each other, a mechanism that could relate to mesenchymal-induced cell condensation (Takebe et al., 2015), yet promptly segregated into their respective compartments to recapitulate the spatial arrangement of homeostatic biliary ducts *in vivo*, where PDGFRα^+SCA1$^+$ cells wrap around but do not intermingle with the ductal epithelium. The principles governing this were beyond the scope of this work but are subject of great interest in understanding the self-organization of multicellular tissues (Takeichi, 2011).

In summary, our findings expand the concept of cellular niche in that it is the relative abundance of cell contacts, and not the absolute number of cells, that dictates the final outcome of epithelial proliferation during the different phases of the damage-regenerative response. Interestingly, in mouse prostate and muscle, mesenchymal SCA1$^+$ populations have also been found to modulate epithelial proliferation and myogenic differentiation, respectively (Joe et al., 2010; Wei et al., 2019). It would be of great interest to study whether our observations translate to human tissue once a direct homolog of *Sca-1/Ly6a* in humans is identified (Upadhyay, 2019). While our studies have focused on liver DC interactions in the PT area, we envision similar mechanisms at play in any other system where cell numbers dynamically change as a consequence of external cues, such as the lung or breast epithelium.

Limitations of the study

We focused on a subpopulation of mesenchymal PDGFRα^+ SCA1$^+$ cells. One limitation, though, is our inability to expand SCA1$^-$ fractions, which prevented us from testing whether the paradoxical behavior observed is a general feature of all or only a subset of liver mesenchymal cells. Another limitation is the low efficiency of co-encapsulation in our microfluidics system. Methods combining flow-based microfluidics could be leveraged to increase the yield of structures with the appropriate cellular ratios (Li et al., 2013). Also, while *in vitro* Notch signaling explains part of the cell contact inhibition mechanism, the plethora of behaviors observed upon direct mesenchymal-to-epithelial contact, including cell apoptosis and organoid collapse, cannot depend on a sole mechanism. It is tempting to speculate that it is the coalescence of several mechanisms, including biochemical signals and mechanical forces exerted by the Msc in the ductal epithelium, that explains the full phenotype. Accurately detangling one from the others will be a demanding yet not totally unsurmountable challenge to pursue in future studies.

STAR★METHODS

Detailed methods are provided in the online version of this paper and include the following:

- KEY RESOURCES TABLE
- RESOURCE AVAILABILITY
  - Lead contact

CellPress
OPEN ACCESS

- Materials availability
- Data and code availability
- EXPERIMENTAL MODEL AND SUBJECT DETAILS
  - Cell culture
  - Mouse models
- METHOD DETAILS
  - Liver ductal isolation
  - Flow cytometry
  - Matrigel co-culture
  - Conditioned medium and transwell co-cultures
  - 2D Matrigel co-cultures
  - Microfluidic chip production
  - Microfluidic cell encapsulation
  - Small molecule inhibitor and siRNA treatment
  - Mouse tissue sections staining
  - Organoid and mesenchyme staining
  - Time-lapse imaging and processing
  - qRT-PCR
  - RNA sequencing and analysis
  - Mesenchymal scRNaseq
- QUANTIFICATION AND STATISTICAL ANALYSIS
  - Organoid formation efficiency and size
  - Liver section analysis and processing
  - Fluorescence analysis
  - Statistics

SUPPLEMENTAL INFORMATION

Supplemental information can be found online at https://doi.org/10.1016/j.stem.2021.07.002.

ACKNOWLEDGMENTS

M.H. is a Lise Meitner Fellow from the Max-Planck-Gesellschaft. This work was funded by a Wellcome Trust Sir Henry Dale Fellowship awarded to M.H. (104151/Z/14/Z). L.C.-E. was funded by a Wellcome Trust Four-Year PhD Studentship from the Stem Cell Biology and Medicine Program. T.N.K. was supported by an AstraZeneca Graduate Studentship. N.C.H. is supported by a Wellcome Trust Senior Research Fellowship in Clinical Science (ref. 219542/Z/19/Z), Medical Research Council, and a Chan Zuckerberg Initiative Seed Network Grant. F.H. is an H2020 ERC Advanced Investigator (695669). P.S. received grants from the Novo Nordisk Foundation (NNF16076 and NNF10717). This work was partially funded by an H2020 LSMF4LIFE (ECH2020-668350 awarded to M.H.) and the Wellcome Trust (WT108438/C/15/Z awarded to F.H.) We acknowledge the Gurdon Institute core funding (Wellcome Trust 092096 and CRUK C6946/A14492). We also thank Mr. Robert Arnes-Benito for technical assistance, Prof. Anne Grapin-Botton and Prof. Magdalena Zernicka-Goetz for providing mouse strains, Mr. Kay Harnish and Dr. Charles Bradshaw (both from the Gurdon Institute), Dr. Jan Peychl and Dr. Sebastian Bundschuh (both from MPI-CBG), and the Gurdon Institute and MPI-CBG animal facilities for help with bioinformatics, imaging, and animal care; Dr. Andy Riddell (Cambridge Stem Cell Institute), Ms. Joana Cerveira (Department of Pathology, University of Cambridge), and Ms. Julia Jarrells and Ms. Ina Nuesslein (both MPI-CBG) for assistance with fluorescence-activated cell sorting (FACS); Dr. Maike Paramor (Cambridge Stem Cell Institute) for library preparation; and Mr. Hans Kleine-Brüggeney for the chip design.

AUTHOR CONTRIBUTIONS

M.H. and L.C.-E. designed the project. L.C.-E. and A.M.D. performed most of the experiments and, together with M.H., interpreted results. L.C.-E., A.M.D., and T.N.K. performed and F.H. supervised the microfluidics experiments. O.S., N.P., B.S., and G.B. performed experiments, and C.P. performed bioinformatics analyses. R.D., J.R.W.-K., and N.C.H. provided and analyzed the scRNA-seq data. R.B., F.J., and N.P.M. assisted with image analysis. P.S. provided mice. L.C.-E., A.M.D., and M.H. wrote the manuscript. All authors read and commented on the manuscript.

DECLARATION OF INTERESTS

M.H. is inventor in a patent on liver organoids and is on the advisory board of the journal *Cell Stem Cell*.

Received: November 28, 2019
Revised: May 19, 2021
Accepted: July 9, 2021
Published: August 2, 2021

REFERENCES

Aloia, L., McKie, M.A., Vernaz, G., Cordero-Espinoza, L., Aleksieva, N., van den Ameele, J., Antonica, F., Font-Cunill, B., Raven, A., Aiese Cigliano, R., et al. (2019). Epigenetic remodelling licences adult cholangiocytes for organoid formation and liver regeneration. Nat. Cell Biol. *21*, 1321–1333.

Apte, U., Thompson, M.D., Cui, S., Liu, B., Cieply, B., and Monga, S.P. (2008). Wnt/beta-catenin signaling mediates oval cell response in rodents. Hepatology *47*, 288–295.

Berger, D.R., Ware, B.R., Davidson, M.D., Allsup, S.R., and Khetani, S.R. (2015). Enhancing the functional maturity of induced pluripotent stem cell-derived human hepatocytes by controlled presentation of cell-cell interactions in vitro. Hepatology *61*, 1370–1381.

Bhatia, S.N., Balis, U.J., Yarmush, M.L., and Toner, M. (1998). Microfabrication of hepatocyte/fibroblast co-cultures: role of homotypic cell interactions. Biotechnol. Prog. *14*, 378–387.

Boulter, L., Govaere, O., Bird, T.G., Radulescu, S., Ramachandran, P., Pellicoro, A., Ridgway, R.A., Seo, S.S., Spee, B., Van Rooijen, N., et al. (2012). Macrophage-derived Wnt opposes Notch signaling to specify hepatic progenitor cell fate in chronic liver disease. Nat. Med. *18*, 572–579.

Boulter, L., Lu, W.Y., and Forbes, S.J. (2013). Differentiation of progenitors in the liver: a matter of local choice. J. Clin. Invest. *123*, 1867–1873.

Broutier, L., Andersson-Rolf, A., Hindley, C.J., Boj, S.F., Clevers, H., Koo, B.K., and Huch, M. (2016). Culture and establishment of self-renewing human and mouse adult liver and pancreas 3D organoids and their genetic manipulation. Nat. Protoc. *11*, 1724–1743.

Choi, T.Y., Ninov, N., Stainier, D.Y., and Shin, D. (2014). Extensive conversion of hepatic biliary epithelial cells to hepatocytes after near total loss of hepatocytes in zebrafish. Gastroenterology *146*, 776–788.

Christen, B., and Slack, J.M. (1999). Spatial response to fibroblast growth factor signalling in Xenopus embryos. Development *126*, 119–125.

Clayton, E., and Forbes, S.J. (2009). The isolation and in vitro expansion of hepatic Sca-1 progenitor cells. Biochem. Biophys. Res. Commun. *381*, 549–553.

Cohen, M., Georgiou, M., Stevenson, N.L., Miodownik, M., and Baum, B. (2010). Dynamic filopodia transmit intermittent Delta-Notch signaling to drive pattern refinement during lateral inhibition. Dev. Cell *19*, 78–89.

Coll, M., Perea, L., Boon, R., Leite, S.B., Vallverdú, J., Mannaerts, I., Smout, A., El Taghdouini, A., Blaya, D., Rodrigo-Torres, D., et al. (2018). Generation of Hepatic Stellate Cells from Human Pluripotent Stem Cells Enables In Vitro Modeling of Liver Fibrosis. Cell Stem Cell *23*, 101–113.e7.

Cordero-Espinoza, L., and Huch, M. (2018). The balancing act of the liver: tissue regeneration versus fibrosis. J. Clin. Invest. *128*, 85–96.

Davidson, M.D., Kukla, D.A., and Khetani, S.R. (2017). Microengineered cultures containing human hepatic stellate cells and hepatocytes for drug development. Integr. Biol. *9*, 662–677.

De Joussineau, C., Soulé, J., Martin, M., Anguille, C., Montcourrier, P., and Alexandre, D. (2003). Delta-promoted filopodia mediate long-range lateral inhibition in Drosophila. Nature *426*, 555–559.

Dobie, R., Wilson-Kanamori, J.R., Henderson, B.E.P., Smith, J.R., Matchett, K.P., Portman, J.R., Wallenborg, K., Picelli, S., Zagorska, A., Pendem, S.V.,

et al. (2019). Single-Cell Transcriptomics Uncovers Zonation of Function in the Mesenchyme during Liver Fibrosis. Cell Rep. *29*, 1832–1847.e1838.

Furuyama, K., Kawaguchi, Y., Akiyama, H., Horiguchi, M., Kodama, S., Kuhara, T., Hosokawa, S., Elbahrawy, A., Soeda, T., Koizumi, M., et al. (2011). Continuous cell supply from a Sox9-expressing progenitor zone in adult liver, exocrine pancreas and intestine. Nat. Genet. *43*, 34–41.

Gurtner, G.C., Werner, S., Barrandon, Y., and Longaker, M.T. (2008). Wound repair and regeneration. Nature *453*, 314–321.

Hamilton, T.G., Klinghoffer, R.A., Corrin, P.D., and Soriano, P. (2003). Evolutionary divergence of platelet-derived growth factor alpha receptor signaling mechanisms. Mol. Cell. Biol. *23*, 4013–4025.

Hofmann, J.J., Zovein, A.C., Koh, H., Radtke, F., Weinmaster, G., and Iruela-Arispe, M.L. (2010). Jagged1 in the portal vein mesenchyme regulates intrahepatic bile duct development: insights into Alagille syndrome. Development *137*, 4061–4072.

Hu, M., Kurobe, M., Jeong, Y.J., Fuerer, C., Ghole, S., Nusse, R., and Sylvester, K.G. (2007). Wnt/beta-catenin signaling in murine hepatic transit amplifying progenitor cells. Gastroenterology *133*, 1579–1591.

Huch, M., Dorrell, C., Boj, S.F., van Es, J.H., Li, V.S., van de Wetering, M., Sato, T., Hamer, K., Sasaki, N., Finegold, M.J., et al. (2013). In vitro expansion of single Lgr5+ liver stem cells induced by Wnt-driven regeneration. Nature *494*, 247–250.

Huch, M., Gehart, H., van Boxtel, R., Hamer, K., Blokzijl, F., Verstegen, M.M., Ellis, E., van Wenum, M., Fuchs, S.A., de Ligt, J., et al. (2015). Long-term culture of genome-stable bipotent stem cells from adult human liver. Cell *160*, 299–312.

Jeliazkova, P., Jörs, S., Lee, M., Zimber-Strobl, U., Ferrer, J., Schmid, R.M., Siveke, J.T., and Geisler, F. (2013). Canonical Notch2 signaling determines biliary cell fates of embryonic hepatoblasts and adult hepatocytes independent of Hes1. Hepatology *57*, 2469–2479.

Joe, A.W., Yi, L., Natarajan, A., Le Grand, F., So, L., Wang, J., Rudnicki, M.A., and Rossi, F.M. (2010). Muscle injury activates resident fibro/adipogenic progenitors that facilitate myogenesis. Nat. Cell Biol. *12*, 153–163.

Kan, N.G., Junghans, D., and Izpisua Belmonte, J.C. (2009). Compensatory growth mechanisms regulated by BMP and FGF signaling mediate liver regeneration in zebrafish after partial hepatectomy. FASEB J. *23*, 3516–3525.

Kapur, J.N., Sahoo, P.K., and Wong, A.K.C. (1985). A New Method for Gray-Level Picture Thresholding Using the Entropy of the Histogram. Comput Vision Graph *29*, 273–285.

Katsumata, L.W., Miyajima, A., and Itoh, T. (2017). Portal fibroblasts marked by the surface antigen Thy1 contribute to fibrosis in mouse models of cholestatic liver injury. Hepatol. Commun. *1*, 198–214.

Klein, A.M., and Simons, B.D. (2011). Universal patterns of stem cell fate in cycling adult tissues. Development *138*, 3103–3111.

Kleine-Brüggeney, H., van Vliet, L.D., Mulas, C., Gielen, F., Agley, C.C., Silva, J.C.R., Smith, A., Chalut, K., and Hollfelder, F. (2019). Long-Term Perfusion Culture of Monoclonal Embryonic Stem Cells in 3D Hydrogel Beads for Continuous Optical Analysis of Differentiation. Small *15*, e1804576.

Klinck, R., Füchtbauer, E.M., Ahnfelt-Rønne, J., Serup, P., Jensen, J.N., and Jørgensen, M.C. (2011). A BAC transgenic Hes1-EGFP reporter reveals novel expression domains in mouse embryos. Gene Expr. Patterns *11*, 415–426.

Krull, A.V.T., Prakash, M., Lalit, M., and Jug, F. (2020). Probabilistic Noise2Void: Unsupervised Content-Aware Denoising. Front. Comput. Sci. Published online February 19, 2020. https://doi.org/10.3389/fcomp.2020.00005.

Kumachev, A., Greener, J., Tumarkin, E., Eiser, E., Zandstra, P.W., and Kumacheva, E. (2011). High-throughput generation of hydrogel microbeads with varying elasticity for cell encapsulation. Biomaterials *32*, 1477–1483.

Lepreux, S., and Desmoulière, A. (2015). Human liver myofibroblasts during development and diseases with a focus on portal (myo)fibroblasts. Front. Physiol. *6*, 173.

Li, C.Y., Wood, D.K., Huang, J.H., and Bhatia, S.N. (2013). Flow-based pipeline for systematic modulation and analysis of 3D tumor microenvironments. Lab Chip *13*, 1969–1978.

Malato, Y., Naqvi, S., Schürmann, N., Ng, R., Wang, B., Zape, J., Kay, M.A., Grimm, D., and Willenbring, H. (2011). Fate tracing of mature hepatocytes in mouse liver homeostasis and regeneration. J. Clin. Invest. *121*, 4850–4860.

Mederacke, I., Hsu, C.C., Troeger, J.S., Huebener, P., Mu, X., Dapito, D.H., Pradere, J.P., and Schwabe, R.F. (2013). Fate tracing reveals hepatic stellate cells as dominant contributors to liver fibrosis independent of its aetiology. Nat. Commun. *4*, 2823.

Miyajima, A., Tanaka, M., and Itoh, T. (2014). Stem/progenitor cells in liver development, homeostasis, regeneration, and reprogramming. Cell Stem Cell *14*, 561–574.

Nguyen, T.V., Ukairo, O., Khetani, S.R., McVay, M., Kanchagar, C., Seghezzi, W., Ayanoglu, G., Irrechukwu, O., and Evers, R. (2015). Establishment of a hepatocyte-kupffer cell coculture model for assessment of proinflammatory cytokine effects on metabolizing enzymes and drug transporters. Drug Metab. Dispos. *43*, 774–785.

Otsu, N. (1979). Threshold Selection Method from Gray-Level Histograms. Ieee T Syst Man Cyb *9*, 62–66.

Ouchi, R., Togo, S., Kimura, M., Shinozawa, T., Koido, M., Koike, H., Thompson, W., Karns, R.A., Mayhew, C.N., McGrath, P.S., et al. (2019). Modeling Steatohepatitis in Humans with Pluripotent Stem Cell-Derived Organoids. Cell Metab. *30*, 374–384.e6.

Patel, D., Haque, A., Gao, Y., and Revzin, A. (2015). Using reconfigurable microfluidics to study the role of HGF in autocrine and paracrine signaling of hepatocytes. Integr. Biol. *7*, 815–824.

Perrimon, N., Pitsouli, C., and Shilo, B.Z. (2012). Signaling mechanisms controlling cell fate and embryonic patterning. Cold Spring Harb. Perspect. Biol. *4*, a005975.

Picelli, S., Faridani, O.R., Björklund, A.K., Winberg, G., Sagasser, S., and Sandberg, R. (2014). Full-length RNA-seq from single cells using Smart-seq2. Nat. Protoc. *9*, 171–181.

Pintilie, D.G., Shupe, T.D., Oh, S.H., Salganik, S.V., Darwiche, H., and Petersen, B.E. (2010). Hepatic stellate cells' involvement in progenitor-mediated liver regeneration. Lab. Invest. *90*, 1199–1208.

Prior, N., Inacio, P., and Huch, M. (2019). Liver organoids: from basic research to therapeutic applications. Gut *68*, 2228–2237.

Ramachandran, P., Dobie, R., Wilson-Kanamori, J.R., Dora, E.F., Henderson, B.E.P., Luu, N.T., Portman, J.R., Matchett, K.P., Brice, M., Marwick, J.A., et al. (2019). Resolving the fibrotic niche of human liver cirrhosis at single-cell level. Nature *575*, 512–518.

Raven, A., Lu, W.Y., Man, T.Y., Ferreira-Gonzalez, S., O'Duibhir, E., Dwyer, B.J., Thomson, J.P., Meehan, R.R., Bogorad, R., Koteliansky, V., et al. (2017). Cholangiocytes act as facultative liver stem cells during impaired hepatocyte regeneration. Nature *547*, 350–354.

Rockey, D.C., Boyles, J.K., Gabbiani, G., and Friedman, S.L. (1992). Rat hepatic lipocytes express smooth muscle actin upon activation in vivo and in culture. J. Submicrosc. Cytol. Pathol. *24*, 193–203.

Rossi, J.M., Dunn, N.R., Hogan, B.L., and Zaret, K.S. (2001). Distinct mesodermal signals, including BMPs from the septum transversum mesenchyme, are required in combination for hepatogenesis from the endoderm. Genes Dev. *15*, 1998–2009.

Schindelin, J., Arganda-Carreras, I., Frise, E., Kaynig, V., Longair, M., Pietzsch, T., Preibisch, S., Rueden, C., Saalfeld, S., Schmid, B., et al. (2012). Fiji: an open-source platform for biological-image analysis. Nat. Methods *9*, 676–682.

Schneider, C., Rasband, W., and Eliceiri, K. (2012). NIH Image to ImageJ: 25 years of image analysis. Nat. Methods *9*, 671–675.

Shen, K., Chang, W., Gao, X., Wang, H., Niu, W., Song, L., and Qin, X. (2011). Depletion of activated hepatic stellate cell correlates with severe liver damage and abnormal liver regeneration in acetaminophen-induced liver injury. Acta Biochim. Biophys. Sin. (Shanghai) *43*, 307–315.

Sparks, E.E., Huppert, K.A., Brown, M.A., Washington, M.K., and Huppert, S.S. (2010). Notch signaling regulates formation of the three-dimensional architecture of intrahepatic bile ducts in mice. Hepatology *51*, 1391–1400.

Stine, R.R., and Matunis, E.L. (2013). Stem cell competition: finding balance in the niche. Trends Cell Biol. *23*, 357–364.

Takase, H.M., Itoh, T., Ino, S., Wang, T., Koji, T., Akira, S., Takikawa, Y., and Miyajima, A. (2013). FGF7 is a functional niche signal required for stimulation of adult liver progenitor cells that support liver regeneration. Genes Dev. *27*, 169–181.

Takebe, T., Sekine, K., Enomura, M., Koike, H., Kimura, M., Ogaeri, T., Zhang, R.R., Ueno, Y., Zheng, Y.W., Koike, N., et al. (2013). Vascularized and functional human liver from an iPSC-derived organ bud transplant. Nature *499*, 481–484.

Takebe, T., Enomura, M., Yoshizawa, E., Kimura, M., Koike, H., Ueno, Y., Matsuzaki, T., Yamazaki, T., Toyohara, T., Osafune, K., et al. (2015). Vascularized and Complex Organ Buds from Diverse Tissues via Mesenchymal Cell-Driven Condensation. Cell Stem Cell *16*, 556–565.

Takeichi, M. (2011). Self-organization of animal tissues: cadherin-mediated processes. Dev. Cell *21*, 24–26.

Taymour, R., Kilian, D., Ahlfeld, T., Gelinsky, M., and Lode, A. (2021). 3D bioprinting of hepatocytes: core-shell structured co-cultures with fibroblasts for enhanced functionality. Sci. Rep. *11*, 5130.

Upadhyay, G. (2019). Emerging Role of Lymphocyte Antigen-6 Family of Genes in Cancer and Immune Cells. Front. Immunol. *10*, 819.

Ware, B.R., Durham, M.J., Monckton, C.P., and Khetani, S.R. (2017). A Cell Culture Platform to Maintain Long-term Phenotype of Primary Human Hepatocytes and Endothelial Cells. Cell. Mol. Gastroenterol. Hepatol. *5*, 187–207.

Wei, X., Zhang, L., Zhou, Z., Kwon, O.J., Zhang, Y., Nguyen, H., Dumpit, R., True, L., Nelson, P., Dong, B., et al. (2019). Spatially Restricted Stromal Wnt Signaling Restrains Prostate Epithelial Progenitor Growth through Direct and Indirect Mechanisms. Cell Stem Cell *24*, 753–768.e6.

Yang, J., Mowry, L.E., Nejak-Bowen, K.N., Okabe, H., Diegel, C.R., Lang, R.A., Williams, B.O., and Monga, S.P. (2014). β-catenin signaling in murine liver zonation and regeneration: a Wnt-Wnt situation! Hepatology *60*, 964–976.

Zack, G.W., Rogers, W.E., and Latt, S.A. (1977). Automatic measurement of sister chromatid exchange frequency. J. Histochem. Cytochem. *25*, 741–753.

Zaret, K.S. (2002). Regulatory phases of early liver development: paradigms of organogenesis. Nat. Rev. Genet. *3*, 499–512.

Zong, Y., Panikkar, A., Xu, J., Antoniou, A., Raynaud, P., Lemaigre, F., and Stanger, B.Z. (2009). Notch signaling controls liver development by regulating biliary differentiation. Development *136*, 1727–1739.

Zorn, A.M. (2008). Liver development. In StemBook (Harvard Stem Cell Institute).

STAR★METHODS

KEY RESOURCES TABLE

REAGENT or RESOURCE	SOURCE	IDENTIFIER
Antibodies		
Rat anti-Ly-6A/E (Sca-1) monoclonal (Clone D7), FITC	ThermoFisher Scientific	Cat# 11-5981-81; RRID: AB_465332
Rat anti-Ly-6A/E (Sca-1) monoclonal (Clone D7), PE	ThermoFisher Scientific	Cat# 12-5981-82; RRID: AB_466086
Rat anti-Ly-6A/E (Sca-1) monoclonal (Clone D7), Super Bright 436	ThermoFisher Scientific	Cat# 62-5981-82; RRID: AB_2637287
Rat anti-Ly-6A/E (Sca-1) monoclonal (Clone D7)	ThermoFisher Scientific	Cat# 14-5981-85; RRID: AB_467779
Rat anti- CD326 (EpCAM) monoclonal (Clone G8.8), APC	ThermoFisher Scientific	Cat# 17-5791-80; RRID: AB_2734965
Rat anti-CD31 monoclonal (Clone 390), PE-Cy7	Abcam	Cat# ab46733; RRID: AB_868905
Rat anti-CD31 monoclonal (Clone 390), PE-Cy7	BD Biosciences	Cat# 561410; RRID: AB_10612003
Rabbit anti-CD31 polyclonal	Abcam	Cat# ab28364; RRID: AB_726362
Rat anti-CD45 monoclonal (Clone 30-F11), PE-Cy7	BD Biosciences	Cat# 552848; RRID: AB_394489
Rat anti-CD45 monoclonal (Clone 30-F11), APC	ThermoFisher Scientific	Cat# 17-0451-83; RRID: AB_469393
Rat anti-CD11b monoclonal (Clone M1/70), PE-Cy7	BD Biosciences	Cat# 552850; RRID: AB_394491
Mouse anti-VEGF R3/Flt-4 Affinity Purified polyclonal	R&D Systems	Cat# AF743; RRID: AB_355563
Goat anti-Osteopontin Polyclonal	R&D Systems	Cat# AF808; RRID: AB_2194992
Rabbit anti-Cytokeratin, wide spectrum screening polyclonal	Agilent	Cat# Z0622; RRID: AB_2650434
Rat anti-Cytokeratin 19 monoclonal (Clone TROMA-III)	DSHB	Cat# TROMA-III; RRID: AB_2133570
Rat anti-F4/80 monoclonal (Clone CI:A3-1)	Abcam	Cat# ab6640; RRID: AB_1140040
Rabbit anti-Desmin polyclonal	Abcam	Cat# ab8592; RRID: AB_306653
Rabbit anti-Desmin polyclonal	Abcam	Cat# ab15200; RRID: AB_301744
Rabbit anti-Vimentin monoclonal (Clone EPR3776)	Abcam	Cat# ab92547; RRID: AB_10562134
Rabbit anti-alpha smooth muscle actin polyclonal	Abcam	Cat# ab5694; RRID: AB_2223021
Rabbit anti-CD34 monoclonal (Clone EP373Y)	Abcam	Cat# ab81289; RRID: AB_1640331
Rat anti-CD34 monoclonal (Clone RAM34), eFluor 660	ThermoFisher Scientific	Cat# 50-0341-82; RRID: AB_10596826
Goat anti-PDGFR- alpha polyclonal	R&D Systems	Cat# AF1062; RRID: AB_2236897
Goat anti-Reelin Affinity Purified polyclonal	R&D Systems	Cat# AF3820; RRID: AB_2253745
Rabbit anti-Ki67 (Ki-67) monoclonal (Clone SP6)	ThermoFisher Scientific	Cat# RM-9106-S1; RRID: AB_149792
Rabbit anti-Elastin polyclonal	CEDARLANE	Cat# CL55041AP; RRID: AB_10061195
Mouse anti-E-Cadherin monoclonal (Clone 34/E)	BD Biosciences	Cat# 610405; RRID: AB_397787
Mouse anti-Beta-Catenin monoclonal (Clone 14)	BD Biosciences	Cat# 610153; RRID: AB_397554
Rabbit anti-Phospho-Histone H3 (Ser10) polyclonal	Cell Signaling Technology	Cat# 9701; RRID: AB_331535
Rabbit anti-Cleaved Caspase-3 (Asp175) monoclonal (Clone 5A1E)	Cell Signaling Technology	Cat# 9664; RRID: AB_2070042
Chemicals, peptides, and recombinant proteins		
Collagenase from *Clostridium histolyticum*	Merck/Sigma	Cat# C9407
Dispase II	ThermoFisher Scientific	Cat# 17105-041
Fetal Bovine Serum	Merck/Sigma	Cat# F7524
Advanced DMEM/F-12	ThermoFisher Scientific	Cat# 12634010
DMEM, high glucose, GlutaMAX Supplement, pyruvate	ThermoFisher Scientific	Cat# 31966021
HEPES (1M)	ThermoFisher Scientific	Cat# 15630056
Penicillin/Streptomycin	ThermoFisher Scientific	Cat# 15140-122
GlutaMAX supplement	ThermoFisher Scientific	Cat# 35050-068

(*Continued on next page*)

CellPress
OPEN ACCESS

Continued

REAGENT or RESOURCE	SOURCE	IDENTIFIER
TrypLE Express Enzyme (1X), phenol red	ThermoFisher Scientific	Cat# 12605010
TrypLE Select Enzyme (10X), no phenol red	ThermoFisher Scientific	Cat# A1217701
B27-Supplement, serum free	ThermoFisher Scientific	Cat# 17504-044
N-2 Supplement	ThermoFisher Scientific	Cat# 17502-048
N-acetylcysteine (NAC)	Merck/Sigma	Cat# A9165
[Leu15]-Gastrin I Human	Merck/Sigma	Cat# G9145; CAS: 39024-57-2
Mouse EGF Recombinant Protein	ThermoFisher Scientific	Cat# PMG8041
Recombinant Human FGF-10	Peprotech	Cat# 100-26
Nicotinamide	Merck/Sigma	Cat# N0636
Rspondin 1 conditioned medium	Home made	Broutier et al., 2016 Nature Protocols
WNT3a conditioned medium	Home made	Broutier et al., 2016 Nature protocols
Recombinant Human HGF (Insect derived)	Peprotech	Cat# 100-39
Recombinant Human Noggin	Peprotech	Cat# 120-10C
Lipofectamine RNAiMAX	ThermoFisher Scientific	Cat# 13778030
ROCK inhibitor - Y-27632 dihydrochloride	Merck/Sigma	Cat# Y0503; CAS: 129830-38-2
Matrigel Growth Factor Reduced (GFR) Basement Membrane Matrix, Phenol Red-free	Corning	Cat# 356231
0.1% DDC (3,5-diethoxycarbonyl-1,4-dihydrocollidine) mouse diet	Custom Animal Diets, LLC	Cat# AD5001
Cell Recovery Solution	Corning	Cat# 354253
HFE-7500 3M (TM) Novec (TM) Engineered fluid	Fluorochem	Cat# 051243; CAS: 297730-93-9
Pico-Surf (5% (w/w) in Novec 7500)	Sphere Fluidics	Cat# C022
SeaPrep Agarose	Lonza	Cat# 50302
1H,1H,2H,2H-Perfluoro-1-octanol (PFO)	Merck/Sigma	Cat# 370533; CAS: 647-42-7
O.C.T. compound	VWR Chemicals	Cat# 361603E
Triton X-100	Merck/Sigma	Cat# T8787; CAS: 9002-93-1
Bovine serum albumin (BSA)	Merck/Sigma	Cat# A8806; CAS: 9048-46-8
Donkey serum	Merck/Sigma	Cat# D9663
Dimethyl sulfoxide (DMSO)	Merck/Sigma	Cat# D8418; CAS: 67-68-5
A8301 (TGF-βRI, ALK4 and ALK7 inhibitor)	TOCRIS	Cat# 2939/10; CAS: 909910-43-6
SB431542 (TGF-βRI, ALK4 and ALK7 inhibitor)	TOCRIS	Cat# 1614; CAS: 301836-41-9
DAPT (2S)-N-[(3,5-Difluorophenyl)acetyl]-L-alanyl-2-phenyl]glycine 1,1-dimethylethyl ester) (γ-secretase inhibitor)	Merck/Sigma	Cat# D5942; CAS: 208255-80-5
DBZ (Dibenzazepine) (γ-secretase inhibitor)	Merck/Sigma	Cat# D5942; CAS: 208255-80-5
Verteporfin	Merck/Sigma	Cat# SML0534; CAS: 129497-78-5
Vectashield	Vector Laboratories	Cat# H-1000-10
(Z)-4-Hydroxytamoxifen	Merck/Sigma	Cat# H7904-5MG; CAS: 68047-06-3
Hoechst 33342, Trihydrochloride, Trihydrate	ThermoFisher Scientific	Cat# H3570; CAS: 23491-52-3
SiR DNA	SpiroChrome	Cat# CY-SC007
DAPI	BD Biosciences	Cat# BD564907
Alexa Fluor Phalloidin 647	ThermoFisher Scientific	Cat# A22287
(Z)-4-Hydroxytamoxifen	Merck/Sigma	Cat# H7904-5MG; CAS: 68047-06-3
Critical commercial assays		
Click-iT EdU Alexa Fluor 594 Imaging Kit	ThermoFisher Scientific	Cat# C10339
Viability/Cytotoxicity Assay Kit for Animal Live & Dead Cells	Biotium	Cat# 30002
PicoPure RNA Isolation Kit	ThermoFisher Scientific	Cat# KIT0204
iTaq Universal SYBR Green Supermix	Bio-Rad	Cat# 172-5124
FastStart Essential DNA Green Master	Roche	Cat# 06402712001

(*Continued on next page*)

Continued

REAGENT or RESOURCE	SOURCE	IDENTIFIER
Deposited data		
RNA sequencing data of sorted cells and co-cultures	GEO	GEO: GSE140697
scRNA sequencing of mesenchymal populations	Dobie et al., 2019	N/A
Experimental models: Cell lines		
Mouse: liver mesenchyme SCA1$^+$	This paper	N/A
Mouse: liver mesenchyme SCA1$^+$ PDGFRα$^+$	This paper	N/A
Mouse: liver mesenchyme SCA1$^+$ PDGFRα$^+$ CD34$^+$	This paper	N/A
Mouse: liver mesenchyme SCA1$^-$ PDGFRα$^+$ CD34$^+$	This paper	N/A
Mouse: liver mesenchyme SCA1$^+$ PDGFRα$^+$ CD34$^-$	This paper	N/A
Mouse: liver mesenchyme SCA1$^-$ PDGFRα$^+$ CD34$^-$	This paper	N/A
Mouse: mouse embryonic fibroblasts (MEFs)	Gift from Ronald Naumann, MPI-CBG	N/A
Experimental models: organisms/strains		
Mouse: *Rosa26-mTmG* [Gt(ROSA)26Sortm4(ACTB-tdTomato,-EGFP)Luo/J]	The Jackson Laboratory	RRID: IMSR_JAX:007576
Mouse: *Rosa26-nTnG* [Gt(ROSA)26Sortm1(CAG-tdTomato*,-EGFP*)Ees]	The Jackson Laboratory	RRID: IMSR_JAX:023035
Mouse: Lgr5iresCreERT	Clevers Lab	Huch et al., 2013
Mouse: R26RtdTomato [Gt(ROSA)26Sortm9(CAG tdTomato)Hze/J]	The Jackson Laboratory	RRID: IMSR_JAX:007909
Mouse: Hes1-GFP [Tg(Hes1-EGFP)1Hri]	Serup Lab;	Klinck et al., 2011 Gene Expression Patterns
Mouse: *Pdgfra-H2B-GFP* [B6.129S4-Pdgfratm11 (EGFP)Sor/J]	Zernicka-Goetz Lab	Hamilton et al., 2003; RRID: IMSR_JAX:007669
Oligonucleotides		
See Table S3 *for qRT-PCR primers*	N/A	N/A
See Table S4 *for siRNA oligos*	Dharmacon	N/A
Software and algorithms		
ZEN	Zeiss	https://www.zeiss.com/microscopy/int/products/microscope-software/zen-lite.html
ImageJ/Fiji	Schneider et al., 2012	https://imagej.nih.gov/ij/
FlowJo	FlowJo	https://www.flowjo.com/
Huygens	Scientific Volume Imaging	https://svi.nl/
Noise to Void	Github	https://github.com/juglab/n2v
Liver Cell Distances	This paper	https://github.com/gurdon-institute/Liver-Cell-Distances
Chimeric Organoid Analyzer	This paper	https://github.com/gurdon-institute/Chimeric-Organoid-Analyser
Prism9	GraphPad	https://www.graphpad.com/scientific-software/prism/

RESOURCE AVAILABILITY

Lead contact

Further information and requests for resources and reagents should be directed to and will be fulfilled by the lead contact, Meritxell Huch (huch@mpi-cbg.de).

Materials availability

This study did not generate new unique reagents.

Data and code availability

The RNaseq datasets generated during this study are available at Gene Expression Omnibus (https://www.ncbi.nlm.nih.gov/geo/info/seq.html) under accession numbers GEO: GSE140697 .

CellPress
OPEN ACCESS

Software/packages used to analyze the dataset are either freely or commercially available. The custom scripts described in this manuscript are deposited on Github repository.

Noise To Void: https://github.com/juglab/n2v

Liver Cell Distances: https://github.com/gurdon-institute/Liver-Cell-Distances

Chimeric Organoid Analyzer: https://github.com/gurdon-institute/Chimeric-Organoid-Analyser

EXPERIMENTAL MODEL AND SUBJECT DETAILS

Cell culture

Organoids were cultured in AdDMEM/F12 (ThermoFisher, 12634010) medium containing HEPES (ThermoFisher, #15630-056), Penicillin/Streptomycin (ThermoFisher, #15140-122), Glutamax (ThermoFisher, #35050-068), 1% B27 (Invitrogen, #17504-044), 1% N2 (ThermoFisher, #17502-048) and 1.25mM N-acetylcysteine (Merck/Sigma, #A9165) –referred to as Basal medium–, which was further supplemented with 10nM gastrin (Merck/Sigma, #G9145), 50ng/ml mEGF (ThermoFisher, #PMG8043), 5% RSPO1 conditioned medium (homemade), 100ng/ml FGF10 (Peprotech, #100-26), 10mM nicotinamide (Merck/Sigma, #N0636) and 50ng/ml HGF (Peprotech, #100-39) –referred to as expansion medium (EM). Following isolation, EpCAM+ DC were embedded in Matrigel and cultured in EM supplemented with 30% WNT3a conditioned medium (WNT CM) (homemade), 25ng/ml Noggin (Peprotech, #120-10C) and 10 μM ROCK inhibitor (Ri) (Y-27632, Merck/Sigma, #Y0503) for 3 days and then were switched to standard EM. Organoids were passaged at a 1:3 ratio once a week or when fully grown through mechanical dissociation and re-embedded in fresh Matrigel and cultured in EM.

Mesenchymal cells were cultured in Basal medium supplemented with WNT CM (30%) referred to as mesenchymal medium (MM). Cells were passaged at 1:3 and 1:2 ratios, through enzymatic digestion using TrypLE Express (ThermoFisher, #12605010) for 5 min at 37°C. Ri was added to the MM when cells were seeded right after sorting or following passage. Mouse embryonic fibroblasts (MEFs) were cultured in DMEM (ThermoFisher, #31966021) supplemented with 10% FBS (Merck/Sigma, #F7524) and Penicillin/Streptomycin (ThermoFisher, #15140-122). Both mesenchymal cells and organoids were cultured in 37°C with 21% O_2 and 5% CO_2. When required cells were grown in 3% FBS in the absence of WNT CM.

Mouse models

Mouse experiments were performed under the Animal (Scientific Procedures) Act 1986 Amendment Regulations 2012 following ethical review by the University of Cambridge Animal Welfare and Ethical Review Body (AWERB). In addition, mouse experiments conducted in Germany were performed in accordance with the German animal welfare legislation and in strict pathogen-free conditions in the animal facility of the MPI-CBG. Protocols were approved by the Institutional Animal Welfare Officer (Tierschutzbeauftragter), and all necessary licenses were obtained from the regional Ethical Commission for Animal Experimentation of Dresden, Germany (Tierversuchskommission, Landesdirektion Dresden).

Mouse lines *Rosa26-nTnG* [Gt(ROSA)26Sortm1(CAG-tdTomato*,-EGFP*)Ees] and *Rosa26-mTmG* [Gt(ROSA)26Sortm4(ACTB-tdTomato,-EGFP)Luo/J] were obtained from the Jackson Laboratory (JAX). The *Rosa26-nGFP* line was obtained by germline recombination of the *Rosa26-nTnG* using a ubiquitous Cre. The Lgr5iresCreERT/RosatdTom was described in Huch et al. (2013) and kindly donated by Prof Hans Clevers (Hubrecht Institute). The Hes1-GFP was reported in Klinck et al. (2011) and kindly donated by Prof Anne Grapin-Botton (MPI-CBG). The *Pdgfra-H2B-GFP* was described in Hamilton et al. (2003) and obtained from Prof Magdalena Zernicka-Goetz. The *Pdgfra-H2B-GFP* mTmG mouse was obtained by crossing the *Pdgfra-H2B-GFP* and *Rosa26-mTmG* strains.

Mice were kept under standard husbandry in a pathogen-free environment with a 12 h day/night cycle. Sterile food and water were given *ad libitum*. Healthy adult mice (8-12 weeks of age) of both sexes were used for experiments. To induce liver damage, 8-12 weeks old mice were transferred to individual wheat-free cages and were fed with diet pellets supplemented with 0.1% DDC (3,5-diethoxycarbonyl-1,4- dihydrocollidine) (Custom Animal diets, LLC, #AD5001). Littermates from up to 3 litters of similar age and both sexes were randomly assigned to experimental groups. The diet was provided *ad libitum* for the duration of the experiment (up to 5 days or until weight drop reached a maximum of 20%), after which the mice were either sacrificed (DDC day 5) or switched back to normal chow to allow recovery (DDC day 5+6 days, DDC day 5+38 days). Untreated mice were used as controls (DDC Ctrl day 0). For each condition in each separate experiment, n = 3 mice were used, plus one additional mouse (n = 4) for the recovery groups to account any potential unexpected deaths. No mice were excluded from analysis.

METHOD DETAILS

Liver ductal isolation

To enrich for the biliary duct compartment, mouse livers were harvested and digested enzymatically as previously reported (Huch et al., 2013). In short, minced livers were incubated in a solution containing 0.0125% (mg/ml) collagenase (Merck/Sigma, #C9407), 0.0125% (mg/ml) dispase II (ThermoFisher, #17105-041) and 1% fetal bovine serum (FBS) (Merck/Sigma, #F7524) in DMEM/Glutamax (ThermoFisher, #31966-021) supplemented with HEPES (ThermoFisher, #15630-056) and Penicillin/Streptomycin (ThermoFisher, #15140-122) and 0.1 mg/ml of DNAase (Merck/Sigma, #DN25) in a shaker at 37°C and 150 rpm for 3h as detailed in Broutier et al. (2016). The biliary tree fragments and associated stroma were then dissociated into single cells with TrypLE diluted to 5x (GIBCO, #A12177-01).

Flow cytometry

For live cell sorting, single cells were incubated with fluorophore-conjugated antibodies for 30min and FACS-sorted using MoFlo Legacy, Astrios (Beckman Coulter), BD FACSAria (BD Biosciences) or SH800S (SONY) cell sorters. Cells were sequentially gated based on size and granularity (forward scatter, FSC, versus side scatter, SSC) and singlets (FSC-Area versus FSC-Height); after which ductal cells (DC) were selected based on EpCAM positivity and negative exclusion of the hematopoietic/endothelial markers CD31, CD45 and CD11b. The mesenchyme was enriched based on SCA1 positivity from the EpCAM$^-$CD31$^-$CD45$^-$CD11b$^-$ fraction, or in the case of *Pdgfra-H2B-GFP* mice, as double positive PDGFRα-GFP$^+$SCA1$^+$ cells gated from the EpCAM$^-$CD31$^-$CD45$^-$CD11b$^-$ fraction. For the isolation of PF and HSC the SCA1$^+$/SCA1$^-$ fractions were subsequently gated on CD34$^+$ (PF) and CD34$^-$ (HSC). Cells derived from *Rosa26-nTnG* or *Rosa26-mTmG* livers were further gated for tdTomato positivity. DC from *Hes1-GFP* livers were sorted as EpCAM$^+$CD31$^-$CD45$^-$CD11b$^-$ regardless of GFP positivity.

For analysis of *Hes1-GFP* expression following culture in conditioned medium (CM), 30 000 *Hes1-GFP* organoid cells were cultured in mesenchymal CM from PDGFRα$^+$SCA1$^+$ cells or non-conditioned media control (refreshed every 48h) for 8 days. For analysis of *Hes1-GFP* expression upon cell-cell contact with mesenchymal cells, 30 000 *Hes1-GFP* organoid cells were seeded alone or with 30 000 tdTomato$^+$SCA1$^+$ mesenchymal cells in EM + WNT CM (refreshed every 48h) on 2D Matrigel-layered 48-well plates for 8 days. *Lgr5CreERT2*, *R26-tdTomato* organoids were derived from *Lgr5CreERT2*, *R26-tdTomato* mice. 30 000 *Lgr5CreERT2*, *R26-tdTomato* organoid cells were cultured alone or co-cultured with PDGFRα-GFP$^+$SCA1$^+$ Msc cells on 2D Matrigel-layered 48-well plates overlaid with EM + WNT CM medium. On day 3, cultures were incubated with 10 μM of 4-hydroxytamoxifen (4-HT, Merck/Sigma, #H7904-5MG) and analyzed 24h later. Prior to all flow cytometric analysis, cultures were extracted from Matrigel with Cell Recovery solution (Corning, #354253), dissociated into single cells and analyzed with a Fortessa cell analyzer (BD Bioscience).

Matrigel co-culture

For matrigel bubble co-cultures, 3600 or 5000 freshly isolated and FACS-sorted ductal cells were embedded in a 25 μ.L Matrigel bubble with mesenchymal cells. The varying number of mesenchymal cells is indicated in the figure or figure legend (4000, 5000, 18 000, 25 000, 50 000, or 100 000) The co-culture was overlayed with 250 μL of MM. The organoid formation efficiency was assessed at day 7 after seeding.

Conditioned medium and transwell co-cultures

To generate mesenchymal conditioned medium (CM), sorted mesenchymal cells (PDGFRα$^+$SCA1$^+$) were first expanded *in vitro* (up to passage 2 or 3) as detailed above. When reaching 80%–90% confluency, cells were incubated with fresh MM medium. This was conditioned for 48h, centrifuged at 500 g for 10 min and filtered prior to being added to freshly sorted EpCAM$^+$ DC. For transwell co-cultures, freshly sorted or *in vitro* passaged mesenchymal cells were seeded on the bottom of 24 transwell-fitting plates (Corning, #3470) and cultured in MM medium for 5-7 days until reaching 80%–90% confluency. Freshly sorted 5000 EpCAM$^+$ DC were then seeded on top on cell-impermeable transwell inserts within a 25 μL drop of 100% Matrigel. Both the top and bottom compartments of the transwell were maintained in either Basal or MM for 10 days.

2D Matrigel co-cultures

For cell aggregation on 96–well plates pre-coated with a Matrigel-layer, single PDGFRα$^+$SCA1$^+$ cells and DC were mixed in the following mesenchyme-to- ductal cell ratios: 0:1, 0.1:1, 0.2:1, 0.5:1, 1:1, 1:2 and 5:1. In 96 well plate, for a 1:1 ratio co-culture 5000 DCs and 5000 Msc were used, and Msc adjusted accordingly in lower ratios (e.g., 5000 DCs and 2500 Msc in 0.5:1 ratio). After mixing, cells were centrifuged at 300 g for 5 min and seeded on top of a 2D-layer of solidified Matrigel (100%) covering the bottom of a 96-well plate. The medium of choice was dependent on experimental context, but consisted on either growth factor-reduced mesenchymal medium (MM) or complete organoid expansion medium (EM) supplemented with WNT CM to enhance mesenchymal cell survival. After 48h, organoids containing ductal and mesenchymal cells were detected.

Microfluidic chip production

Polydimethylsiloxane (PDMS) microfluidic chips were produced using soft lithography and replica molding as described elsewhere (Kleine-Brüggeney et al., 2019). Ductal and mesenchymal cells were co-encapsulated into microgels using a microfluidic flow-focusing device (FFD) that was a modified version of the microfluidic chip previously described in Kleine-Brüggeney et al. (2019) and Kumachev et al. (2011) and used to compartmentalize cells in droplets. Chips were designed to contain two separate inlets for the loading of two distinct cell populations (in aqueous phase): one inlet for the continuous phase (fluorinated oil HFE 7500 (Fluorochem, #051243)) containing 0.3% Pico Surf 1 surfactant (Sphere Fluidics, #C022) and one outlet. To maximize the chance of cell-cell encounters by proximity, the cross geometry of the chip, where droplet formation occurs, was limited to a width of 70 μm and a height of 75 μm. The AutoCAD Flow Focusing Device (FFD) Chip Design File used for production of PDMS FFD is available upon request.

Microfluidic cell encapsulation

EpCAM$^+$ ductal cells and SCA1$^+$ mesenchymal cells were isolated from *Rosa26-nGFP* and *Rosa26-nTnG* mice, respectively or vice versa, and were expanded *in vitro* as detailed above. The organoid and mesenchymal cell populations were dissociated into single cells, filtered through 40 μm cell strainers and resuspended as 0.75 × $10^{5\text{-}6}$ cells/50 μL of MM + Ri medium, respectively. The cell

suspensions were mixed with ultralow melting agarose solution (3% SeaPrep®, LONZA, #50302) in a volume ratio of 1:1 and were loaded onto the two aqueous phase inlets of the FFD. A flow rate of 3 μl/min was used for both aqueous phase channels and a flow rate of 30 μl/min for the continuous phase. The nascent emulsion droplet containing liquid agarose and cell suspension was collected in an ice cooled test tube resulting in agarose microgel formation. The gels were subsequently demulsified with 45 μl 1H,1H,2H,2H Perfluoro-1-octanol (PFO) (Merck, #370533) into 200 μL of MM+ Ri medium. μ-slide 8-well dishes (ibidi, #80826) were layered with 130 μL of ice-cold Matrigel/well and 10-15 μL of the microgel/cell suspension was seeded within each well. The cultures were maintained in MM medium.

Small molecule inhibitor and siRNA treatment

For the small-molecule inhibitor experiments, 10 000 freshly sorted EpCAM$^+$ DC were incubated in MM + Ri medium supplemented with one of the following inhibitors: A8301 (5 μM), SB431542 (10 μM), DAPT (10 μM), DBZ (10 μM) or Verteporfin (0.1 μM) or a combination of these, for 3h at 37°C. Cells treated with the same % of the vehicle DMSO were used as controls. The DC-treated cells were divided in half: 5000 cells were seeded alone as monoculture, 5000 were mixed with PDGFRα$^+$SCA1$^+$ in a 1:1 ratio. Cells were seeded in MM + Ri on top of a Matrigel-coated well in a 96wp as above. For the siRNA screen, 10 000 freshly sorted EpCAM$^+$ DC were transfected with 10pmol of a pool of 4 ON-Targetplus siRNA (Dharmacon) (see Methods S1D) for each candidate gene using Lipofectamine RNAimax (Life Technologies, # 13778030) according to manufacturer's instruction. Cells suspended in Basal + Ri medium were centrifuged for 45 minutes at 600 g at 32°C and then incubated 3h at 37°C. 5000 transfected DC were seeded alone, 5000 were co-cultured with PDGFRα$^+$SCA1$^+$ mesenchymal cells at 0.5:1 ratio in MM + Ri on 2D Matrigel-layered 96wp. Organoid formation was assayed at d10.

Mouse tissue sections staining

For tissue staining, livers were washed in PBS, diced with a razor blade and fixed for 2h or overnight in 10% formalin while rolling at 4°C. Tissues were then incubated with 30% sucrose PBS for 24-48h, embedded into cryomolds (Sakura, #4566) with OCT compound (VWR, #361603E) and snap-frozen. Tissue blocks were cryo-sectioned with a Leica CM-3050S cryostat or on Thermo Scientific CryoStar NX70 cryostat. For Ki67 staining, thick liver sections (100 μm) were blocked/permeabilized in PBS containing 1% Triton X-100 (Merck/Sigma, #T8787), 5% dimethyl sulfoxide (DMSO; Merck/Sigma, #D8418), 1% bovine serum albumin (BSA; Merck/Sigma #A8806) and 2% donkey serum (DS; Merck/Sigma, #D9663) for 16h at 4°C, and incubated with primary antibodies diluted in PBS + 0.5% Triton X-100, 1% DMSO, 2% DS for 72h at 4°C on an orbital shaker. Tissues were washed thoroughly over 24h with PBS + 0.5% Triton X-100 and 1% DMSO and then incubated with fluorophore-conjugated secondary antibodies in PBS + 0.5% Triton X-100, 1% DMSO and 2% DS for 48 at 4°C (see Methods S1A and S1B). Tissues were counterstained in PBS containing 1:1000 Hoechst 33342 (ThermoFisher, # H3570) for 1h and then washed in ascending glycerol concentrations (10%, 30%, 50%, 70%, 90%) for 1h. Sections were mounted in Vectashield (Vector Laboratories, #H-1000-10). All other liver immunostainings were performed on thin (8 or 12 μm) sections. For detection of surface antigens (e.g., SCA1), sections were blocked in PBS with 2% DS and 1% BSA for 2h at RT, incubated with primary antibodies in 1/100-diluted blocking buffer overnight at 4°C and with secondary antibodies for 2h at RT in 0.05% BSA PBS. Sections were counterstained with 1:1000 Hoechst for 10min and mounted in Vectashield. The stainings for PDGFRα, VEGFR3, β-catenin, and PCK were all enhanced with an additional Tris-EDTA pH9 antigen retrieval step (3min, 65°C) prior to blocking. Non-membrane stains were performed as above but with a blocking buffer supplemented with 0.5% Triton X-100. Images were acquired using a confocal microscope (Leica SP8 or Zeiss LSM 880) and processed using Volocity software (PerkinElmer), ZEN software (Zeiss), or ImageJ/Fiji.

For spectral unmixing of SCA1, OPN, and SiR DNA stainings in *Pdgfra-H2B-GFP mTmG* liver mouse section, 8 μm or 12 μm mouse tissue sections were imaged on Zeiss LSM 880 using a LD LCI Plan-Apochromat 40x glycerol immersion correction NA 1.2 objective (Zeiss). Laser lines at 405nm, 488nm, 561nm and 633nm were used to excite the fluorophores. Lambda mode scanning (detecting 410-687nm) was used to detect AF405, AF488, EGFP, tdTomato, AF633 and SiR-DNA. For all images, tile scans and z stacks were acquired with a step size of 1.1 μm and a pinhole of 20 airy unit. Images were taken at 1024x1024 voxel density with a line averaging of 8. Fluorophores and autofluorescence were unmixed into separate channels using the unmixing algorithm provided in the Zen software (Zeiss). Additionally, for each of the pictures a scan in the same Z stack was acquired using usual confocal set up as a control. Single stained slides were used to obtain the reference spectra of the different fluorophores. All composite pictures from stained tissue sections were obtained by merging the single channel images in FIJI/ImageJ.

Refer to Methods S1A and S1B for the complete list of primary antibody dilutions and secondary antibodies used.

Organoid and mesenchyme staining

For *in vitro* stainings, organoids and/or co-cultures were first extracted from Matrigel to facilitate immunostaining with ice-cold Cell Recovery solution (Corning, #354253) and then fixed with 4% paraformaldehyde (PFA) (Electron Microscopy Sciences, #15713-S) for 30min at RT; alternatively, cells were fixed *in situ* to preserve mesenchymal-to-epithelial interactions. Blocking and permeabilization was performed for 2h at RT in PBS containing 0.5% Triton X-100, 2% DMSO, 1% BSA and 2% DS. EdU incorporation assays were performed with the Click-iT® EdU Alexa Fluor 594 Imaging Kit (Life Technologies, #C10339) according to the manufacturer's protocol. Cells were incubated for 16h with 10 μM EdU in their respective culture medium, after which they were fixed in 4% PFA for 30 min, permeabilized with 0.5% Triton X-100 for 20 min and incubated with freshly prepared 1X Click-iT EdU cocktail for 30 min at RT. Nuclei were stained with Hoechst 33342 (Life Technologies, # 23491-52-3), DAPI (BD-Biosciences, # BD564907) or SiR-DNA (Spirochrome, # CY-SC007) for 15 min.

For Cleaved Caspase 3, pSer10-Histone3 and Krt19 staining, organoids were fixed with 4% PFA in Matrigel for 30min on ice, washed in 0.01% Triton X-100 PBS and permeabilized in 0.2% Triton X-100 PBS for 30min at RT. After 1hr blocking in 3% BSA 0.01% Triton X-100 PBS, the samples were incubated with primary antibodies overnight at 4°C in blocking solution. Following 3 washes with 0.01% Triton X-100 PBS, the samples were incubated 1hr at RT with secondary antibodies and Phalloidin/DAPI in blocking solution.

For live/dead staining in live cultures, Viability/Cytotoxicity Assay Kit for Animal Live & Dead Cells (Biotium, #30002) was used according to the manufacturer's protocol. Briefly, 2 μM Calcein and 4 μM EthDIII in PBS was added to cover the cells, and incubated for 30 min before washing, and imaging in normal media. Immunofluorescence images were acquired using a confocal microscope (Leica SP8 or Zeiss LSM 880) and processed using Volocity software (PerkinElmer), ZEN software (Zeiss), or ImageJ/Fiji. Live cell images were acquired in a Leica DMIL LED (brightfield only) using a Leica DF C450C camera or an EVOS FL (brightfield and fluorescence) microscope. Whole well pictures were acquired with a Leica M80 microscope using a Leica MC170 HD camera.

All composite pictures from stained cultures or organoids were obtained by merging the single channel images in FIJI/ImageJ.

Time-lapse imaging and processing

Time-lapse imaging of cells was carried out at 37°C and 5% CO_2 for 24h periods. A 20x air objective on a spinning- disk confocal microscope system (Intelligent Imaging Innovations, Inc. 3i) comprising an Observer Z1 inverted microscope (Zeiss), a CSU X1 spinning disk head (Yokogawa), and a QuantEM 512SC camera (Photometrics), was used to perform time-lapse imaging. Imaging was performed at 15 min intervals, with a z-step of 7 μm and a low laser power. A 10x air objective on a Zeiss 710 confocal microscope was also used perform time-lapse imaging at 15 min intervals, with a z-step of 9 μm, and 1024 × 1024 bidirectional scanning. Videos were generated with the Slidebook6 software and were analyzed with ImageJ/Fiji.

qRT-PCR

Total RNA was extracted from cells using the Arcturus PicoPure RNA Isolation Kit (Applied Biosystems, #12204-01) according to the manufacturer's protocol; including a 15 min digestion step with DNase to remove traces of genomic DNA. The RNA (50-250 ng) was reverse-transcribed with the Moloney Murine Leukemia Virus reverse transcriptase (M-MLVRT) (Promega, #M368B) and amplified using the iTaqTM Universal SYBR Green Supermix (Bio-Rad, #172-5124) on the CFX ConnectTM Real-Time PCR Detection System (Bio-Rad) or using FastStart Essential DNA Green Master (Roche, #06402712001) on the LightCycler 96 machine (Roche). The list of primers used for qRT-PCR is provided in Supplementary Methods S1C. Gene expression levels were normalized to the housekeeping gene *Hprt*, *18S* or *Gapdh* as specified in the graph axis labels. Refer to Methods S1C for the complete list of primer sequences used.

RNA sequencing and analysis

DC (EpCAM$^+$ CD45$^-$ CD11b$^-$ CD31$^-$) and mesenchymal/stromal sub-population (PDGFRα$^{+/-}$ SCA1$^{+/-}$ CD45$^-$ CD11b$^-$ CD31$^-$) hepatic fractions were sorted from three healthy mouse littermates for analysis of gene expression in homeostasis. For co-culture analyses, mesenchymal cells (SCA1$^+$ CD45$^-$ CD11b$^-$ CD31$^-$) from two littermates were first expanded on the bottom of 24 transwell-fitting plates (50000 cells/well) for 7 days in MM medium, after which freshly sorted DC (EpCAM$^+$ CD45$^-$ CD11b$^-$ CD31$^-$) from two other littermates were cultured on a cell-impermeable transwell insert (5000 cells/Matrigel bubble) alone in EM or in MM with the mesenchymal cells at the bottom for 15 days. Total RNA was extracted from all samples with the Picopure RNA Extraction Kit according to manufacturer's instructions (including DNase digestion).

RNA libraries were prepared using Smartseq2 (Picelli et al., 2014) and were sequenced on an Illumina HiSeq 4000 or Illumina HiSeq1500 instrument in single read mode at 50 base length. FastQC (version 0.11.4) was used for initial quality control of the reads. Reads were then mapped to the GRCm38/mm10 UCSC reference genome using STAR aligner (version 2.5.0a). Samtools was used to filter unmapped and low-quality reads (-F 1804 and -q 20). Raw counts were generated using featureCounts from the Rsubread package (version 1.24.2) including all exons for a gene from the mm10 GTF file (Mus_musculus.GRCm38.87.gtf). RPKMs were generated with raw counts and gene lengths reported by featureCounts. TPM (transcripts per million) and log_2(TPM+1) values were generated by normalizing the RPKM values. Dendograms were generated using hclust from the R stats package (version 3.5.1). Scaled RPKM values were used with Euclidean distance and the ward.d method for performing hierarchical clustering. For clustering of all samples, the top 2 000 most variable genes were used. Heatmaps were prepared based on TPM and logTPM values using the Prism9 software. All data has been deposited in GEO database. GEO accession number GSE140697 .

Mesenchymal scRNaseq

Data was obtained from Dobie et al. (2019) and analyzed for the expression of specific genes as detailed in their methods section (Dobie et al., 2019).

QUANTIFICATION AND STATISTICAL ANALYSIS

Organoid formation efficiency and size

Organoid formation efficiency was quantified by counting the total number of cystic/single layer (lumen-containing) organoid structures after 7-10 days in culture and normalizing it to the total number of EpCAM$^+$ cells seeded (typically 5000). Organoids were selected as regions of interest (ROI) with the blow/lasso tool and measured for area using Fiji (Schindelin et al., 2012).

Liver section analysis and processing

In order to quantify the relative positions of OPN^+ ductal cells from $PDGFR\alpha^+SCA1^+$ mesenchymal cells in liver tissue slices we developed Liver Cell Distances, a custom pipeline for Fiji implemented as a Jython script. Liver Cell Distances generates signal masks from maximum intensity z-projections using parameter sets appropriate for the size and morphology of the labeled structures of interest (Methods S1E). Single channel masks are combined to create SCA1/GFP and Hoescht/OPN double labeled area masks, allowing extraction of areas expressing SCA1 and GFP, and nuclei expressing OPN. To allow unsupervised use of automatic thresholding methods on images with varying signal levels including those with only background present, minimum intensity values can be set to discard mask areas containing raw mean intensity values too low to be signal of interest.

Distances from each OPN labeled nucleus with an area of at least 5 μm^2 to the nearest SCA1/GFP area are calculated by measuring the mean value of the SCA1/GFP signed Euclidean distance transform inside the nucleus. Liver Cell Distances script has been deposited in the publicly available GitHub repository: https://github.com/gurdon-institute/Liver-Cell-Distances. The analysis of liver sections with Desmin staining has been performed manually. The Desmin pictures have been denoised with Noise2Void (Krull et al., 2020), and were deconvolved with Huygens Professional version 19.04 (Scientific Volume Imaging, the Netherlands, https://svi.nl/), using a theoretical PSF and the CMLE algorithm with a SNR:20, 0.05 quality threshold and for a maximum of 40 iterations.

Fluorescence analysis

In order to quantify Hes1-GFP and tdTomato fluorescence within organoid structures, we developed Chimeric Organoid Analyzer, a script for Fiji that automatically applies custom segmentation pipelines for each of the image channels. Chimeric Organoid Analyzer measures organoid area in a single slice of a z stack chosen for optimal focus and measures the area of GFP and Tomato signal inside the organoid. Organoid area is mapped by calculating smoothed local variance and applying the Triangle automatic thresholding method (Zack et al., 1977). GFP signal is segmented using the Otsu threshold (Otsu, 1979) on smoothed signal, and Tomato-containing cell clusters are segmented using Kapur's maximum entropy threshold (Kapur et al., 1985) on difference of Gaussians processed images. These methods were chosen to detect the features of interest in each channel, namely textured regions, large, homogeneous signal areas and discrete clusters of cells in the brightfield, GFP and Tomato channels respectively. Chimeric Organoid Analyzer has been deposited in the publicly available GitHub repository: https://github.com/gurdon-institute/Chimeric-Organoid-Analyser.

Statistics

Data were analyzed as detailed in Figure legends and as appropriate for each experiment by using Mann–Whitney test, Welch's t test, unpaired t test with Welch's correction or a Student's t test. $p < 0.05$ was considered statistically significant. Calculations were performed using the Prism 9 software package. All P values are given in the corresponding figure legends. Dispersion and precision measures (e.g., mean, median, SD, SEM) are specified in the figure legends. All the independent and biological replicates are specified in figure legends and Supplementary Table S1. Additionally, we provide Table S1 with all the manual quantification data.

Cell Stem Cell, Volume *28*

Supplemental Information

Dynamic cell contacts between periportal mesenchyme and ductal epithelium act as a rheostat for liver cell proliferation

Lucía Cordero-Espinoza, Anna M. Dowbaj, Timo N. Kohler, Bernhard Strauss, Olga Sarlidou, German Belenguer, Clare Pacini, Nuno P. Martins, Ross Dobie, John R. Wilson-Kanamori, Richard Butler, Nicole Prior, Palle Serup, Florian Jug, Neil C. Henderson, Florian Hollfelder, and Meritxell Huch

Cordero-Espinoza et al._Figure S1

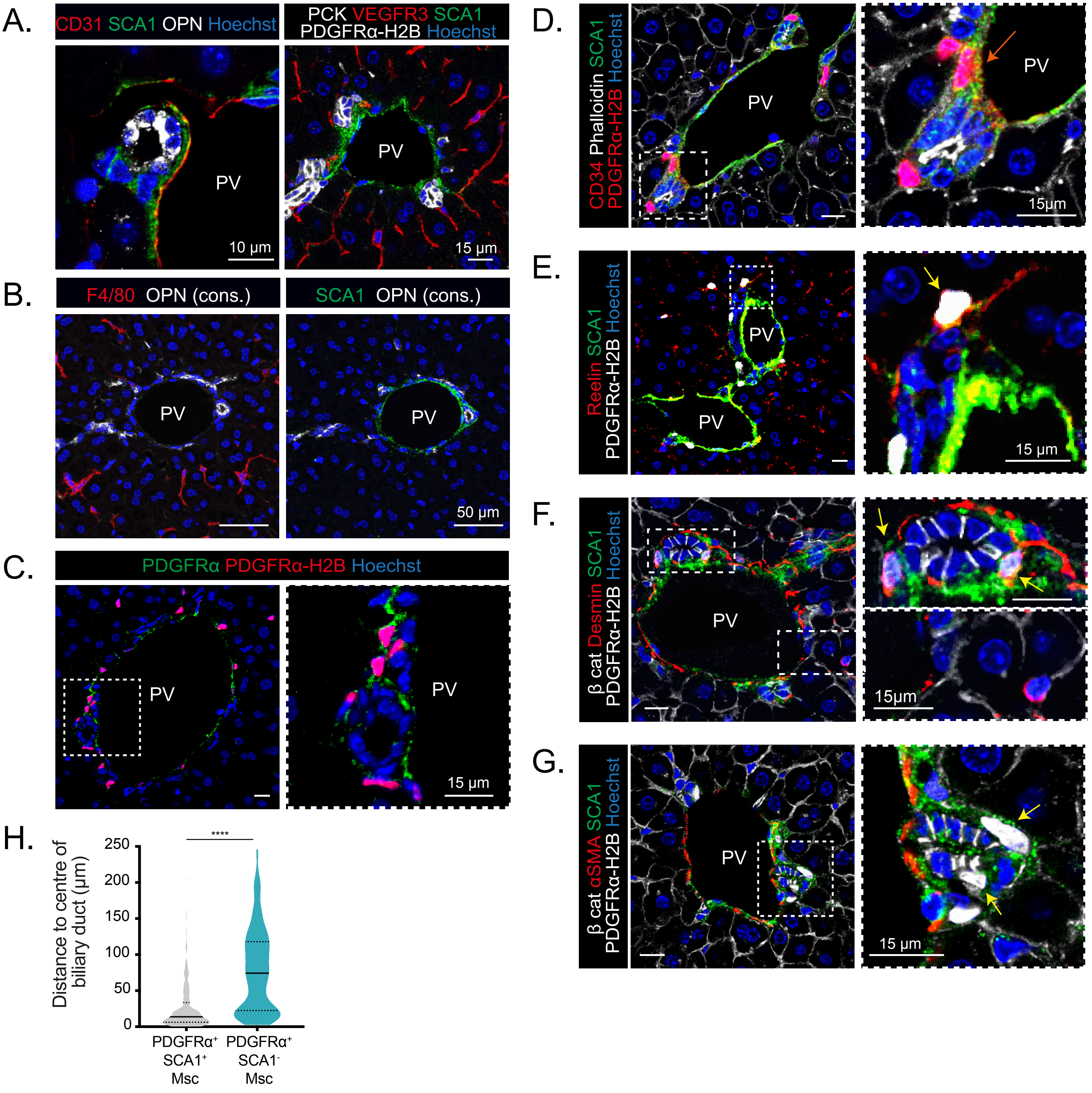

Figure S1. Periportal SCA1⁺ cells express mesenchymal markers and are close to the bile duct epithelium. Related to Figure 1.

A-G) Immunofluorescence analysis of WT (A left, B) and Pdgfra-H2B-GFP (A right, C-G) mouse livers indicates that the PDGFRα$^+$SCA1$^+$ cell population at the portal tract co-stains with CD34, desmin and Reelin, but not with α-SMA, CD31, VEGFR3, and F4/80. Images are presented as single z-stacks and nuclei are all counterstained with Hoechst (blue). PV, portal vein. Composite pictures were obtained by merging the single channel images in FIJI/ImageJ.

A) SCA1 (green) immunostaining with CD31 (red, left panel) and VEGFR3 (red, right panel) endothelial markers, and the ductal cell markers Osteopontin (OPN, white, left panel) or pancytokeratin (PCK, white membrane, right panel).

B) Consecutive (cons) 5µm-liver sections stained with the ductal marker Osteopontin (OPN, white) and the macrophage marker F4/80 (red) (left panel) or SCA1 (green) (right panel).

C) PDGFRα immunostaining (green) in Pdgfra-H2B-GFP (nuclear red) mouse livers indicate that the reporter faithfully recapitulates endogenous PDGFRα expression.

D) Representative single z-stack images of Pdgfra-H2B-GFP (nuclear red) mouse livers co-stained with SCA1 (green), the portal fibroblast marker CD34 (red) and the actin marker Phalloidin (white, membrane). Orange arrow, PDGFRα$^+$SCA1$^+$ Msc.

E) SCA1 (green) immunostaining with the hepatic stellate cell marker Reelin (red) in Pdgfra-H2B-GFP (white) mouse livers. Yellow arrow, PDGFRα$^+$SCA1$^+$ Msc.

F) Representative single z-stack images of Pdgfra-H2B-GFP (nuclear white) mouse livers co-stained with SCA1 (green), mesenchymal marker Desmin (red) and epithelial marker β-catenin (white, membrane). Yellow arrows, PDGFRα$^+$SCA1$^+$ Msc.

G) SCA1 (green) immunostaining with the pericyte marker α-SMA (red) and the epithelial marker β-catenin (white, membrane) in Pdgfra-H2B-GFP (white, nuclear) mouse livers. Yellow arrows, PDGFRα$^+$SCA1$^+$ Msc.

H) Violin plot graph representing the distribution, median and IQR of distances between PDGFRα$^+$SCA1$^+$ Msc and PDGFRα$^+$SCA1$^-$ Msc to the center of the nearest biliary duct in homeostatic liver sections (n=3). P-value was calculated using Mann Whitney test, $p<0.0001$ (****).

Cordero-Espinoza et al._Figure S2

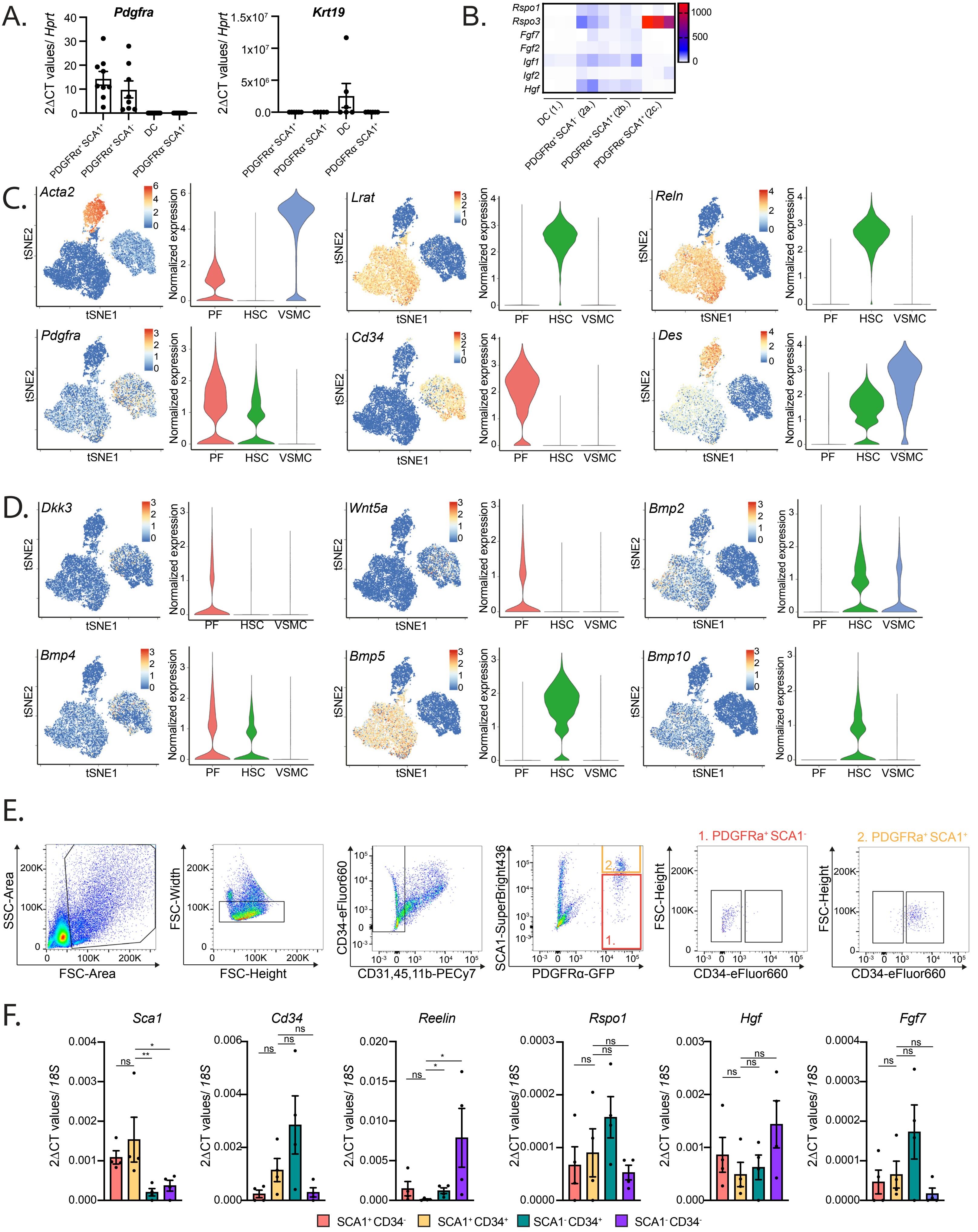

Figure S2. scRNAseq analysis on liver mesenchymal populations. Related to Figure 2.

A) mRNA expression levels of selected genes measured via RT-qPCR in freshly sorted DC and specified niche cells; Graph represents mean ± SEM on n=9 (Pdgfrα) or n=6 (Krt19) (DC, PDGFRα$^+$SCA1$^+$, PDGFRα$^-$SCA1$^+$ Msc), n=8 (Pdgfrα) or n=5 (Krt19) (PDGFRα$^+$SCA1$^-$ Msc) biological replicates (mice) from 3 independent experiments.

(B) Heatmap representing TPM values of the indicated secretome genes from n=3 biological replicates from the RNAseq analysis of ductal cells (DC), mesenchymal PDGFR$^+$SCA1$^+$ and PDGFRα$^+$SCA1$^-$ cells and stromal PDGFRα$^-$SCA1$^+$ cells. The numbers (2a, 2b, 2c) correspond to sorted populations in Figure 2B.

C-D) scRNAseq analysis of sorted mouse hepatic mesenchymal cell populations published in Dobie *et al*., 2019. tSNE plots show the expression of the indicated genes in each mesenchymal cluster. Violin plots indicate the data point distribution of gene expression for the indicated genes. PF: portal fibroblasts, HSC: hepatic stellate cells. VSMC vascular smooth muscle cell.

(E) FACS sorting strategy to separate the portal fibroblasts from hepatic stellate cells (HSC) in the PDGFRα$^+$SCA1$^+$ and PDGFRα$^+$SCA1$^-$ fractions, based on the portal fibroblast specific marker CD34.

(F) qRT-PCR gene expression analysis of Sca1, Cd34 and Reelin markers, and selected secretome genes Rspo1, Hgf and Fgf7, in the specified mesenchymal subpopulations (PDGFRα$^+$SCA1$^+$CD34$^-$, red; PDGFRα$^+$SCA1$^+$CD34$^+$, yellow; PDGFRα$^+$SCA1$^-$CD34$^+$, green; PDGFRα$^+$ SCA1$^-$CD34$^-$, purple). Graph represents the mean ± SEM of n=4 biological replicates in 3 independent experiments. p-values were obtained using Mann-Whitney test.

Cordero-Espinoza et al._Figure S3

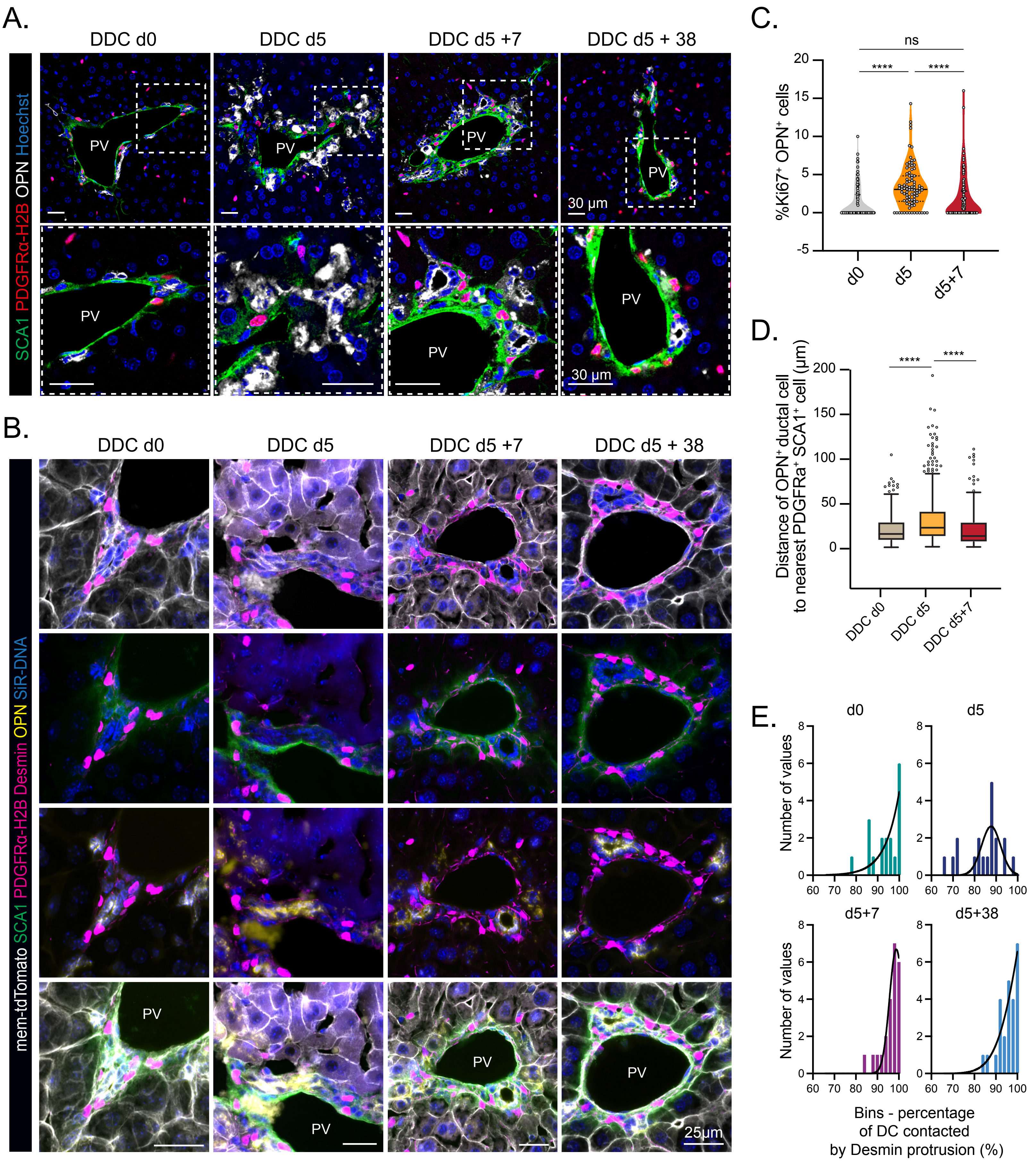

Figure S3. SCA1$^+$ mesenchyme in vivo during regeneration. Related to Figure 3.
A) Representative single z-stack images of livers from Pdgfra-H2B-GFP (nuclear red) mice damaged as above and stained for SCA1 (green) and OPN (white). Nuclei were counterstained with Hoechst (blue). PV, portal vein. Composite pictures were obtained by merging the single channel images in FIJI/ImageJ.
(B) Representative maximum intensity projection images of livers from Pdgfra-H2B-GFP/mTmG (nuclear magenta and membrane white) mice before (d0), during damage (d5) and and at different time points after damage (d5+7, d5+38) as detailed in methods. Sections were stained for desmin (magenta), SCA1 (green) and OPN (yellow). Nuclei were stained with SiR-DNA (blue). Composite pictures were obtained by merging the single channel images in FIJI/ImageJ.
C) Violin plot graph representing the data point distribution, median and IQR of the percentage of Ki67$^+$ OPN$^+$ ductal cells in undamaged (d0), d5 (damaged) and d5+7 (recovery) livers from n=3 independent experiments. p-values were obtained via Mann-Whitney t-tests. d0 vs d5 and d5 vs d5+7, p<0.0001 (****); d0 vs d5+7, p=0.1168 (ns).
D) Box and whiskers Tukey plot (median, whiskers are 1.5 interquartile range) represents the distance between OPN$^+$ DC and PDGFRα$^+$SCA1$^+$ Msc cells (see methods) in DDC-damaged livers at d0, d5 and d5+7 (n=2). Dots are outliers. P-values were obtained via Mann-Whitney tests. ****, p<0.0001.
(E) Frequency distribution and Gaussian curve of the percentages for contacts, presented in Figure 3B.

Cordero-Espinoza et al._Figure S4

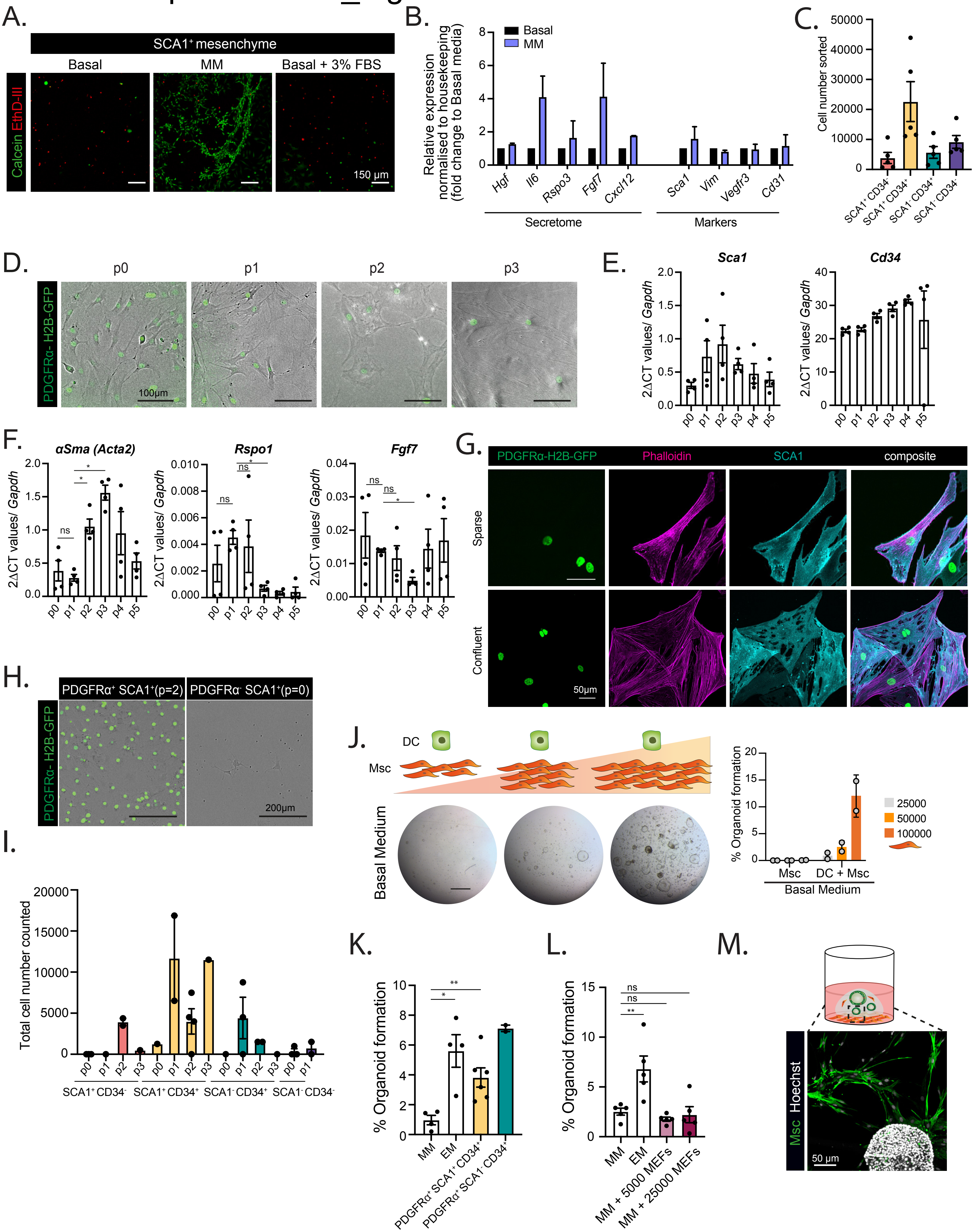

Figure S4. Growth and expansion of SCA1$^+$ mesenchymal cells in vitro. Related to Figure 4.

A) Cell viability assay indicates that SCA1$^+$ mesenchymal cells grow best in MM medium. SCA1$^+$ Msc cells were cultured within 3D Matrigel droplet in Basal (n=3), MM (n=4) and Basal + 3% FBS medium (n=3) and 6 days later cells were incubated with the cell viability dye calcein (4 μM, green) and the cell death dye EthD-III (8μM, red) and imaged using a fluorescence microscope. Composite pictures were obtained by merging the single channel images in FIJI/ImageJ.

B) Gene expression analysis of SCA1$^+$ Msc secretome and markers in presence or absence of 30% WNT 3a conditioned media in MM or Basal media supplemented with 3%FBS. Fold change normalized to basal media condition (n=2 biological and independent replicates).

C) Number of sorted cells from each isolation performed for the specified Msc subpopulations (PDGFRα$^+$SCA1$^+$CD34$^-$, red; PDGFRα$^+$SCA1$^+$CD34$^+$, yellow; PDGFRα$^+$SCA1$^-$CD34$^+$, green; PDGFRα$^+$SCA1$^-$CD34$^-$, purple)

D) Representative images of Msc cells in passage 0, passage1, passage 2 and passage 3 grown on plastic in MM showing brightfield and Pdgfra-H2BGFP expression (green). Composite pictures were obtained by merging the single channel images in FIJI/ImageJ.

E-F) mRNA expression of Sca1, Cd34, aSma, Rspo1 and Fgf7 by qRT-PCR analysis (n=4, from 2 independent experiments) of serially passaged SCA1$^+$ PDGFRα$^+$ cells. p-values were obtained using Mann-Whitney test, ns p> 0.1, * p=0.0286.

G) Representative images of Msc PDGFRα$^+$SCA1$^+$ cells in sparse and confluent culture conditions (passage 3), stained for marker SCA1 (cyan) and phalloidin (magenta) to visualize actin network. Nuclei from Pdgfra-H2B-GFP are also shown (green). Composite pictures were obtained by merging the single channel images in FIJI/ImageJ.

H) Brightfield and fluorescence images of PDGFRα$^+$SCA1$^+$ Msc following 2 serial passages (p2) of culture on plastic and with MM. Note that, PDGFRα$^-$SCA1$^+$ cells cannot be expanded under these culture conditions. Composite pictures were obtained by merging the single channel images in FIJI/ImageJ.

(I) Total cell number counted of the corresponding Msc populations counted at the time of passage after expansion.

(J) Organoid formation efficiency correlates with the number of mesenchymal cells in the co-culture. Increasing numbers of freshly sorted Msc cells were cultured alone or with EpCAM$^+$ DC in a 3D Matrigel droplet overlaid with medium w/o any growth factors (basal medium) and 10 days later organoid formation was assessed. Representative bright field images are shown. Scale bar, 100μm. Graph represents the mean ± SD of n=2 independent experiments.

K) Organoid formation efficiency assessed after 7 days of Matrigel droplet co-culture of 5000 freshly sorted ductal cells with 4000 mesenchymal cells of the specified fraction. p-values were obtained using Welch's test, p<0.01 (**), p<0.1 (*); n=5 for MM, EM and CD34$^+$ SCA1$^+$, and n=2 for CD34$^+$SCA1$^-$ cells.

L) Organoid formation efficiency assessed after 7 days of Matrigel droplet co-culture of 5000 freshly sorted ductal cells with 5000 or 25 000 MEF (mouse embryonic fibroblast) cells. P-values were obtained using Mann-Whitney test, p<0.01 (**), ns> 0.1.

(M) Representative image of an 8-day co-culture between ductal cells and mesenchymal cells (green) seeded within a 3D Matrigel droplet co-culture. Note that most of the mesenchymal cells attach to the bottom of the culture plate and spatially segregate from the DC-derived organoids, not establishing any cell-cell contact. Nuclei were counterstained with Hoechst (white). Composite pictures were obtained by merging the single channel images in FIJI/ImageJ.

Cordero-Espinoza et al._Figure S5

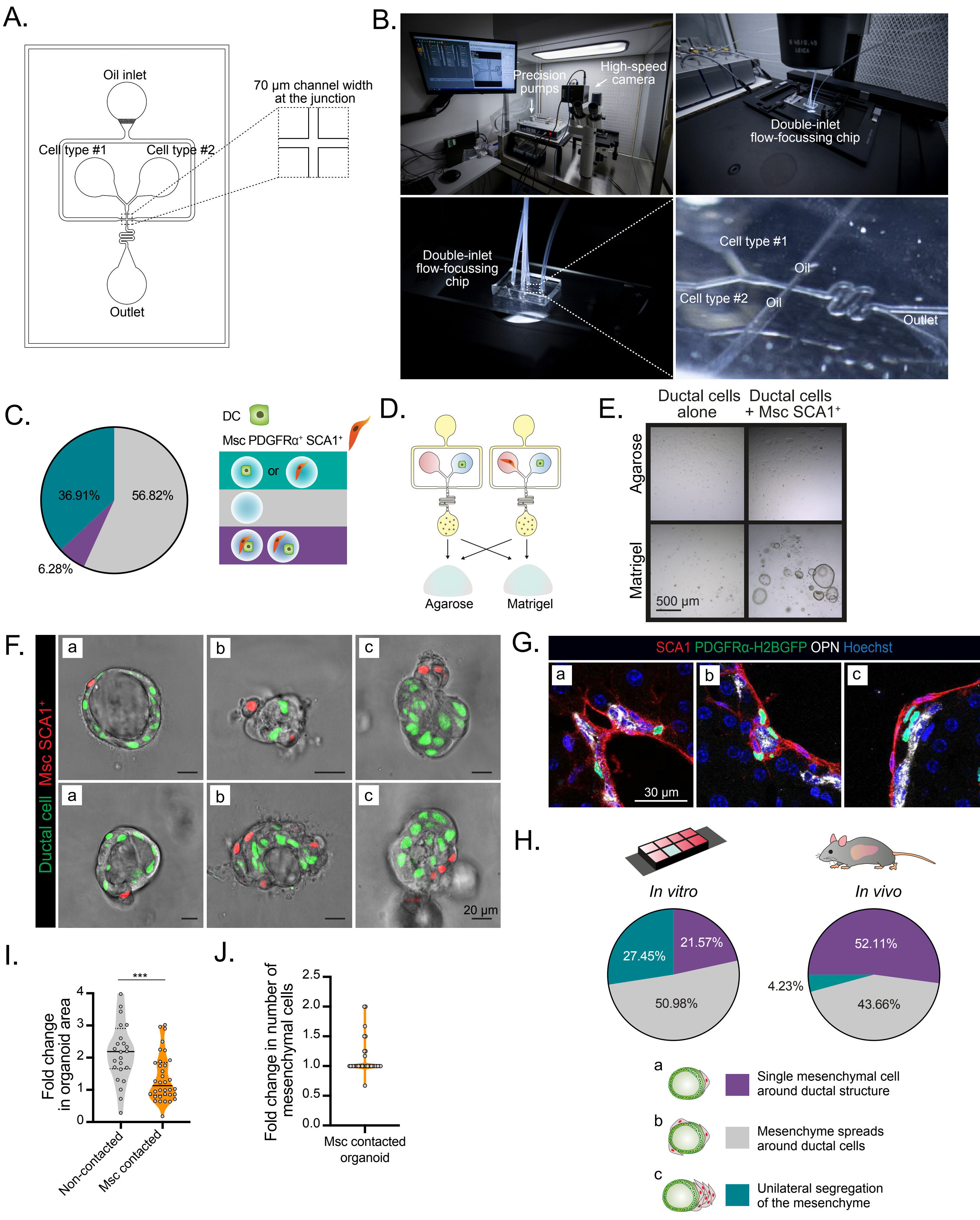

Figure S5. Msc-contacted organoids containing ductal and mesenchymal cells recapitulate in vitro the ductal : mesenchymal architecture of the portal tract. Related to Figure 5.

Organoid cells were encapsulated alone or with $SCA1^+$ Msc cells into agarose droplets using an FFD, seeded into 8-μ well dishes, imaged live for 24h at day 4 post-encapsulation and evaluated for the generation of organoids containing ductal and mesenchymal cells.

A) Design of the microfluidic double-inlet flow-focusing device (chip).

B) Pictures showing the microfluidic set up; precision pumps control the flow of liquids from syringes; high speed camera can monitor encapsulation in the double-inlet flow-focusing chip; close up of the chip; the whole apparatus is enclosed in a laminar flow hood.

C) Pie chart summarizing the frequency of microgels containing no cells (grey), only one cell-type (either DC or Msc $PDGFR\alpha^+SCA1^+$ cells, teal), or both cell types (purple) at time t=0 following microfluidic encapsulation. n=5 independent experiments were performed.

D-E) Agarose microgels were seeded into 8-μ well dishes containing a 3D Matrigel or agarose layer and cultured in MM. D) Scheme of the experimental design. E) Representative brightfield images of organoid formation. Note that organoids were only generated when agarose microgels were embedded in Matrigel following a co-encapsulation with ductal and mesenchymal cells (n=3).

F) Representative single z-stack snapshots of Msc-contacted organoids at d4 post microfluidic encapsulation showing different ductal-mesenchymal cell dispositions categorized as a (1 mesenchymal cell attached), b (mesenchymal cells spread on the periphery of the organoid) or c (mesenchymal cells segregated to one side of the organoid). Composite pictures were obtained by merging the single channel images in FIJI/ImageJ.

G) Representative single z-stack images of homeostatic Pdgfra-H2B-GFP (nuclear green) mouse livers co-stained with SCA1 (red), Osteopontin (OPN, white) and counter-stained with Hoechst (blue). Categories as in F: a (1 mesenchymal cell around ductal epithelium), b (mesenchymal cells spread around the ductal epithelium) or c (mesenchymal cells segregated to one side of the ductal epithelium).

H) Pie chart summarizing the array of ductal-mesenchymal cell dispositions in vitro (left) and in vivo (right) from n=3 independent experiments (n=51 organoids and n=85 bile duct structures). Composite pictures were obtained by merging the single channel images in FIJI/ImageJ.

I) Violin plot graph representing the data point distribution, median and IQR of fold changes in organoid area in mesenchyme-contacted and non-contacted structures within a 24h-period of time-lapse imaging at d4 following microfluidic encapsulation. P-value was obtained by Mann-Whitney test. ***, p=0.0006, n=3 independent experiments.

J) Violin plot graph representing the data point distribution, median and IQR of the fold change of mesenchymal cell numbers in Msc-contacted organoids within a 24h-period of time-lapse imaging at d4 following microfluidic encapsulation, n=3 independent experiments.

Cordero-Espinoza et al._Figure S6

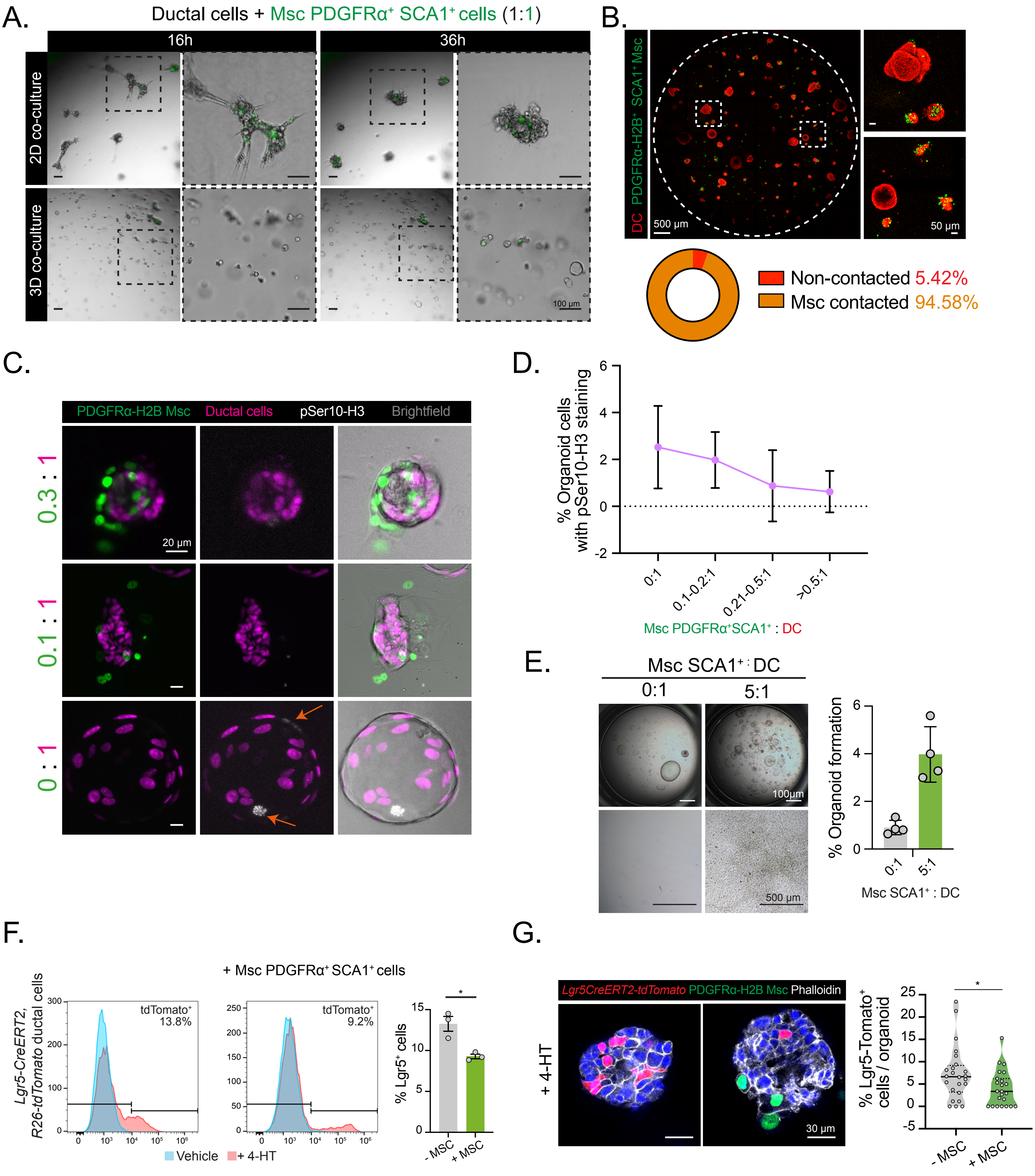

Figure S6. The ratio between ductal cells and mesenchymal cells in co-cultures determines the net outcome of ductal cell proliferation. Related to Figure 6.

A) Ductal cells (5,000 cells) from organoids were co-cultured with 5,000 PDGFRα-GFP$^+$ SCA1$^+$Msc (green, 1:1 ratio) in a 96-well plate by either culturing them on top of a well pre-coated with a Matrigel layer (top) or embedding them within a Matrigel droplet (bottom). Representative pictures at 16h and 36h after seeding are shown. Composite pictures were obtained by merging the single channel images in EVOS software.

B) Aggregation efficiency of nuclear tdTomato$^+$ DC (red) and PDGFRα-GFP$^+$ SCA1$^+$ Msc (green) seeded at a 1:1 ratio (5,000 cells each) on a Matrigel layer. Representative images of one of n=4 independent biological replicates are shown. Composite pictures were obtained by merging the single channel images in FIJI/ImageJ.

C-D) Mitoses decrease with increasing co-culture ratios of Msc to DCs. C) Msc-contacted (PDGFRα$^+$SCA1$^+$ Msc, nuclear green) and non-contacted organoids (nuclear magenta), stained for phospho-Serine10-Histone H3 (pSer10-H3, marker of mitosis, white); ratio of Msc to DC is specified for each structure; orange arrows point to dividing cells. Composite pictures were obtained by merging the single channel images in FIJI/ImageJ. D) Graph represents mean ± SEM of n=3 independent experiment quantification of detected pSer10-H3 staining in organoid structures has been divided into bins specified by the ratio of Msc to DC cells in each structure.

E) Freshly sorted EpCAM$^+$ DC were cultured in a transwell alone or with SCA1$^+$Msc at a 1:5 ratio for 8 days in MM. Note that in the absence of cell-cell contact, SCA1$^+$ mesenchymal cells do not inhibit ductal cell proliferation even at a >10-fold higher ratio (1:5) than the homeostatic ratio. Representative images of the top and bottom parts of the transwell are shown. Graph represents the quantification of organoid formation. Data is plotted as mean ± SD of n=3 independent experiments.

F) Single Lgr5CreERT2, R26-tdTomato ductal organoid cells were cultured alone or co-cultured with PDGFRα-GFP$^+$SCA1$^+$ Msc cells (1:1 ratio) on top of Matrigel and overlaid with EM + WNT3a CM medium. On day 3, cultures were incubated with 10μM of 4-hydroxytamoxifen (4-HT) and analyzed for percentage of tdTomato$^+$ organoid cells via flow cytometry 24h later. Graph represents the mean ± SD (n=3) of the number of Lgr5$^+$ cells quantified by FACS. Note that upon contact-permissive co-culture the number of Lgr5$^+$ cells is significantly reduced even in the presence of all growth factors and WNT3a ligand. P-value was obtained by Student t-test. *, p=0.0137.

G) PDGFRα-GFP$^+$ SCA1$^+$ Msc cells (geen) were co-cultured with single Lgr5CreERT2/R26-tdTomato liver organoid cells at a 1:0.5 ratio in complete EM + WNT3a CM medium in a 96-well plate. At day 3 of culture, cells were treated with 4-HT and fixed/stained 24h later. Representative single z-stack immunofluorescence images of same-well organoids counter-stained with Hoechst (blue) and Phalloidin (white). Violin plot graph represents the percentage of Lgr5-tdTomato$^+$ cells/organoid in Msc-Contacted vs non-contacted organoids. Note that in Msc-contacted organoids, where cell-cell contact is established, the number of Lgr5$^+$ cells is significantly reduced even in the presence of all growth factors and WNT3a ligand in the medium. P-value was obtained by Mann-Whitney test. *, p=0.0348 from n=23 organoids each from two independent experiments. Composite pictures were obtained by merging the single channel images in FIJI/ImageJ.

Cordero-Espinoza et al._Figure S7

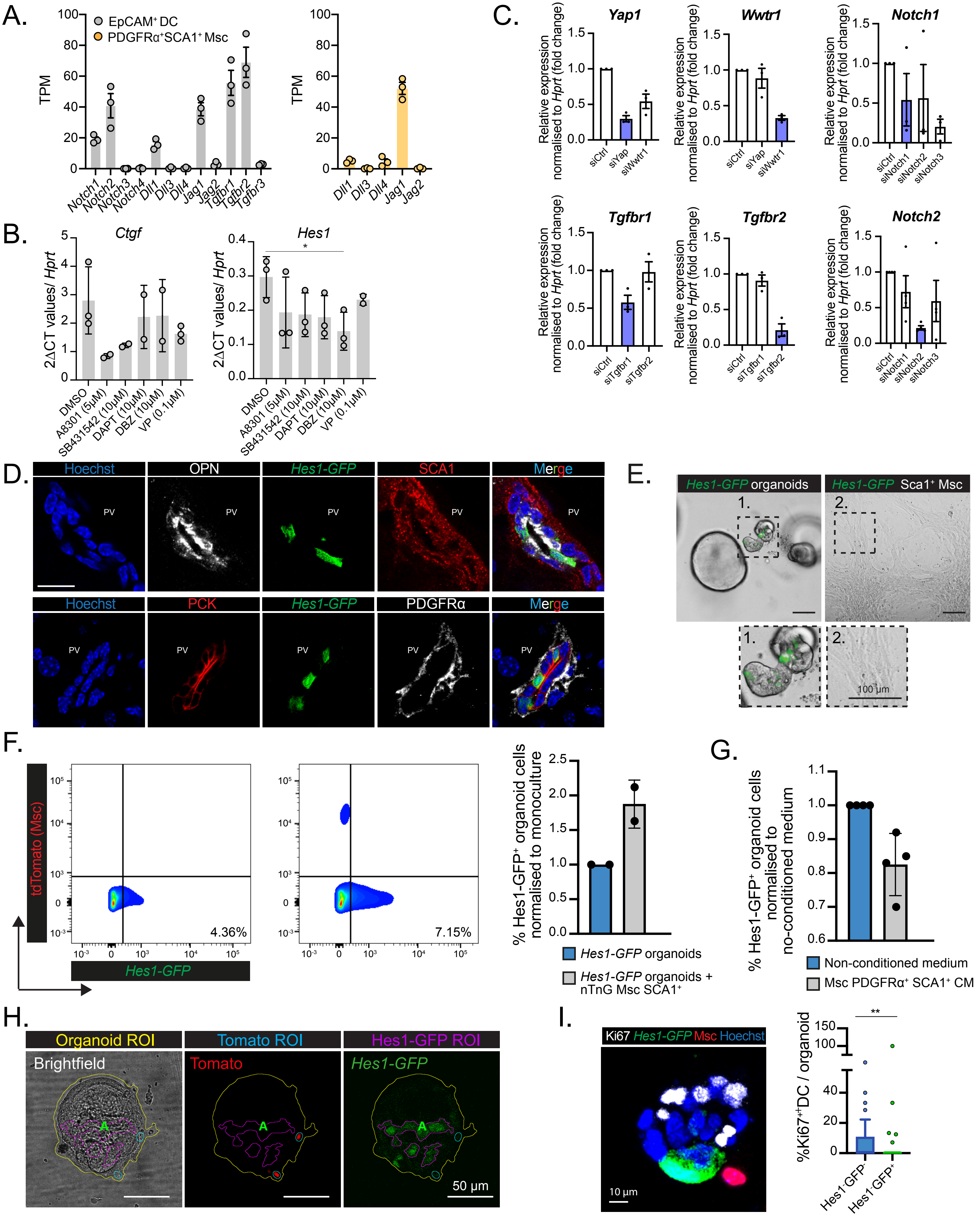

Figure S7. Cell-cell contact from PDGFRα^{+}SCA1^{+} Msc inhibits DC proliferation via Notch signaling. Related to Figure 7.

A) Gene expression analysis on selected genes of the Notch and Tgfb pathway in freshly sorted EpCAM^{+} DC (grey bars) and PDGFRα^{+}SCA1^{+} Msc cells (orange bars) subject to RNA sequencing. Graphs represent mean ± SEM of n=3 mice.

B) Ductal organoid cells were treated with DMSO, A8301 (5μM), SB431542 (10μM), DAPT (10μM), DBZ (10μM) or Verteporfin (VP, 0.1μM) for 24h and mRNA expression of Ctgf and Hes1 was measured via RT-qPCR. Graph shows mean ± SD of n=3 independent experiments. P values were calculated with a Student t-test. All treatments were compared to DMSO control. For Ctgf expression: A8301, p=0.1132 (ns); SB431542, p=0.1713 (ns); DAPT, p=6207 (ns); DBZ, p=0.6621 (ns); VP, p=0.1688 (ns). For Hes1 expression: A8301, p=0.2086 (ns); SB431542, p=0.0953 (ns); DAPT, p=0.0799 (ns); DBZ, p=0.0285 (*); VP, p=0.2419 (ns).

C) Ductal organoid cells (50,000) were transfected with siRNAs oligos against the indicated genes and efficiency of knockdown was assessed 24h later by determining the expression of the corresponding genes via RT-qPCR. Graph represents the mean ± SEM of n=3 biological replicates.

D) Single z-stack images of Hes1-GFP mouse livers immunostained against OPN (white) and SCA1 (red) (top) or PCK (red) and PDGFRα (white) (bottom) and counterstained with Hoechst. Note that Hes1-GFP expression (green) is restricted to the ductal compartment (OPN^{+} or PCK^{+} cells). Composite pictures were obtained by merging the single channel images in FIJI/ImageJ.

E) Ductal cells (DC) and SCA1^{+} Msc were isolated from Hes1-GFP mice and cultured in EM and MM respectively. Representative images of GFP fluorescence are shown (DC, n=4; SCA1+ Msc n=2). Composite pictures were obtained by merging the single channel images in EVOS software.

F) Single Hes1-GFP ductal organoid cells were cultured alone (left panel) or with nuclear tdTomato^{+} SCA1^{+} Msc cells at 1:1 ratio in growth factor rich medium (EM + WNT3a CM medium, right panel) and on top of a well coated with a layer of Matrigel. On day 8, the cultures were analyzed for Hes1-GFP expression via flow cytometry. Graph presents mean ± SD of the number of Hes1-GFP^{+} organoid cells in co-culture vs monocultures. n=2 independent experiments.

G) Single Hes1-GFP ductal organoid cells were cultured in conditioned media from PDGFRα^{+}SCA1^{+} Msc cells or non-conditioned media control (refreshed every 48h) for 8 days. On day 8, the cultures were analyzed for Hes1-GFP expression via flow cytometry. Graph presents Mean ± SD of the number of Hes1-GFP^{+} cells (n=4).

H) Representative signal masks generated by a custom-made ImageJ script detecting organoid area (yellow outline), nuclear tdTomato fluorescence (cyan outline) and Hes1-GFP fluorescence (purple outline) in single z-stack images of Hes1-GFP organoid cells co-cultured with nuclear tdTomato^{+} SCA1^{+} Msc cells at 1:0.5 ratio (DC:Msc) in mesenchymal medium (MM).

I) Ki67 immunostaining (white) in 5-day Matrigel co-cultures between Hes1-GFP organoid cells (green) and nuclear tdTomato^{+} SCA1^{+} Msc cells (red, seeded at 1:0.5 DC:Msc) (left). Quantification of the percentage of Ki67^{+} cells in Hes1-GFP^{-} vs Hes1-GFP^{+} ductal cells per organoid. Tukey box plot displaying the median and IQR (n=3). p=0.0076, Mann Whitney test (right). Composite pictures were obtained by merging the single channel images in FIJI/ImageJ.
